# Unveiling the role of boroxines in metal-free carbon–carbon homologations using diazo compounds and boronic acids†
†Electronic supplementary information (ESI) available. See DOI: 10.1039/c7sc02264f, additional data is available from the University of Cambridge Data Repository Website: https://doi.org/10.17863/CAM.10823.


**DOI:** 10.1039/c7sc02264f

**Published:** 2017-06-15

**Authors:** Claudio Bomio, Mikhail A. Kabeshov, Arthur R. Lit, Shing-Hing Lau, Janna Ehlert, Claudio Battilocchio, Steven V. Ley

**Affiliations:** a Department of Chemistry , University of Cambridge , Lensfield Road , Cambridge CB21EW , UK . Email: svl1000@cam.ac.uk ; http://www.leygroup.ch.cam.ac.uk

## Abstract

By means of computational and experimental mechanistic studies the fundamental role of boroxines in the reaction between diazo compounds and boronic acids was elucidated. Consequently, a selective metal-free carbon–carbon homologation of aryl and vinyl boroxines using TMSCHN_2_, giving access to TMS-pinacol boronic ester products, was developed.

## Abstract 2

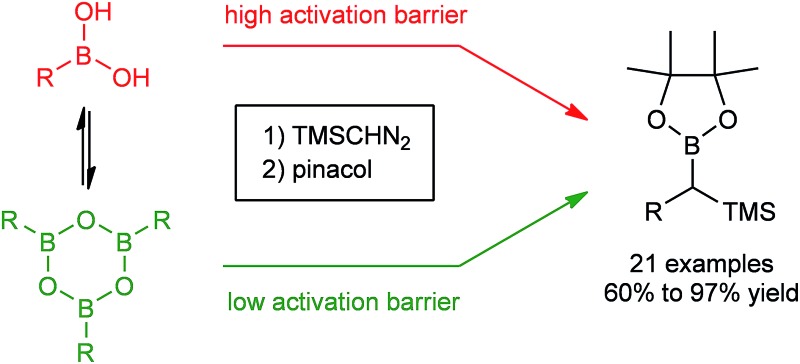
By means of computational and experimental mechanistic studies the fundamental role of boroxines in the reaction between diazo compounds and boronic acids was elucidated.

## Introduction

Carbon–carbon bond forming reactions have been at the centre of organic synthesis for decades allowing efficient and quick assembly of complex molecular structures.^1^–^6^ Among many others, organoboron compounds are often the reagents of choice as they promote C–C bond formation reactions with high chemo- and stereoselectivity without the need for expensive, sometimes unstable and toxic transition metal catalysts.^7^,^8^ A large number of highly efficient methods have been described where a boron atom plays initially a role of the Lewis acid followed by a σ-bond migration thus allowing a metal-free coupling reaction between an electrophile (organoborane, organoboronic ester, dihaloborane)^9^–^13^ and a nucleophile (organolithium intermediates, sulfur ylides, diazo compounds).^9^,^14^–^23^

Coupling between boronic acids and diazo compounds has attracted considerable interest in the synthetic community as boronic acids are readily available, usually stable and more atom efficient reagents when compared to their ester analogues.^16^,^24^–^26^ Additionally, the homologation using TMS-diazomethane (TMSCHN_2_) and organoboron compounds is useful to install a trimethylsilyl and boron functionality in a metal-free fashion.^27^–^32^ These doubly functionalised carbon substrates are of interest, as they may be used to generate sequential or orthogonal functionalization at the same carbon atom.^28^,^29^ To date, the homologation with TMSCHN_2_ has only been performed in combination with boronic acids to access the TMS-free homologation products.^26^,^33^ While several proposed mechanisms have been reported, the role of a boronic acid anhydride (boroxine) intermediate in these reactions remains unclear.^16^,^26^,^33^

In this work, we report a detailed computational and experimental study unveiling the crucial role of the boronic acid anhydrides–boroxines in these coupling reactions with diazo compounds and demonstrate how this knowledge can be applied to improve the scope of the method.

## Results and discussion

**Scheme 1:**
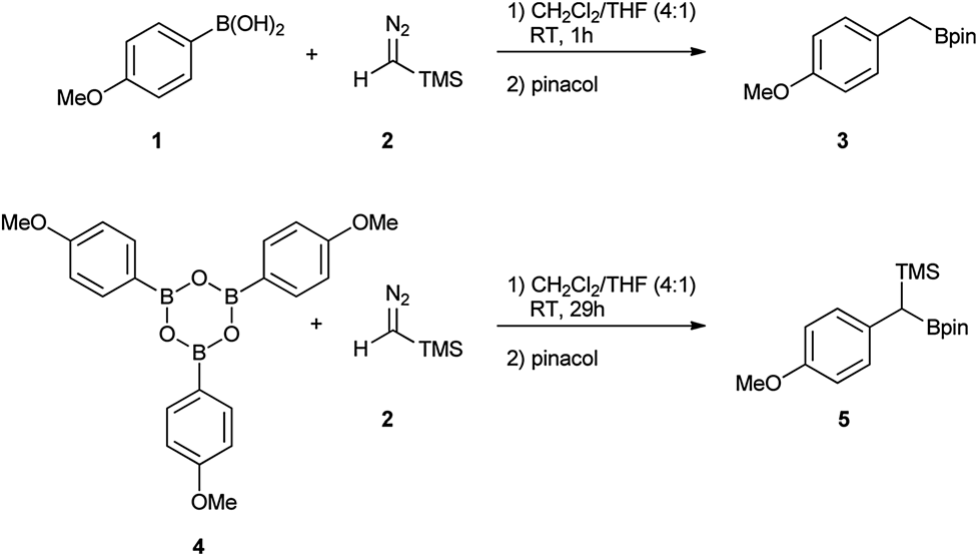
Reactions of the boronic acid **1** and boroxine **4** with TMS diazomethane **2**.

Initially, the intriguing difference between the behaviour of the *p*-methoxyphenyl boronic acid (**1**) and the respective *p*-methoxyphenylboroxine (**4**) in their reactions with TMS diazomethane (TMSCHN_2_, **2**) was noteworthy (Scheme 1).

When mixing the boronic acid **1** with TMSCHN_2_**2** at ambient temperature, nearly instant decolourisation of the reaction mixture (originating from TMSCHN_2_) and strong gas evolution was observed. As a result, the formation of a roughly 1 : 1 mixture of homologation product **3** and Bpin ester of the starting material **6** was isolated. By contrast, when using boroxine **4** instead of boronic acid **1** under the same conditions, no gas evolution and only very slow decolourisation was observed. As expected, the TMS-homologated product **5** was isolated as the main product in 75% yield after 29 h of stirring at RT (Scheme 1).

**Fig. 1:**
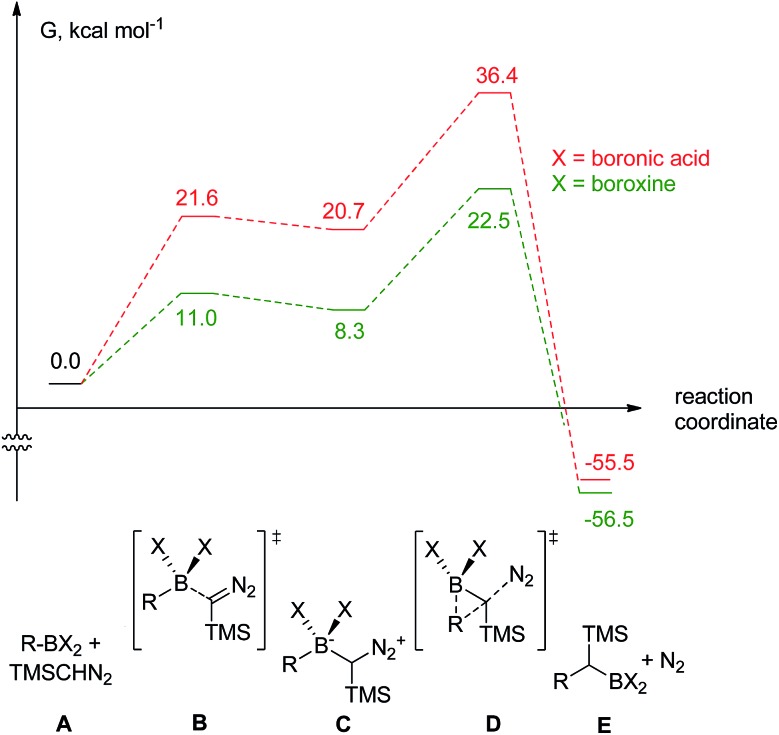
The computed reaction pathway for the coupling between boronic species and TMS diazomethane (R = Ph).

The decomposition of TMSCHN_2_ (**2**) to diazomethane is known to occur under acidic conditions and is due to the formation of H_3_O^+^ through an equilibrium between boronic acids or boroxines and their anionic tetrahedral species in the presence of water. Thereby this decomposition can rationalise the difference in the products obtained (Scheme 1).^34^,^35^ In order to further understand the role of boroxines in the coupling of diazo compounds with boronic acids, quantum chemistry calculations at density functional theory (ωB97xD/cc-pVTZ//ωB97xD/cc-pVDZ with SMD solvation model (solvent = dichloromethane, *ε* = 8.93) as implemented in Gaussian 09) level were therefore performed (Fig. 1).^36^–^42^

It was found that both reactions, using boronic acid or boroxine in combination with TMSCHN_2_ (**2**), proceed *via* a two-step mechanism involving a sequence of a coordination transition state (point B, Fig. 1), unstable intermediate (point C, Fig. 1) and the highest energy point on the coordinate for both examples, which corresponds to the transition state for migratory insertion (point D, Fig. 1). In contrast to other reactions involving boronic acids and boroxines, where similar reactivity for both is usually observed,^34^ the striking difference of 13.9 kcal mol^–1^ (36.4 *vs.* 22.5, respectively; Fig. 1) for the activation Gibbs energies was computed. According to these computational results, only the boroxine, not the boronic acid, is expected to be reactive towards TMSCHN_2_ (**2**) (the barrier of 36.4 kcal mol^–1^ cannot be achieved under conventional conditions of organic reactions in solution).^43^,^44^

In order to check whether similar differences in reactivity are expected for reactions of diazomethane with boroxines and boronic acids, additional calculations using the same computational model, were performed. It can be concluded from the analysis that diazomethane should behave similarly to TMSCHN_2_ (**2**) and react smoothly with boroxines (*G*_act_ = 18.5 kcal mol^–1^). However, the reaction between diazomethane and boronic acids might be possible, but would require significantly higher activation energy (*G*_act_ = 29.2 kcal mol^–1^). Additionally, only mono addition of TMSCHN_2_ (**2**) to boroxine is expected due to the significantly higher activation barrier (27.2 kcal mol^–1^, lower diastereomeric transition state) for the second insertion into the benzylic boroxine intermediate (Fig. 1E).

To verify the computed results, control experiments were performed (Table 1). As already previously described, when using boronic acid **1**, a fast decomposition of TMSCHN_2_ (**2**) occurred with formation of the homologated product **3** and only traces of the TMS homologation product **5** being observed. The conversion of the starting material **1** into **3** using 1.03 eq. of TMSCHN_2_ (**2**) gave only a 43% yield (entry 1). In contrast, when using boroxine (**4**) and 3.10 eq. of TMSCHN_2_ (**2**) (1.03 eq. per boron) the exclusive formation of the TMS homologation product **5** was observed (75% yield, entry 2). Addition of DIPEA to stabilise TMSCHN_2_ (**2**) led to much slower generation of diazomethane but had no effect on the final product distribution (entries 3 and 5). When water was added to the reaction (in order to shift the equilibrium towards the boronic acid **1**) lower conversion towards homologation products **3** and **5** was observed, regardless of the presence of DIPEA. Moreover, it can be observed that water has the effect to increase the rate of decomposition of TMSCHN_2_ (entries 4, 6 and 7). Finally, the addition of large excess of base using boroxine **5** significantly decreases the reaction rate (entry 8). This can also explain why only small amount of TMS homologation product **5** is observed when boronic acid **1** is used in presence of DIPEA at room temperature (entries 3 and 5).

**Table 1:** Experimental homologation studies with boronic acid **1** and boroxine **4** using TMSCHN_2_ (**2**)

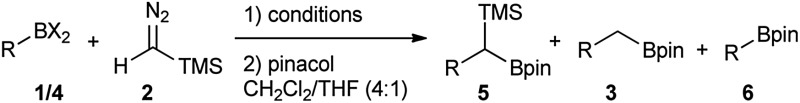						
Entry	R-BX_2_	TMSCHN_2_	Time^*b*^	DIPEA	H_2_O	Yield^*a*^ (5 : 3 : 6)
1	RB(OH)_2_	1.03 eq.	1 h	—	—	1% : 43% : 48%
2	(RBO)_3_	3.10 eq.	29 h	—	—	75% : 0% : 10%
3	RB(OH)_2_	1.03 eq.	15 h	2.1 eq.	—	2% : 40% : 51%
4	RB(OH)_2_	1.03 eq.	10 h	2.1 eq.	2.0 eq.	0% : 14% : 83%
5	RB(OH)_2_	1.03 eq.	65 h	6.0 eq.	—	2% : 42% : 47%
6	RB(OH)_2_	1.03 eq.	23 h	6.0 eq.	2.0 eq.	0% : 16% : 80%
7	RB(OH)_2_	1.03 eq.	1 h	—	2.0 eq.	0% : 19% : 78%
8	(RBO)_3_	3.10 eq.	67^*c*^ h	6.0 eq.	—	80% : 0% : 17%

^*a*^NMR yields. R = 4-MeOPh.

^*b*^Time required for full disappearance of TMSCHN_2_.

^*c*^TMSCHN_2_ not fully consumed.

**Fig. 2:**
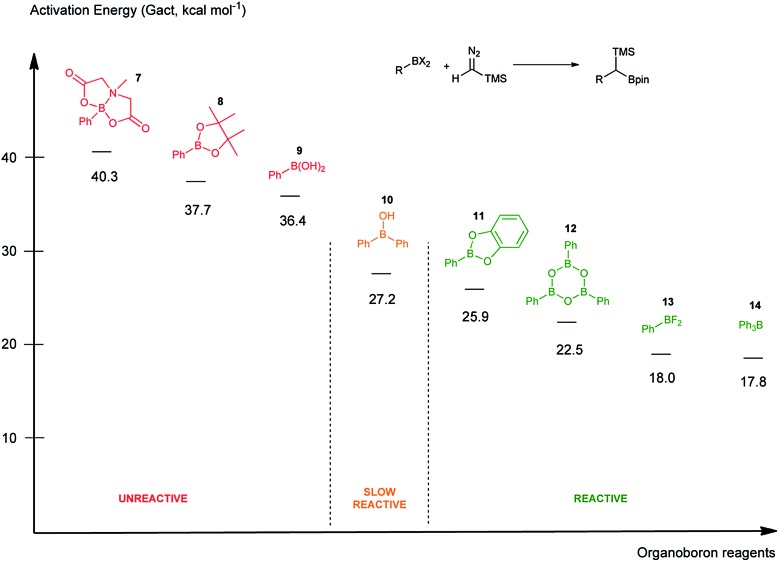
Computed reactivities of organoboron reagents towards TMS diazomethane.

To rank organoboron reagents by their reactivity towards TMSCHN_2_ (**2**), further computational studies were performed and the results were subsequently compared with reactivity trends previously reported in the literature (Fig. 2). From all the computed examples, organoborane **14** showed the highest reactivity towards TMSCHN_2_, followed by difluoroorganoborane **13**, boroxine **12** and catecholborane **8**. Organoboranes are known to react fast with TMSCHN_2_ (**2**) at –78 °C while catecholboranes need heating (60 °C) over an extended time period (12 h).^27^,^45^ Both the reported reactivities as well as the experimentally observed behaviour of boroxines are in good correlation with our calculations. To prove that both the MIDA **7** and pinacol boranes **8** are not reactive, the substrates were treated with TMSCHN_2_ (**2**) at reflux in toluene for 3 d. In all cases, no homologation products were observed. Again, this is in agreement with our calculations.

To conclude therefore, it was determined that boroxines are ideal reagents for reactions with diazo compounds, as they allow excellent reactivity and reasonable atom economy. Additionally, these results corroborate the fact that boroxines are most likely the reactive species in the coupling between diazo compounds and boronic acids.^26^

Our studies continued with the optimisation of reaction conditions by using boroxines, obtained *via* dehydration of boronic acids using a Dean–Stark apparatus (Table 2).

**Table 2:** Optimisation of the TMS homologation with boroxines^*a*^

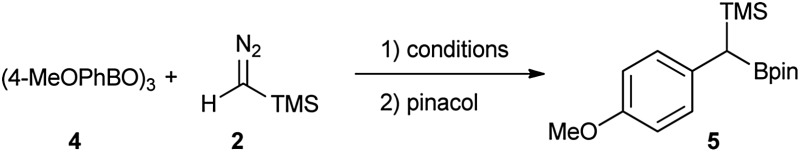					
Entry	Solvent	*T*	Time	DIPEA	Yield
1	CH_2_Cl_2_/THF (4 : 1)	24 °C	29 h	—	75^*b*^ ^,^^*c*^%
2	Toluene	60 °C	3 h	—	78^*c*^%
3	Toluene	60 °C	3 h	3.0 eq.	88%
4	Toluene	85 °C	1 h	3.0 eq.	87%
5	Toluene	85 °C	1 h	3.6 eq.	93%
6	Toluene	85 °C	1 h	3.6 eq.	50^*b*^ ^,^^*d*^%

^*a*^1.03 eq. TMSCHN_2_ per boron atom.

^*b*^NMR yield.

^*c*^Aldehyde formation observed.

^*d*^Reaction performed with boronic acid **1**.

As already described in Scheme 1, the desired product **5** could be obtained in 75% yield after stirring at rt for 29 h. To our delight, it was found that, unlike in other reported homologation reactions with TMSCHN_2_ (**2**), using just a slight excess of 0.033 equiv. of TMSCHN_2_ (**2**) per boron atom was sufficient to reach full conversion.^26^,^33^ To speed up the process, the reaction was performed at 60 °C in toluene, to give 78% of the desired product (**5**) after just 3 h (Table 2, entry 2). As a side product, the corresponding aldehyde was observed (approximately 10%), which is likely to arise from the oxidation of the TMS-boroxine intermediate. It was possible to suppress the formation of this byproduct by adding DIPEA to the reaction mixture, which resulted in an improved yield of 88% for compound **5** (Table 2, entry 3). Increasing the temperature to 85 °C led to full conversion after 1 h without affecting the yield. By increasing the amount of DIPEA from 3.00 equiv. to 3.60 equiv. the yield of the homologation could be further improved to 93%. Further attempts to increase the yield by adding more DIPEA did not lead to any improvements. Finally, applying the optimised conditions to the boronic acid **1** led to the formation of the desired TMS homologated product **5** in moderate yield (50%) which can be attributed to the very poor solubility of boronic acid and water in toluene and easier formation of the boroxine at elevated temperature (entry 6).

**Scheme 2:**
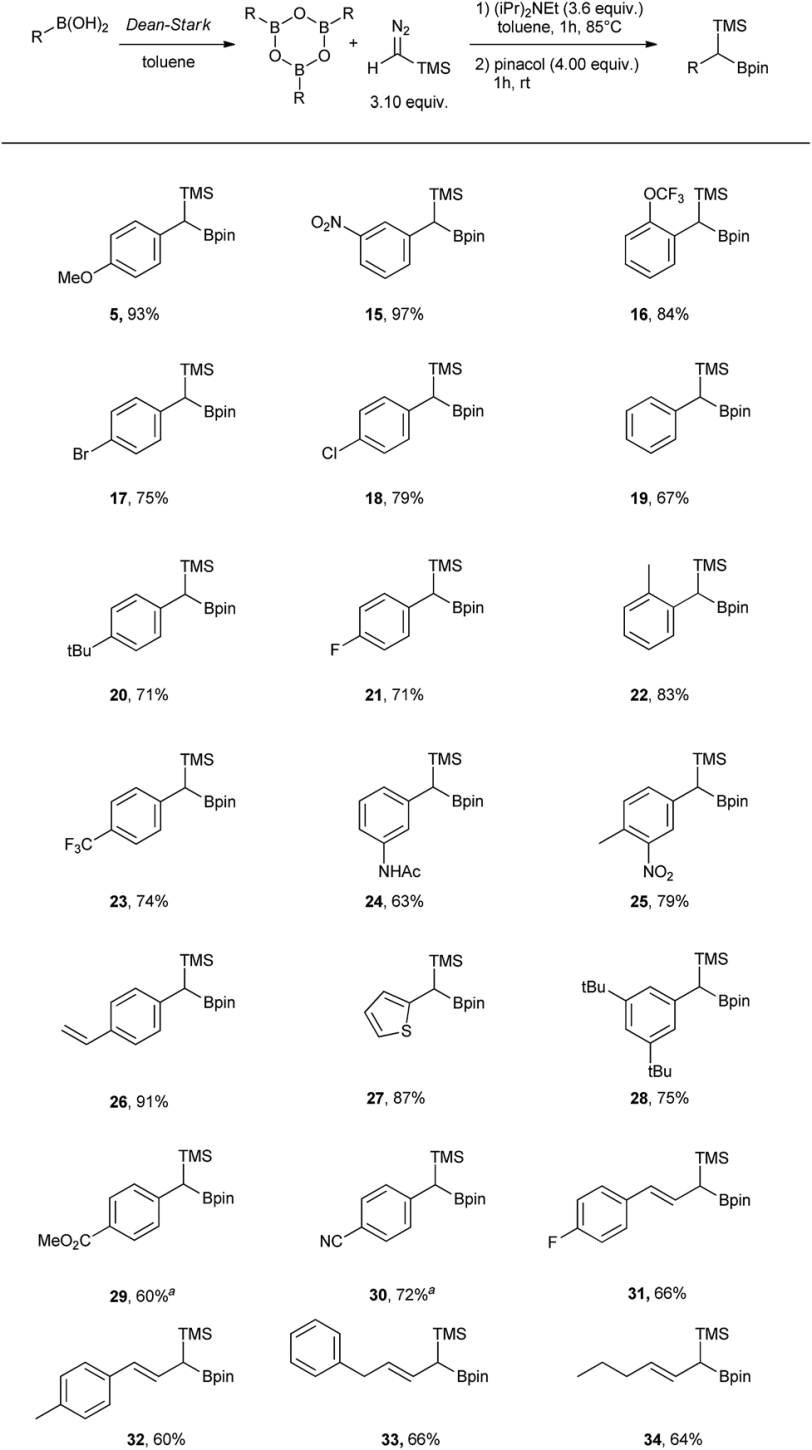
Scope table of the homologation reaction using TMSCHN_2_ and boroxines. ^*a*^ Reaction performed without DIPEA. General comment: TMS-boroxine intermediates are prone to protodeboration and oxidation and can therefore not be isolated.

Having the optimal conditions in hand, the scope was expanded to other boronic acids (see Scheme 2). The reaction showed broad functional group tolerance (–NO_2_, halogen, ester, amide, thiophene). The conditions could also be applied to vinyl boronic acid precursors (**31** to **34**). Significant difference in reactivity could be observed with substrates bearing strongly electron withdrawing groups in *para* position (**29** and **30**), where the use of DIPEA led to the protodeborated byproducts. In order to suppress protodeboration, the reaction with electron poor substrates had to be performed without the presence of base, with unavoidable 10–15% aldehyde formation as a side product.

**Fig. 3:**
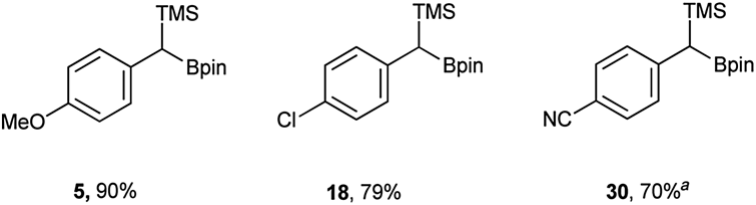
Yields of scaled up reactions (0.1 mol). ^*a*^ Reaction performed without DIPEA.

In addition to the scope, the homologation was performed on a gram-scale with three boronic acids (5, 18, 30) in order to demonstrate the practical usefulness of this reaction. As shown in Fig. 3 the homologation proceeded smoothly on scale by applying identical reaction conditions.

## Conclusions

We have shown experimentally and by means of DFT computations that boroxines of the corresponding boronic acids are likely to be the reactive intermediates in the homologation reaction with diazo compounds. Consequently, we have developed a metal-free, robust and scalable approach towards bench stable benzyl and allyl TMS-Bpin products with high functional group tolerance using TMSCHN_2_ and boroxines. Current investigations are directed towards the selective functionalisation of the TMS-Bpin products.

## Experimental

### General procedure for preparation of TMS-Bpin products

The reaction was carried out in dry conditions under an atmosphere of argon. To a mixture of boroxine (0.15 mmol, 1.0 equiv.) and *N*,*N*-diisopropylethylamine (0.094 mL, 0.54 mmol, 3.6 equiv.) in toluene (0.75 mL) was added (trimethylsilyl)diazomethane (0.23 mL, 0.465 mmol, 2 M in hexanes, 3.1 equiv.). The reaction mixture was stirred at 85 °C for 1 h and allowed to cool down to room temperature. Pinacol (70.9 mg, 0.60 mmol, 4.0 equiv.) was added and the reaction mixture was stirred at room temperature for 2 h. The reaction was quenched with a saturated aqueous solution of NH_4_Cl and the aqueous phase was extracted with EtOAc. The combined organic extracts were washed with brine, dried (MgSO_4_) and concentrated *in vacuo*. The crude residue was purified by silica gel flash column chromatography to afford the desired TMS-Bpin product.

## Supplementary Material

Supplementary information
