# Supplementary material for: Unveiling the role of boroxines in metal-free carbon–carbon homologations using diazo compounds and boronic acids
Source: Chem Sci. 2017 Jun 15;8(9):6071–5. doi: 10.1039/c7sc02264f (PMC5859889; doi:10.1039/c7sc02264f)
Supplement: Supplementary file 1 [file SC-008-C7SC02264F-s001.pdf]

Electronic Supplementary Material (ESI) for Chemical Science.
This journal is © The Royal Society of Chemistry 2017

Contents

1. General Experimental Details 1

2. Synthetic Procedure and Characterisation of Boroxines 3

3. Synthetic Procedures and Characterisation of TMS-Bpin 14

4. ^{1}H and ^{13}C NMR Spectra of Boroxines 30

5. ^{1}H and ^{13}C NMR Spectra of TMS-Bpin 38

6. Computational methods 59

7. References..99

1. General Experimental Details

All reactions were performed using oven-dried glassware (200 ˚C) under an atmosphere of argon unless otherwise stated. Solvents were dried and distilled using standard methods. All boronic acids were purchased at the highest commercial quality from Sigma Aldrich, Alfa Aesar and Fluorochem. (Trimethylsilyl)diazomethane was obtained from Sigma Aldrich. All reagents were used without further purification.

Analytical thin layer chromatography (TLC) was performed using silica gel 60 F_{254} pre-coated glass backed plates and visualized by ultraviolet radiation (254 nm) and/or Seebach reagent (12.5 g phosphomolybdic acid, 5.0 g cerium(IV)sulfate tetrahydrate, 16.0 mL water, 450.0 mL conc. sulfuric acid). Flash column chromatography was performed using high-purity grade silica gel (Merck grade 9385) with a pore size 60 Å and 230–400 mesh particle size under air pressure.

^{1}H NMR spectra were recorded on a 400 MHz DPX-400 Dual Spectrometer or a 600 MHz Avance 600 BBI Spectrometer as indicated. Chemical shifts are reported in ppm with the resonance resulting from incomplete deuteration of the solvent as the internal standard ($CDCl_3$: 7.26 ppm; $(CD_3)_2SO$: 2.50 ppm). ^{13}C NMR spectra were recorded on a 600 MHz Avance 600 BBI Spectrometer with complete proton decoupling. Chemical shifts are reported in ppm with the solvent resonance as the internal standard ($^{13}CDCl_3$: 77.16 ppm; $(^{13}CD_3)_2SO$: 39.52 ppm). Data are reported as follows: chemical shift δ/ppm, multiplicity (s = singlet, d = doublet, t = triplet, q = quartet, qn = quintet, br = broad, m = multiplet or combinations thereof; ^{13}C signals are singlets unless otherwise stated), coupling constants *J* in Hz, integration (^{1}H only). ^{1}H NMR signals are reported to 2 decimal places and ^{13}C signals to 1 decimal place unless rounding would produce a value identical to another signal. In this case, an additional decimal place is reported for both signals concerned.

Infrared spectra were recorded neat as thin films or as solids on a Perkin-Elmer Spectrum One FTIR spectrometer and selected peaks are reported.

High resolution mass spectrometry (HRMS) was performed using positive electrospray ionisation (ESI+), on either a Waters Micromass LCT Premier spectrometer or performed by the Mass Spectrometry Service for the Chemistry Department at the University of Cambridge. All m/z values are reported to 4 decimal places and are within ± 5 ppm of theoretical values.

Melting points were measured on a Stuart Scientific SMP3 melting point apparatus using a gradient of 0.5 °C.min^{-1}.

2. Synthetic Procedure and Characterisation of Boroxines

General Procedure A for Preparation of Boroxines: A mixture of the boronic acid (0.5 g) in toluene (10 mL) was refluxed for 5-7 h with the use of a Dean-Stark trap. The solvent was removed *in vacuo* and the residue dried overnight at 60 ˚C under high vacuum to yield the corresponding boroxine. This boroxine was directly used in the next step without further purification.

2,4,6-tris(4-methoxyphenyl)-1,3,5,2,4,6-trioxatriborinane (4):

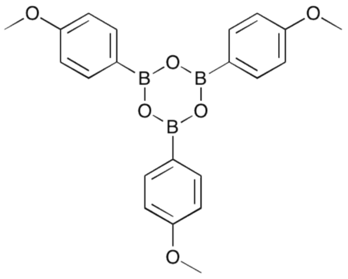

Prepared according to general procedure A using 4-methoxyphenylboronic acid to give the title compound as a white solid.

^{1}H NMR (600 MHz, $CDCl_3$): δ 8.17 (d, J = 8.6 Hz, 6 H), 7.02 (d, J = 8.6 Hz, 6 H), 3.89 (s, 9 H). Data consistent with reported compound.[1]

2,4,6-tris(3-nitrophenyl)-1,3,5,2,4,6-trioxatriborinane (15a):

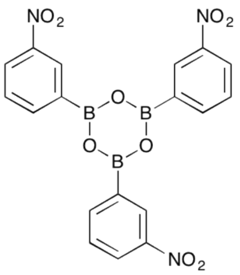

Prepared according to general procedure A using 3-nitrophenylboronic acid to give the title compound as a beige solid, which was insoluble in a variety of dry, deuterated NMR solvents.[3]

FTIR (ν_{max}, cm^{-1}): 3333, 1607, 1578, 1488, 1449, 1362, 1308, 1258, 1226, 1154, 1069, 1019, 927, 811.

m.p.: >277 ˚C (Decomposition) (lit. 280-281 ˚C).[6]

2,4,6-tris(2-(trifluoromethoxy)phenyl)-1,3,5,2,4,6-trioxatriborinane (16a):

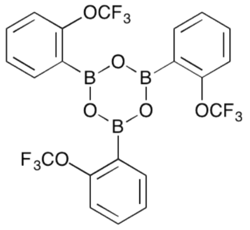

Prepared according to general procedure A using 2-(trifluoromethoxy)phenylboronic acid to give the title compound as a white solid.

^{1}H NMR (600 MHz, CDCl$_3$): δ 8.29 (d, *J* = 7.1, 3 H), 7.64-7.61 (m, 3 H), 7.44 (t, *J* = 7.3 Hz, 3 H), 7.36 (d, *J* = 8.0 Hz, 3 H).

^{13}C NMR (150 MHz, CDCl$_3$): δ 154.9 (d, *J* = 1.7 Hz), 138.5, 134.4, 126.8, 121.4, 120.8 (q, *J* = 254.9 Hz).

^{11}B NMR (128 MHz, CDCl$_3$): δ 28.4.

FTIR (ν_{max}, cm^{-1}): 3333, 1607, 1578, 1488, 1449, 1362, 1308, 1258, 1226, 1154, 1069, 1019, 927, 811.

HRMS (ESI): calculated for $C_{21}H_{12}O_6F_9{}^{11}B_3$ $[M]^+$ 564.0769, found 564.0778.

m.p.: 109-112 ˚C.

2,4,6-tris(4-bromophenyl)-1,3,5,2,4,6-trioxatriborinane (17a):

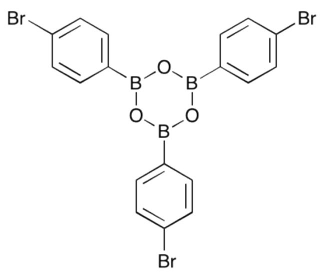

Prepared according to general procedure A using 4-bromophenylboronic acid to give the title compound as a white solid.

^{1}H NMR (600 MHz, $(CD_3)_2SO$): δ 7.81 (d, *J* = 8.1 Hz, 6 H), 7.57 (d, *J* = 8.1 Hz, 6 H).

^{13}C NMR (150 MHz, $(CD_3)_2SO$): δ 135.6, 130.5, 123.6.

^{11}B NMR (128 MHz, $(CD_3)_2SO$): δ 17.0.

FTIR (ν_{max}, cm^{-1}): 2920, 2851, 1662, 1587, 1559, 1428, 1392, 1363, 1349, 1307, 1295, 1259, 1176, 1104, 1080, 1063, 1010, 839, 826.

HRMS (ESI): Mass not found.

m.p.: >293 ˚C (Decomposition).

2,4,6-tris(4-chlorophenyl)-1,3,5,2,4,6-trioxatriborinane (18a):

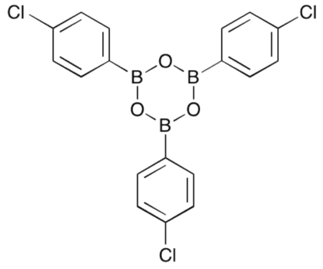

Prepared according to general procedure A using 4-chlorophenylboronic acid to give the title compound as a white solid.

^{1}H NMR (400 MHz, $CDCl_3$): δ 8.13 (d, *J* = 8.4 Hz, 6 H), 7.49 (d, *J* = 8.4 Hz, 6 H). Data consistent with reported compound.[1]

2,4,6-triphenyl-1,3,5,2,4,6-trioxatriborinane (19a):

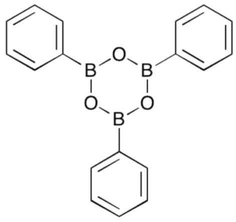

Prepared according to general procedure A using phenylboronic acid to give the title compound as a white solid.

^{1}H NMR (400 MHz, CDCl$_3$): δ 8.27-8.24 (m, 6 H), 7.63-7.59 (m, 3 H), 7.54-7.50 (m, 6 H). Data consistent with reported compound.[1]

2,4,6-tris(4-(*tert*-butyl)phenyl)-1,3,5,2,4,6-trioxatriborinane (20a):

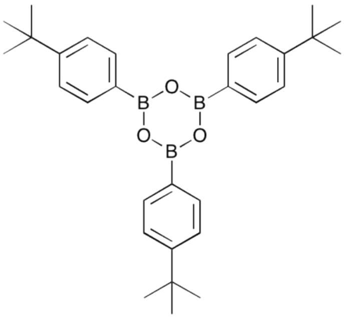

Prepared according to general procedure A using 4-*tert*-butylphenylboronic acid to give the title compound as a white solid.

^{1}H NMR (400 MHz, CDCl$_3$): δ 8.19 (d, *J* = 8.4 Hz, 6 H), 7.56 (d, *J* = 8.4 Hz, 6 H), 1.40 (s, 27 H). Data consistent with reported compound.[3]

2,4,6-tris(4-fluorophenyl)-1,3,5,2,4,6-trioxatriborinane (21a):

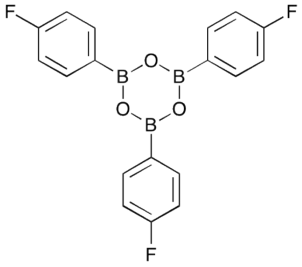

Prepared according to general procedure A using 4-fluorophenylboronic acid to give the title compound as a white solid.

^{1}H NMR (600 MHz, CDCl$_3$): δ 8.23-8.21 (m, 6 H), 7.21-7.18 (m, 6 H). Data consistent with reported compound.[4]

2,4,6-tri-*o*-tolyl-1,3,5,2,4,6-trioxatriborinane (22a):

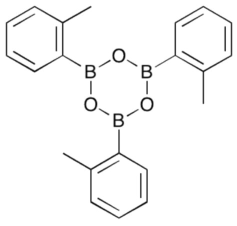

Prepared according to general procedure A using *o*-tolylboronic acid to give the title compound as a white solid.

^{1}H NMR (600 MHz, $CDCl_3$): δ 8.22 (d, *J* = 7.4 Hz, 3 H), 7.46 (t, *J* = 7.4 Hz, 3 H), 7.33-7.28 (m, 6 H), 2.83 (s, 9 H). Data consistent with reported compound.[1]

2,4,6-tris(4-(trifluoromethyl)phenyl)-1,3,5,2,4,6-trioxatriborinane (23a):

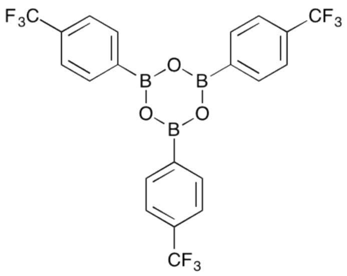

Prepared according to general procedure A using 4-(trifluoromethyl)phenylboronic acid to give the title compound as a white solid.

^{1}H NMR (600 MHz, $CDCl_3$): δ 8.35 (d, *J* = 7.9 Hz, 6 H), 7.78 (d, *J* = 7.9 Hz, 6 H). Data consistent with reported compound.[7]

***N*,*N'*,*N''*-((1,3,5,2,4,6-trioxatriborinane-2,4,6-triyl)tris(benzene-3,1-diyl))triacetamide (24a):**

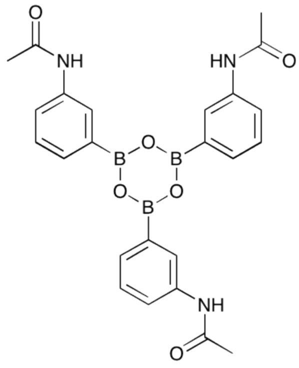

Prepared according to general procedure A using 3-acetamidophenylboronic acid to give the title compound as a white solid.

^{1}H NMR (600 MHz, $(CD_3)_2SO$): δ 9.92 (s, 3 H), 7.91 (s, 3 H), 7.80 (d, *J* = 7.9 Hz, 3 H), 7.58 (d, *J* = 7.1 Hz, 3 H), 7.32 (t, *J* = 7.7 Hz, 3 H), 2.07 (s, 9 H). ^{1}H NMR shows 6% of boronic acid.

^{13}C NMR (150 MHz, $(CD_3)_2SO$): δ 168.3, 138.7, 128.5, 127.8, 124.4, 120.7, 24.1.

^{11}B NMR (128 MHz, $(CD_3)_2SO$): δ 14.5.

FTIR (ν_{max}, cm^{-1}): 3333, 1607, 1578, 1488, 1449, 1362, 1308, 1258, 1226, 1154, 1069, 1019, 927, 811.

HRMS (ESI): calculated for $C_{24}H_{24}O_6N_3{}^{11}B_3Na$ $[M+Na]^+$ 506.1842, found 506.1833.

m.p.: >269 ˚C (Decomposition).

2,4,6-tris(4-methyl-3-nitrophenyl)-1,3,5,2,4,6-trioxatriborinane (25a):

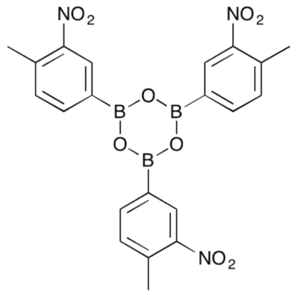

Prepared according to general procedure A using 4-methyl-3-nitrophenylboronic acid to give the title compound as a beige solid, which was insoluble in a variety of dry, deuterated NMR solvents.

FTIR (ν_{max}, cm^{-1}): 2981, 1618, 1557, 1526, 1496, 1448, 1335, 1308, 1277, 1208, 1159, 1105, 1077, 1034, 917, 903, 876.

m.p.: >262 ˚C (Decomposition).

2,4,6-tris(4-vinylphenyl)-1,3,5,2,4,6-trioxatriborinane (26a):

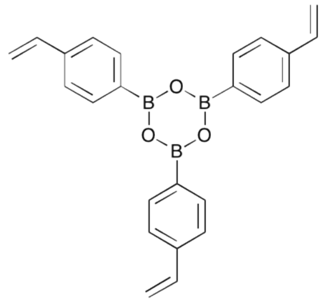

Prepared according to general procedure A using 4-vinylphenylboronic acid to give the title compound as a white solid.

^{1}H NMR (600 MHz, $CDCl_3$): δ 8.19 (d, *J* = 8.0 Hz, 6 H), 7.54 (d, *J* = 8.0 Hz, 6 H), 6.81 (dd, *J* = 17.6, 10.9 Hz, 3 H), 5.91 (d, *J* = 17.6 Hz, 3 H), 5.38 (d, *J* = 10.9 Hz, 3 H). Data consistent with reported compound.[2]

2,4,6-tri(thiophen-2-yl)-1,3,5,2,4,6-trioxatriborinane (27a):

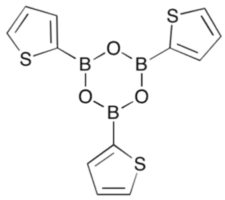

Prepared according to general procedure A using 2-thienylboronic acid to give the title compound as a beige solid.

^{1}H NMR (600 MHz, $CDCl_3$): δ 8.04 (dd, *J* = 3.5, 0.7 Hz, 3 H), 7.81 (dd, *J* = 4.6, 0.7 Hz, 3 H), 7.31 (dd, *J* = 4.6, 3.5 Hz, 3 H). Data consistent with reported compound.[8]

2,4,6-tris(3,5-di-*tert*-butylphenyl)-1,3,5,2,4,6-trioxatriborinane (28a):

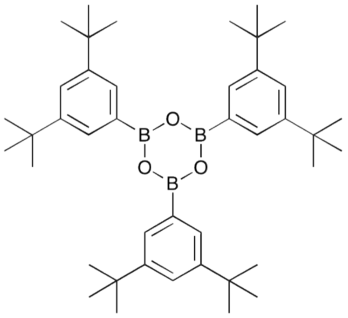

Prepared according to general procedure A using (3,5-di-*tert*-butylphenyl)boronic acid to give the title compound as a white solid.

^{1}H NMR (600 MHz, CDCl$_3$): δ 8.24 (d, *J* = 1.9 Hz, 6 H), 7.79 (m, 3 H), 1.53 (s, 54 H).

^{13}C NMR (150 MHz, CDCl$_3$): δ 150.3, 129.6, 127.1, 35.0, 31.6.

^{11}B NMR (128 MHz, CDCl$_3$): δ 28.2.

FTIR (ν_{max}, cm^{-1}): 3207, 2963, 1594, 1428, 1363, 1342, 1308, 1272, 1247, 907, 897, 890.

HRMS (ESI): calculated for $C_{42}H_{64}O_3{}^{11}B_3$ [M+H]$^+$ 649.5135, found 649.5114.

m.p.: 196-198 ˚C.

trimethyl 4,4',4''-(1,3,5,2,4,6-trioxatriborinane-2,4,6-triyl)tribenzoate (29a):

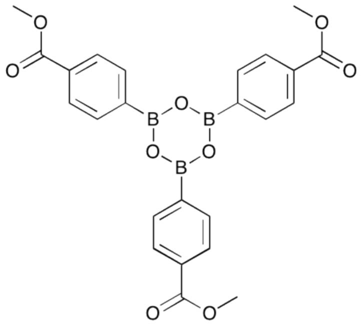

Prepared according to general procedure A using 4-methoxycarbonylphenylboronic acid to give the title compound as a white solid.

^{1}H NMR (600 MHz, CDCl$_3$): δ 8.24 (d, *J* = 8.1 Hz, 6 H), 8.13 (d, *J* = 8.1 Hz, 6 H), 3.95 (s, 9 H).

^{13}C NMR (150 MHz, CDCl$_3$): δ 167.0, 135.7, 134.0, 129.1, 52.5.

^{11}B NMR (128 MHz, CDCl$_3$): δ 28.0.

FTIR (ν_{max}, cm^{-1}): 3442, 3323, 2954, 1722, 1612, 1563, 1508, 1439, 1400, 1341, 1321, 1306, 1266, 1193, 1168, 1111, 1102, 1018, 964, 922, 859.

HRMS (ESI): calculated for $C_{24}H_{22}O_9{}^{11}B_3$ $[M+H]^+$ 487.1543, found 487.1559.

m.p.: 240-242 ˚C.

4,4',4''-(1,3,5,2,4,6-trioxatriborinane-2,4,6-triyl)tribenzonitrile (30a):

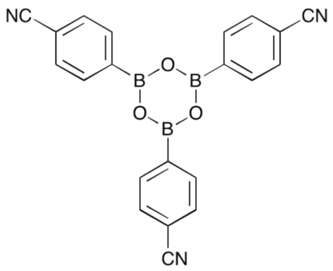

Prepared according to general procedure A using 4-cyanophenylboronic acid to give the title compound as a white solid.

^{1}H NMR (600 MHz, CDCl$_3$): δ 8.31 (d, *J* = 7.7 Hz, 6 H), 7.82 (d, *J* = 7.7 Hz, 6 H). Data consistent with reported compound.[5]

2,4,6-tris((*E*)-4-fluorostyryl)-1,3,5,2,4,6-trioxatriborinane (31a):

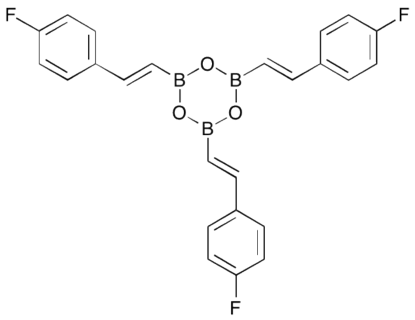

Prepared according to general procedure A using *trans*-2-(4-fluorophenyl)vinylboronic acid to give the title compound as a beige solid.

^{1}H NMR (600 MHz, CDCl$_3$): δ 7.72 (d, *J* = 18.1 Hz, 3 H), 7.59 (dd, *J* = 8.6, 5.4 Hz, 6 H), 7.09 (t, *J* = 8.6 Hz, 6 H), 6.24 (d, *J* = 18.1 Hz, 3 H). ^{1}H NMR shows 7% boronic acid.

^{13}C NMR (150 MHz, CDCl$_3$): δ 163.7 (d, *J* = 248.3 Hz), 151.1, 133.5 (d, *J* = 3.2 Hz), 129.4 (d, *J* = 8.3 Hz), 115.9 (d, *J* = 21.6 Hz).

^{11}B NMR (128 MHz, CDCl$_3$): δ 28.8.

FTIR (ν_{max}, cm^{-1}): 3228, 1620, 1600, 1508, 1414, 1352, 1271, 1220, 1182, 1157, 1095, 998, 861, 812.

HRMS (ESI): calculated for $C_{24}H_{19}O_3F_3{}^{11}B_3$ $[M]^+$ 445.1565, found 445.1580.

m.p.: 190-192 ˚C.

2,4,6-tris((*E*)-4-methylstyryl)-1,3,5,2,4,6-trioxatriborinane (32a):

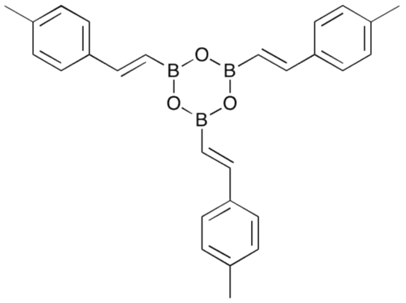

Prepared according to general procedure A using *trans*-2-(4-methylphenyl)vinylboronic acid to give the title compound as a white solid.

^{1}H NMR (600 MHz, CDCl$_3$): δ 7.75 (d, *J* = 18.1 Hz, 3 H), 7.52 (d, *J* = 8.0 Hz, 6 H), 7.21 (d, *J* = 8.0 Hz, 6 H), 6.29 (d, *J* = 18.1 Hz, 3 H), 2.39 (s, 9 H). Data consistent with reported compound.[9]

2,4,6-tris((*E*)-3-phenylprop-1-en-1-yl)-1,3,5,2,4,6-trioxatriborinane (33a):

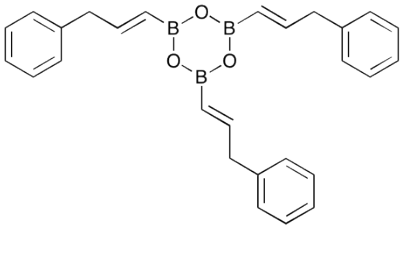

Prepared according to general procedure A using *trans*-3-phenyl-1-propen-1-ylboronic acid to give the title compound as a brown gum.

^{1}H NMR (600 MHz, CDCl$_3$): δ 7.32-7.30 (m, 6 H), 7.24-7.21 (m, 3 H), 7.18 (d, *J* = 7.3 Hz, 6 H), 7.06 (dt, *J* = 17.6, 6.2 Hz, 3 H), 5.53 (d, *J* = 17.6 Hz, 3 H), 3.53 (d, *J* = 6.2 Hz, 6 H).

^{13}C NMR (150 MHz, CDCl$_3$): δ 155.7, 139.0, 129.0, 128.7, 126.4, 42.2.

^{11}B NMR (128 MHz, $CDCl_3$): δ 27.5

FTIR (ν_{max}, cm^{-1}): 3209, 1631, 1603, 1452, 1423, 1352, 1301, 1272, 1217, 1195, 1076, 1030, 998, 935, 884.

HRMS (ESI): calculated for $C_{27}H_{28}O_3{}^{11}B_3$ [M+H$^+$] 433.2312, found 433.2308.

2,4,6-tri((*E*)-pent-1-en-1-yl)-1,3,5,2,4,6-trioxatriborinane (34a):

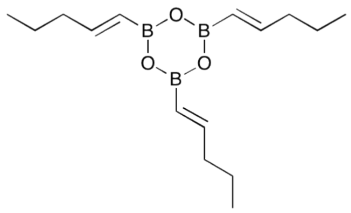

Prepared according to general procedure A using 1-penten-1-ylboronic acid to give the title compound as a grey liquid.

^{1}H NMR (400 MHz, $CDCl_3$): δ 6.96 (dt, *J* = 17.7, 6.5 Hz, 3 H), 5.54 (dt, *J* = 17.7, 1.5 Hz, 3 H), 2.23-2.17 (m, 6 H), 1.54-1.44 (m, 6 H), 0.93 (t, *J* = 7.4 Hz, 9 H).

^{13}C NMR (150 MHz, $CDCl_3$): δ 157.7, 37.9, 21.5, 13.9.

^{11}B NMR (128 MHz, $CDCl_3$): δ 28.8.

FTIR (ν_{max}, cm^{-1}): 2960, 2932, 1633, 1347, 1334, 1302, 1233, 1193, 1106, 1044, 996, 909, 876.

HRMS (ESI): calculated for $C_{15}H_{28}O_3{}^{11}B_3$ $[M+H]^+$ 289.2318, found 289.2313.

3. Synthetic Procedures and Characterisation of TMS-Bpin

General Procedure B for Preparation of TMS-Bpin: The reaction was carried out in dry conditions under an atmosphere of argon. To a mixture of boroxine (0.15 mmol, 1.0 equiv.) and *N*,*N*-diisopropylethylamine (0.094 mL, 0.54 mmol, 3.6 equiv.) in toluene (0.75 mL) was added (trimethylsilyl)diazomethane (0.23 mL, 0.465 mmol, 2 M in hexanes, 3.1 equiv.). The reaction mixture was stirred at 85 ˚C for 1 h and allowed to cool to room temperature. Pinacol (70.9 mg, 0.60 mmol, 4.0 equiv.) was added and the reaction mixture was stirred at room temperature for 2 h. The reaction was quenched with a saturated aqueous solution of NH_4Cl and the aqueous phase was extracted with EtOAc. The combined organic extracts were washed with brine, dried ($MgSO_4$) and concentrated *in vacuo*. The crude residue was purified by silica gel flash column chromatography to afford the desired TMS-Bpin product.

General Procedure C for Preparation of TMS-Bpin: The reaction was carried out in dry conditions under an atmosphere of argon. To a mixture of boroxine (0.15 mmol, 1.0 equiv.) in toluene (0.75 mL) was added (trimethylsilyl)diazomethane (0.23 mL, 0.465 mmol, 2 M in hexanes, 3.1 equiv.). The reaction mixture was stirred at 85 ˚C for 1 h and allowed to cool to room temperature. Pinacol (70.9 mg, 0.60 mmol, 4.0 equiv.) was added and the reaction mixture was stirred at room temperature for 2 h. The reaction was quenched with a saturated aqueous solution of NH_4Cl and the aqueous phase was extracted with EtOAc. The combined organic extracts were washed with brine, dried ($MgSO_4$) and concentrated *in vacuo*. The crude residue was purified by silica gel flash column chromatography to afford the desired TMS-Bpin product.

((4-methoxyphenyl)(4,4,5,5-tetramethyl-1,3,2-dioxaborolan-2-yl)methyl)trimethylsilane (5):

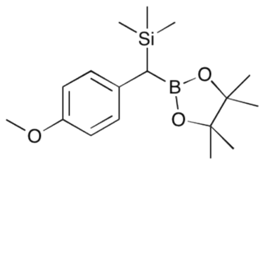

Prepared according to General Procedure B using the corresponding boroxine **4** (60.3 mg, 0.15 mmol, 1.0 equiv.). Purification by silica gel flash column chromatography (eluent: EtOAc:hexane = 1:40) afforded the title compound as a white crystalline solid (134.1 mg, 0.419 mmol, 93%).

^{1}H NMR (600 MHz, $CDCl_3$): δ 7.09 (d, *J* = 8.7 Hz, 2 H), 6.77 (d, *J* = 8.7 Hz, 2 H), 3.77 (s, 3 H), 1.90 (s, 1 H), 1.26 (s, 6 H), 1.24 (s, 6 H), -0.01 (s, 9 H).

^{13}C NMR (150 MHz, $CDCl_3$): δ 156.2, 132.7, 129.4, 113.4, 83.1, 55.3, 25.2, 25.0, -1.4.

^{11}B NMR (128 MHz, $CDCl_3$): δ 33.5.

FTIR (ν_{max}, cm^{-1}): 2978, 1610, 1579, 1508, 1465, 1371, 1342, 1306, 1290, 1244, 1141, 1112, 1037, 970, 851, 839.

HRMS (ESI): calculated for $C_{17}H_{29}O_3{}^{11}BNaSi$ $[M+Na]^+$ 343.1871, found 343.1858.

m.p.: 74-75 ˚C.

***R*$_f$** = 0.41 (EtOAc:hexane = 1:10). All data consistent with reported compound.[10]

trimethyl((3-nitrophenyl)(4,4,5,5-tetramethyl-1,3,2-dioxaborolan-2-yl)methyl)silane (15):

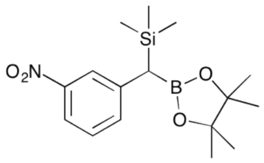

Prepared according to General Procedure B using the corresponding boroxine **15a** (67.0 mg, 0.15 mmol, 1.0 equiv.). Purification by silica gel flash column chromatography (eluent: EtOAc:hexane = 1:30) afforded the title compound as a white crystalline solid (146.3 mg, 0.436 mmol, 97%).

^{1}H NMR (600 MHz, $CDCl_3$): δ 8.03 (t, *J* = 1.9 Hz, 1 H), 7.89 (dd, *J* = 8.2, 1.4 Hz, 1 H), 7.48 (d, *J* = 7.7 Hz, 1 H), 7.34 (dd, *J* = 8.0, 7.9 Hz, 1 H), 2.11 (s, 1 H), 1.27 (s, 6 H), 1.23 (s, 6 H), 0.02 (s, 9 H).

^{13}C NMR (150 MHz, $CDCl_3$): δ 148.3, 143.7, 134.9, 128.6, 123.1, 118.8, 83.6, 25.2, 24.9, -1.5.

^{11}B NMR (128 MHz, $CDCl_3$): δ 32.7.

FTIR (ν_{max}, cm^{-1}): 2981, 1524, 1479, 1392, 1371, 1347, 1312, 1274, 1245, 1166, 1139, 1037, 967, 933, 912, 839.

HRMS (ESI): calculated for $C_{16}H_{27}O_4{}^{11}BNSi$ $[M+H]^+$ 336.1802, found 336.1804.

m.p.: 105-107 ˚C.

***R*$_f$** = 0.34 (EtOAc:hexane = 1:10).

trimethyl((4,4,5,5-tetramethyl-1,3,2-dioxaborolan-2-yl)(2-(trifluoromethoxy)phenyl)methyl)silane (16):

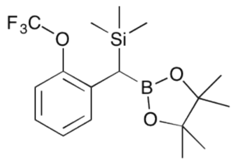

Prepared according to General Procedure B using the corresponding boroxine **16a** (84.6 mg, 0.15 mmol, 1.0 equiv.). Purification by silica gel flash column chromatography (eluent: EtOAc:hexane = 1:40) afforded the title compound as a colourless oil (141.5 mg, 0.378 mmol, 84%).

^{1}H NMR (600 MHz, CDCl$_3$): δ 7.49 (dd, *J* = 7.8, 1.5 Hz, 1 H), 7.18-7.14 (m, 2 H, H10), 7.07-7.04 (m, 1 H), 2.47 (s, 1 H), 1.26 (s, 6 H), 1.23 (s, 6 H), 0.02 (s, 9 H).

^{13}C NMR (150 MHz, CDCl$_3$): δ 146.5, 134.2, 130.8, 126.1, 124.6, 120.9 (q, *J* = 255.1 Hz), 120.0 (d, *J* = 1.2 Hz), 83.4, 25.1, 25.0, -1.2.

^{11}B NMR (128 MHz, CDCl$_3$): δ 33.7.

FTIR (ν_{max}, cm^{-1}): 2980, 1605, 1489, 1453, 1373, 1345, 1319, 1292, 1247, 1225, 1196, 1141, 1082, 1038, 1004, 970, 924, 837.

HRMS (ESI): calculated for $C_{17}H_{27}O_3{}^{11}BF_3Si$ $[M+H]^+$ 375.1775, found 375.1780.

***R*$_f$** = 0.34 (EtOAc:hexane = 1:40).

((4-bromophenyl)(4,4,5,5-tetramethyl-1,3,2-dioxaborolan-2-yl)methyl)trimethylsilane (17):

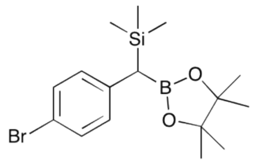

Prepared according to General Procedure B using the corresponding boroxine **17a** (82.3 mg, 0.15 mmol, 1.0 equiv.). Purification by silica gel flash column chromatography (eluent: EtOAc:hexane = 1:40) afforded the title compound as a white crystalline solid (124.7 mg, 0.338 mmol, 75%).

^{1}H NMR (600 MHz, CDCl$_3$): δ 7.30 (d, *J* = 8.5 Hz, 2 H), 7.05 (d, *J* = 8.5 Hz, 2 H), 1.94 (s, 1 H), 1.26 (s, 6 H), 1.23 (s, 6 H), 0.00 (s, 9 H).

^{13}C NMR (150 MHz, CDCl$_3$): δ 140.1, 130.9, 130.4, 117.1, 83.3, 25.2, 25.0, -1.5.

^{11}B NMR (128 MHz, CDCl$_3$): δ 32.5.

FTIR (ν_{max}, cm^{-1}): 2979, 1483, 1404, 1391, 1367, 1338, 1316, 1298, 1263, 1248, 1209, 1165, 1140, 1071, 1050, 1007, 968, 853, 838.

HRMS (ESI): calculated for $C_{16}H_{27}O_2{}^{11}BBrSi$ [M+H]$^+$ 369.1057, found 369.1062.

m.p.: 82-84 ˚C.

***R*$_f$** = 0.14 (EtOAc:hexane = 1:40).

((4-chlorophenyl)(4,4,5,5-tetramethyl-1,3,2-dioxaborolan-2-yl)methyl)trimethylsilane (18):

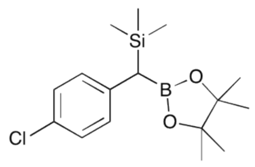

Prepared according to General Procedure B using the corresponding boroxine **18a** (62.3 mg, 0.15 mmol, 1.0 equiv.). Purification by silica gel flash column chromatography (eluent: EtOAc:hexane = 1:40) afforded the title compound as a white crystalline solid (115.5 mg, 0.356 mmol, 79%).

^{1}H NMR (600 MHz, CDCl$_3$): δ 7.16 (d, *J* = 8.6 Hz, 2 H), 7.09 (d, *J* = 8.6 Hz, 2 H), 1.95 (s, 1 H), 1.26 (s, 6 H), 1.23 (s, 6 H), 0.00 (s, 9 H).

^{13}C NMR (150 MHz, CDCl$_3$): δ 139.6, 130.0, 129.2, 128.0, 83.3, 25.3, 25.0, -1.4.

^{11}B NMR (128 MHz, CDCl$_3$): δ 33.2.

FTIR (ν_{max}, cm^{-1}): 2980, 1487, 1409, 1392, 1370, 1338, 1315, 1300, 1265, 1249, 1210, 1167, 1141, 1088, 1050, 1012, 968, 841.

HRMS (ESI): calculated for $C_{16}H_{27}O_2{}^{11}BClSi$ [M+H]$^+$ 325.1562, found 325.1561.

m.p.: 83-84 ˚C.

$\boldsymbol{R_f}$ = 0.26 (EtOAc:hexane = 1:20). All data consistent with reported compound.[10]

trimethyl(phenyl(4,4,5,5-tetramethyl-1,3,2-dioxaborolan-2-yl)methyl)silane (19):

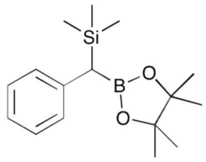

Prepared according to General Procedure B using the corresponding boroxine **19a** (46.8 mg, 0.15 mmol, 1.0 equiv.). Purification by silica gel flash column chromatography (eluent: EtOAc:hexane = 1:30) afforded the title compound as a white crystalline solid (87.6 mg, 0.302 mmol, 67%).

^{1}H NMR (600 MHz, CDCl$_3$): δ 7.22-7.17 (m, 4 H), 7.06-7.03 (m, 1 H), 1.98 (s, 1 H), 1.26 (s, 6 H), 1.24 (s, 6 H), 0.00 (s, 9 H).

^{13}C NMR (150 MHz, CDCl$_3$): δ 140.9, 128.8, 127.9, 123.5, 83.2, 25.3, 25.0, -1.4.

^{11}B NMR (128 MHz, CDCl$_3$): δ 33.2.

FTIR (ν_{max}, cm^{-1}): 2979, 1600, 1495, 1451, 1372, 1349, 1307, 1269, 1247, 1211, 1140, 1033, 1003, 970, 915, 837.

HRMS (ESI): calculated for $C_{16}H_{28}O_2{}^{11}BSi$ [M+H]$^+$ 291.1952, found 291.1937.

m.p.: 40-42 ˚C.

$\boldsymbol{R_f}$ = 0.16 (EtOAc:hexane = 1:30).

((4-(*tert*-butyl)phenyl)(4,4,5,5-tetramethyl-1,3,2-dioxaborolan-2-yl)methyl)trimethylsilane (20):

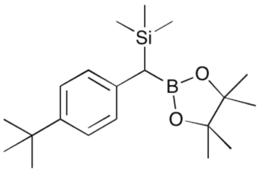

Prepared according to General Procedure B using the corresponding boroxine **20a** (72.0 mg, 0.15 mmol, 1.0 equiv.). Purification by silica gel flash column chromatography (eluent:

EtOAc:hexane = 1:40) afforded the title compound as a white amorphous solid (110.7 mg, 0.320 mmol, 71%).

^{1}H NMR (600 MHz, CDCl$_3$): δ 7.20 (d, *J* = 8.4 Hz, 2 H), 7.09 (d, *J* = 8.4 Hz, 2 H), 1.94 (s, 1 H), 1.30 (s, 9 H), 1.26 (s, 6 H), 1.25 (s, 6 H), 0.00 (s, 9 H).

^{13}C NMR (150 MHz, CDCl$_3$): δ 146.0, 137.5, 128.3, 124.8, 83.1, 34.3, 31.6, 25.3, 25.2, -1.3.

^{11}B NMR (128 MHz, CDCl$_3$): δ 33.6.

FTIR (ν_{max}, cm^{-1}): 2963, 1611, 1513, 1466, 1417, 1364, 1339, 1308, 1268, 1247, 1213, 1142, 1117, 1084, 1023, 969, 841.

HRMS (ESI): calculated for $C_{20}H_{36}O_2{}^{11}BSi$ $[M+H]^+$ 347.2578, found 347.2585.

m.p.: 105-107 ˚C.

***R*$_f$** = 0.16 (EtOAc:hexane = 1:40).

((4-fluorophenyl)(4,4,5,5-tetramethyl-1,3,2-dioxaborolan-2-yl)methyl)trimethylsilane (21):

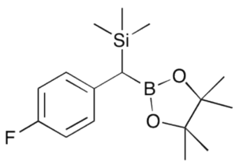

Prepared according to General Procedure B using the corresponding boroxine **21a** (54.9 mg, 0.15 mmol, 1.0 equiv.). Purification by silica gel flash column chromatography (eluent: EtOAc:hexane = 1:50) afforded the title compound as a colourless oil (98.6 mg, 0.320 mmol, 71%).

^{1}H NMR (600 MHz, CDCl$_3$): δ 7.12-7.10 (m, 2 H), 6.91-6.88 (m, 2 H), 1.95 (s, 1 H), 1.26 (s, 6 H), 1.24 (s, 6 H), 0.00 (s, 9 H).

^{13}C NMR (150 MHz, CDCl$_3$): δ 160.1 (d, *J* = 239.2 Hz), 136.4 (d, *J* = 3.0 Hz), 129.7 (d, *J* = 7.3 Hz), 114.6 (d, *J* = 20.7 Hz), 83.3, 25.2, 25.0, -1.5.

^{11}B NMR (128 MHz, CDCl$_3$): δ 32.8.

FTIR (ν_{max}, cm^{-1}): 2979, 1603, 1506, 1469, 1372, 1341, 1248, 1220, 1140, 1032, 970, 840.

HRMS (ESI): calculated for $C_{16}H_{27}O_2{}^{11}BFSi$ $[M+H]^+$ 309.1857, found 309.1857.

***R*$_f$** = 0.48 (EtOAc:hexane = 1:10). All data consistent with reported compound.[10]

trimethyl((4,4,5,5-tetramethyl-1,3,2-dioxaborolan-2-yl)(*o*-tolyl)methyl)silane (22):

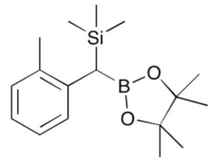

Prepared according to General Procedure B using the corresponding boroxine **22a** (53.1 mg, 0.15 mmol, 1.0 equiv.). Purification by silica gel flash column chromatography (eluent: EtOAc:hexane = 1:30) afforded the title compound as a white crystalline solid (113.7 mg, 0.374 mmol, 83%).

^{1}H NMR (600 MHz, CDCl$_3$): δ 7.39 (d, *J* = 7.7 Hz, 1 H), 7.12-7.09 (m, 2 H), 6.96 (m, 1 H), 2.22 (s, 4 H), 1.27 (s, 6 H), 1.24 (s, 6 H), 0.02 (s, 9 H).

^{13}C NMR (150 MHz, CDCl$_3$): δ 139.5, 134.7, 130.0, 128.7, 125.5, 123.5, 83.2, 25.3, 25.0, 21.2, -1.1.

^{11}B NMR (128 MHz, CDCl$_3$): δ 33.5.

FTIR (ν_{max}, cm^{-1}): 2978, 1602, 1485, 1370, 1338, 1315, 1290, 1269, 1247, 1213, 1143, 1091, 1030, 970, 850, 839.

HRMS (ESI): calculated for $C_{17}H_{30}O_2{}^{11}BSi$ $[M+H]^+$ 305.2108, found 305.2114.

m.p.: 41-43 ˚C.

***R*$_f$** = 0.45 (EtOAc:hexane = 1:10). All data consistent with reported compound.[10]

trimethyl((4,4,5,5-tetramethyl-1,3,2-dioxaborolan-2-yl)(4-(trifluoromethyl)phenyl)methyl)silane (23):

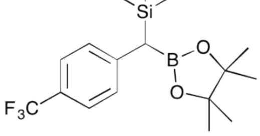

Prepared according to General Procedure B using the corresponding boroxine **23a** (77.4 mg, 0.15 mmol, 1.0 equiv.). Purification by silica gel flash column chromatography (eluent: DCM:

hexane = 1:5) afforded the title compound as a white amorphous solid (119.4 mg, 0.333 mmol, 74%).

^{1}H NMR (600 MHz, CDCl$_3$): δ 7.45 (d, *J* = 8.1 Hz, 2 H), 7.27 (d, *J* = 8.1 Hz, 2 H), 2.08 (s, 1 H), 1.28 (s, 6 H), 1.25 (s, 6 H), 0.02 (s, 9 H).

^{13}C NMR (150 MHz, CDCl$_3$): δ 145.8 (d, *J* = 1.3 Hz), 128.8, 125.9 (q, *J* = 32.0 Hz), 124.91 (q, *J* = 269.7 Hz), 124.87 (q, *J* = 3.8 Hz), 83.5, 25.2, 25.0, -1.4.

^{11}B NMR (128 MHz, CDCl$_3$): δ 32.8.

FTIR (ν_{max}, cm^{-1}): 2981, 1615, 1514, 1392, 1373, 1321, 1268, 1249, 1213, 1141, 1115, 1067, 1018, 969, 908, 841.

HRMS (ESI): calculated for $C_{17}H_{27}O_2{}^{11}BF_3Si$ [M+H]$^+$ 359.1825, found 359.1839.

m.p.: 73-75 ˚C.

***R*$_f$** = 0.27 (EtOAc:hexane = 1:50).

***N*-(3-((4,4,5,5-tetramethyl-1,3,2-dioxaborolan-2-yl)(trimethylsilyl)methyl)phenyl)acetamide (24):**

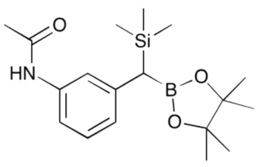

Prepared according to General Procedure B using the corresponding boroxine **24a** (72.4 mg, 0.15 mmol, 1.0 equiv.). Purification by silica gel flash column chromatography (eluent: EtOAc:hexane = 1:3, then MeOH:CHCl$_3$ = 1:50) afforded the title compound as a pale yellow, waxy solid (98.4 mg, 0.283 mmol, 63%).

^{1}H NMR (600 MHz, CDCl$_3$): δ 7.51 (s, 1 H), 7.37 (dd, *J* = 8.0, 1.1 Hz, 1 H,), 7.14-7.10 (m, 2 H), 6.91 (d, *J* = 7.7 Hz, 1 H), 2.13 (s, 3 H), 1.94 (s, 1 H), 1.24 (s, 6 H), 1.22 (s, 6 H), -0.01 (s, 9 H).

^{13}C NMR (150 MHz, CDCl$_3$): δ 168.5, 141.9, 137.6, 128.5, 124.9, 119.8, 115.5, 83.2, 25.2, 25.0, 24.7, -1.4.

^{11}B NMR (128 MHz, CDCl$_3$): δ 33.3.

FTIR (ν_{max}, cm^{-1}): 3301, 2979, 1664, 1608, 1553, 1488, 1436, 1371, 1339, 1307, 1247, 1214, 1140, 1022, 969, 909, 839.

HRMS (ESI): calculated for $C_{18}H_{30}O_3{}^{11}BNSi$ $[M]^+$ 347.2088, found 347.2104.

m.p.: 135-137 ˚C.

***R*$_f$** = 0.24 (EtOAc:hexane = 1:3).

trimethyl((4-methyl-3-nitrophenyl)(4,4,5,5-tetramethyl-1,3,2-dioxaborolan-2-yl)methyl)silane (25):

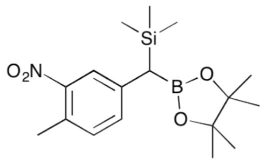

Prepared according to General Procedure B using the corresponding boroxine **25a** (73.3 mg, 0.15 mmol, 1.0 equiv.). Purification by silica gel flash column chromatography (eluent: EtOAc:hexane = 1:20) afforded the title compound as a white crystalline solid (124.1 mg, 0.355 mmol, 79%).

^{1}H NMR (600 MHz, CDCl$_3$): δ 7.77 (d, *J* = 1.8 Hz, 1 H), 7.29 (dd, *J* = 8.0, 1.8 Hz, 1 H), 7.14 (d, *J* = 8.0 Hz, 1 H), 2.51 (s, 3 H), 2.02 (s, 1 H) 1.26 (s, 6 H), 1.23 (s, 6 H), 0.02 (s, 9 H).

^{13}C NMR (150 MHz, CDCl$_3$): δ 149.1, 140.8, 133.5, 132.1, 128.4, 124.2, 83.6, 25.2, 24.9, 20.0, -1.5.

^{11}B NMR (128 MHz, CDCl$_3$): δ 33.0.

FTIR (ν_{max}, cm^{-1}): 2979, 1526, 1449, 1410, 1372, 1343, 1293, 1270, 1250, 1214, 1141, 1036, 971, 849.

HRMS (ESI): calculated for $C_{17}H_{29}O_4{}^{11}BNSi$ $[M+H]^+$ 350.1959, found 350.1971.

m.p.: 88-90 ˚C.

***R*$_f$** = 0.19 (EtOAc:hexane = 1:20).

trimethyl((4,4,5,5-tetramethyl-1,3,2-dioxaborolan-2-yl)(4-vinylphenyl)methyl)silane (26):

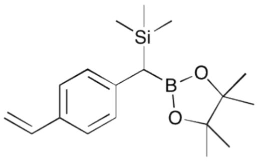

Prepared according to General Procedure B using the corresponding boroxine **26a** (58.5 mg, 0.15 mmol, 1.0 equiv.). Purification by silica gel flash column chromatography (eluent: EtOAc:hexane = 1:20) afforded the title compound as a white crystalline solid (129.6 mg, 0.410 mmol, 91%).

^{1}H NMR (600 MHz, CDCl$_3$): δ 7.26 (d, *J* = 8.2 Hz, 2 H), 7.13 (d, *J* = 8.2 Hz, 2 H), 6.67 (dd, *J* = 17.6, 10.9 Hz), 5.66 (dd, *J* = 17.6, 0.8 Hz, 1 H), 5.12 (dd, *J* = 10.9, 0.8 Hz, 1 H) 1.98 (s, 1 H), 1.26 (s, 6 H), 1.24 (s, 6 H), 0.01 (s, 9 H).

^{13}C NMR (150 MHz, CDCl$_3$): δ 141.0, 137.1, 133.0, 128.9, 125.9, 111.8, 83.2, 25.3, 25.1, -1.3.

^{11}B NMR (128 MHz, CDCl$_3$): δ 33.7.

FTIR (ν_{max}, cm^{-1}): 2979, 1629, 1608, 1508, 1426, 1343, 1310, 1248, 1214, 1141, 1033, 970, 853, 843.

HRMS (ESI): calculated for $C_{18}H_{30}O_2{}^{11}BSi$ [M+H]$^+$ 317.2108, found 317.2111.

m.p.: 46-48 ˚C.

***R*$_f$** = 0.22 (EtOAc:hexane = 1:20).

trimethyl((4,4,5,5-tetramethyl-1,3,2-dioxaborolan-2-yl)(thiophen-2-yl)methyl)silane (27):

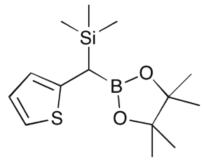

Prepared according to General Procedure B using the corresponding boroxine **27a** (49.5 mg, 0.15 mmol, 1.0 equiv.). Purification by silica gel flash column chromatography (eluent: EtOAc:hexane = 1:50) afforded the title compound as a colourless oil (116.1 mg, 0.392 mmol, 87%).

^{1}H NMR (600 MHz, CDCl$_3$): δ 6.94 (d, *J* = 5.2 Hz, 1 H), 6.88 (dd, *J* = 5.2, 3.2 Hz, 1 H), 6.73 (d, *J* = 3.2 Hz, 1 H), 2.28 (s, 1 H), 1.27 (s, 6 H), 1.25 (s, 6 H), 0.06 (s, 9 H).

^{13}C NMR (150 MHz, CDCl$_3$): δ 143.0, 126.8, 123.1, 120.6, 83.5, 25.3, 25.0, -1.5.

^{11}B NMR (128 MHz, CDCl$_3$): δ 32.7.

FTIR (ν_{max}, cm^{-1}): 2979, 1522, 1436, 1371, 1316, 1247, 1204, 1166, 1142, 1113, 1068, 1032, 968, 840.

HRMS (ESI): calculated for $C_{14}H_{26}O_2{}^{11}BSSi$ $[M+H]^+$ 297.1516, found 297.1520.

***R*$_f$** = 0.38 (EtOAc:hexane = 1:20).

((3,5-di-*tert*-butylphenyl)(4,4,5,5-tetramethyl-1,3,2-dioxaborolan-2-yl)methyl)trimethylsilane (28):

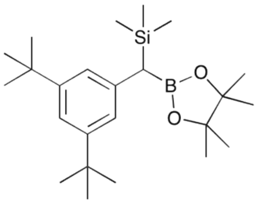

Prepared according to General Procedure B using the corresponding boroxine **28a** (97.3 mg, 0.15 mmol, 1.0 equiv.). Purification by silica gel flash column chromatography (eluent: DCM:hexane = 1:6) afforded the title compound as a white crystalline solid (143.1 mg, 0.356 mmol, 79%).

^{1}H NMR (600 MHz, CDCl$_3$): δ 7.05 (t, *J* = 1.8 Hz, 1 H), 7.03 (d, *J* = 1.8 Hz, 2 H), 1.93 (s, 1 H), 1.30 (s, 18 H), 1.27 (s, 6 H), 1.25 (s, 6 H), -0.02 (s, 9 H).

^{13}C NMR (150 MHz, CDCl$_3$): δ 149.7, 139.2, 123.5, 117.0, 83.0, 34.8, 31.7, 25.4, 25.2, -1.4.

^{11}B NMR (128 MHz, CDCl$_3$): δ 33.7.

FTIR (ν_{max}, cm^{-1}): 2963, 1594, 1479, 1390, 1371, 1337, 1309, 1269, 1247, 1205, 1144, 1040, 973, 948, 849, 841.

HRMS (ESI): calculated for $C_{24}H_{44}{}^{11}O_2BSi$ $[M+H]^+$ 403.3204, found 403.3208.

m.p.: 110-112 ˚C.

***R*$_f$** = 0.35 (EtOAc:hexane = 1:50).

methyl 4-((4,4,5,5-tetramethyl-1,3,2-dioxaborolan-2-yl)(trimethylsilyl)methyl)benzoate (29):

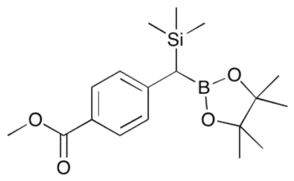

Prepared according to General Procedure C using the corresponding boroxine **29a** (72.9 mg, 0.15 mmol, 1.0 equiv.). Purification by silica gel flash column chromatography (eluent: EtOAc:hexane = 1:40) afforded the title compound as a white amorphous solid (94.1 mg, 0.270 mmol, 60%).

^{1}H NMR (600 MHz, CDCl$_3$): δ 7.87 (d, *J* = 8.4 Hz, 2 H), 7.22 (d, *J* = 8.4 Hz, 2 H), 3.87 (s, 3 H), 2.08 (s, 1 H), 1.26 (s, 6 H), 1.23 (s, 6 H), 0.00 (s, 9 H).

^{13}C NMR (150 MHz, CDCl$_3$): δ 167.7, 147.5, 129.4, 128.6, 125.6, 83.4, 51.9, 25.3, 25.0, -1.4.

^{11}B NMR (128 MHz, CDCl$_3$): δ 33.0.

FTIR (ν_{max}, cm^{-1}): 2978, 1719, 1606, 1508, 1435, 1274, 1182, 1141, 1021, 969, 866, 837.

HRMS (ESI): calculated for $C_{18}H_{30}O_4{}^{11}BSi$ [M+H]$^+$ 349.2006, found 349.2018.

m.p.: 97-99 ˚C.

***R*f** = 0.25 (EtOAc:hexane = 1:20).

4-((4,4,5,5-tetramethyl-1,3,2-dioxaborolan-2-yl)(trimethylsilyl)methyl)benzonitrile (30):

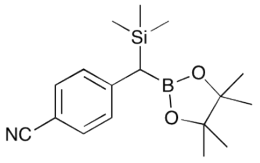

Prepared according to General Procedure C using the corresponding boroxine **30a** (58.0 mg, 0.15 mmol, 1.0 equiv.) Purification by silica gel flash column chromatography (eluent: EtOAc:hexane = 1:30 → 1:20) afforded the title compound as a white amorphous solid (102.1 mg, 0.326 mmol, 72%).

^{1}H NMR (600 MHz, CDCl$_3$): δ 7.48 (d, *J* = 8.2 Hz, 2 H), 7.26 (d, *J* = 8.2 Hz, 2 H), 2.09 (s, 1 H), 1.27 (s, 6 H), 1.24 (s, 6 H), 0.01 (s, 9 H).

^{13}C NMR (150 MHz, CDCl$_3$): δ 147.9, 131.8, 129.3, 119.9, 107.1, 83.6, 25.3, 25.0, -1.5.

^{11}B NMR (128 MHz, CDCl$_3$): δ 32.9.

FTIR (ν_{max}, cm^{-1}): 2978, 2225, 1604, 1500, 1419, 1345, 1307, 1250, 1214, 1141, 1033, 970, 852.

HRMS (ESI): calculated for $C_{17}H_{27}O_2{}^{11}BNSi$ $[M+H]^+$ 316.1904, found 316.1910.

m.p.: 114-116 ˚C.

***R*f** = 0.19 (EtOAc:hexane = 1:20).

(*E*)-(3-(4-fluorophenyl)-1-(4,4,5,5-tetramethyl-1,3,2-dioxaborolan-2-yl)allyl)trimethylsilane (31):

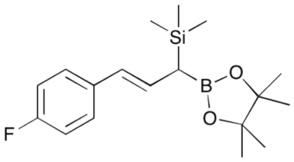

Prepared according to General Procedure B using the corresponding boroxine **31a** (66.6 mg, 0.15 mmol, 1.0 equiv.). Purification by silica gel flash column chromatography (eluent: EtOAc:hexane = 1:50) afforded the title compound as a white amorphous solid (99.3 mg, 0.297 mmol, 66%).

^{1}H NMR (600 MHz, CDCl$_3$): δ 7.28-7.26 (m, 2H), 6.96-6.93 (m, 2H), 6.26 (dd, *J* = 15.7, 10.8 Hz, 1 H), 6.12 (d, *J* = 15.7 Hz, 1H), 1.67 (d, *J* = 10.8 Hz, 1 H), 1.26 (s, 6 H), 1.25 (s, 6 H), 0.09 (s, 9 H).

^{13}C NMR (150 MHz, CDCl$_3$): δ 161.5 (d, *J* = 242.9 Hz), 135.1 (d, *J* = 3.2 Hz), 128.9 (d, *J* = 2.1 Hz), 126.9 (d, *J* = 7.6 Hz), 126.0, 115.3 (d, *J* = 21.2 Hz), 83.2, 25.14, 25.12, -1.4.

^{11}B NMR (128 MHz, CDCl$_3$): δ 33.0.

FTIR (ν_{max}, cm^{-1}): 2982, 2957, 1638, 1592, 1509, 1468, 1372, 1352, 1308, 1265, 1249, 1225, 1159, 1139, 1112, 1079, 1035, 974, 907, 860, 838.

HRMS (ESI): calculated for $C_{18}H_{29}O_2{}^{11}BFSi$ $[M+H]^+$ 335.2014, found 335.2007.

m.p.: 102-104 ˚C.

***R*f** = 0.16 (EtOAc:hexane = 1:50).

(*E*)-trimethyl(1-(4,4,5,5-tetramethyl-1,3,2-dioxaborolan-2-yl)-3-(*p*-tolyl)allyl)silane (32):

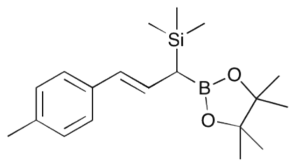

Prepared according to General Procedure B using the corresponding boroxine **32a** (64.8 mg, 0.15 mmol, 1.0 equiv.). Purification by silica gel flash column chromatography (eluent: EtOAc:hexane = 1:60) afforded the title compound as a white amorphous solid (89.2mg, 0.270 mmol, 60%).

^{1}H NMR (600 MHz, $CDCl_3$): δ 7.24 (d, *J* = 8.0 Hz, 2 H), 7.09 (d, *J* = 8.0 Hz, 2 H), 6.31 (dd, *J* = 15.7, 10.9 Hz, 1 H), 6.13 (d, *J* = 15.7 Hz, 1 H), 2.32 (s, 3 H), 1.68 (d, *J* = 10.9 Hz, 1 H), 1.265 (s, 6 H), 1.256 (s, 6 H), 0.09 (s, 9 H).

^{13}C NMR (150 MHz, $CDCl_3$): δ 136.1, 135.6, 129.2, 128.1, 126.9, 125.5, 83.1, 25.11, 25.10, 21.2, -1.4.

^{11}B NMR (128 MHz, $CDCl_3$): δ 33.5.

FTIR (ν_{max}, cm^{-1}): 2978, 1636, 1513, 1447, 1371, 1354, 1314, 1297, 1263, 1248, 1214, 1139, 1112, 1018, 970, 856, 837.

HRMS (ESI): calculated for $C_{19}H_{32}O_2{}^{11}BSi$ $[M+H]^+$ 331.2265, found 331.2267.

m.p.: 78-80 ˚C.

***R*$_f$** = 0.24 (EtOAc:hexane = 1:50).

(*E*)-trimethyl(4-phenyl-1-(4,4,5,5-tetramethyl-1,3,2-dioxaborolan-2-yl)but-2-en-1-yl)silane (33):

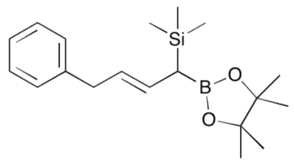

Prepared according to General Procedure B using the corresponding boroxine **33a** (64.8 mg, 0.15 mmol, 1.0 equiv.). Purification by silica gel flash column chromatography (eluent: EtOAc:hexane = 1:50) afforded the title compound as a pale yellow liquid (98.1 mg, 0.297 mmol, 66%).

^{1}H NMR (600 MHz, CDCl$_3$): δ 7.29-7.27 (m, 2 H), 7.20-7.17 (m, 3 H, H11), 5.64 (dd, *J* = 15.1, 10.7 Hz, 1 H), 5.33 (dt, *J* = 15.1, 7.0 Hz), 3.36 (d, *J* = 7.0 Hz, 2 H), 1.50 (d, *J* = 10.7 Hz), 1.26 (s, 12 H, H1), 0.06 (s, 9 H).

^{13}C NMR (150 MHz, CDCl$_3$): δ 142.0, 128.7, 128.5, 128.3, 126.1, 125.8, 83.0, 39.6, 25.1, 25.0, -1.5.

^{11}B NMR (128 MHz, CDCl$_3$): δ 33.2.

FTIR (ν_{max}, cm^{-1}): 2979, 1651, 1604, 1494, 1453, 1355, 1314, 1261, 1247, 1213, 1140, 1082, 1029, 970, 837.

HRMS (ESI): calculated for $C_{19}H_{32}O_2{}^{11}BSi$ [M+H]$^+$ 331.2265, found 331.2260.

***R*$_f$** = 0.12 (EtOAc:hexane = 1:50).

(*E*)-trimethyl(1-(4,4,5,5-tetramethyl-1,3,2-dioxaborolan-2-yl)hex-2-en-1-yl)silane (34):

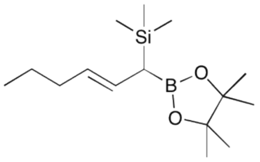

Prepared according to General Procedure B using the corresponding boroxine **34a** (43.2 mg, 0.15 mmol, 1.00 equiv.). Purification by silica gel flash column chromatography (eluent: EtOAc:hexane = 1:50) afforded the title compound as a colourless liquid (81.4 mg, 0.288 mmol, 64%).

^{1}H NMR (600 MHz, CDCl$_3$): δ 5.44 (ddt, *J* = 15.1, 10.5, 1.3 Hz, 1 H), 5.15 (dt, *J* = 15.1, 6.9 Hz), 2.00-1.90 (m, 2 H), 1.41 (d, *J* = 10.5 Hz, 1 H), 1.37-1.30 (m, 2 H), 1.230 (s, 6 H), 1.227 (s, 6 H), 0.86 (t, *J* = 7.4 Hz, 3 H), 0.03, (s, 9 H).

^{13}C NMR (150 MHz, CDCl$_3$): δ 127.8, 126.9, 82.9, 35.2, 25.11, 25.07, 23.4, 13.8, -1.5.

^{11}B NMR (128 MHz, CDCl$_3$): δ 33.2.

FTIR (ν_{max}, cm^{-1}): 2959, 1651, 1465, 1356, 1314, 1247, 1216, 1143, 1023, 970, 849, 837.

HRMS (ESI): calculated for $C_{15}H_{32}O_2{}^{11}BSi$ [M+H]$^+$ 283.2265, found 223.2260.

***R*$_f$** = 0.28 (EtOAc:hexane = 1:50).

4. ^1H and ^{13}C NMR Spectra of Boroxines

2,4,6-tris(2-(trifluoromethoxy)phenyl)-1,3,5,2,4,6-trioxatriborinane (16a):

^1H NMR, 600 MHz, $CDCl_3$:

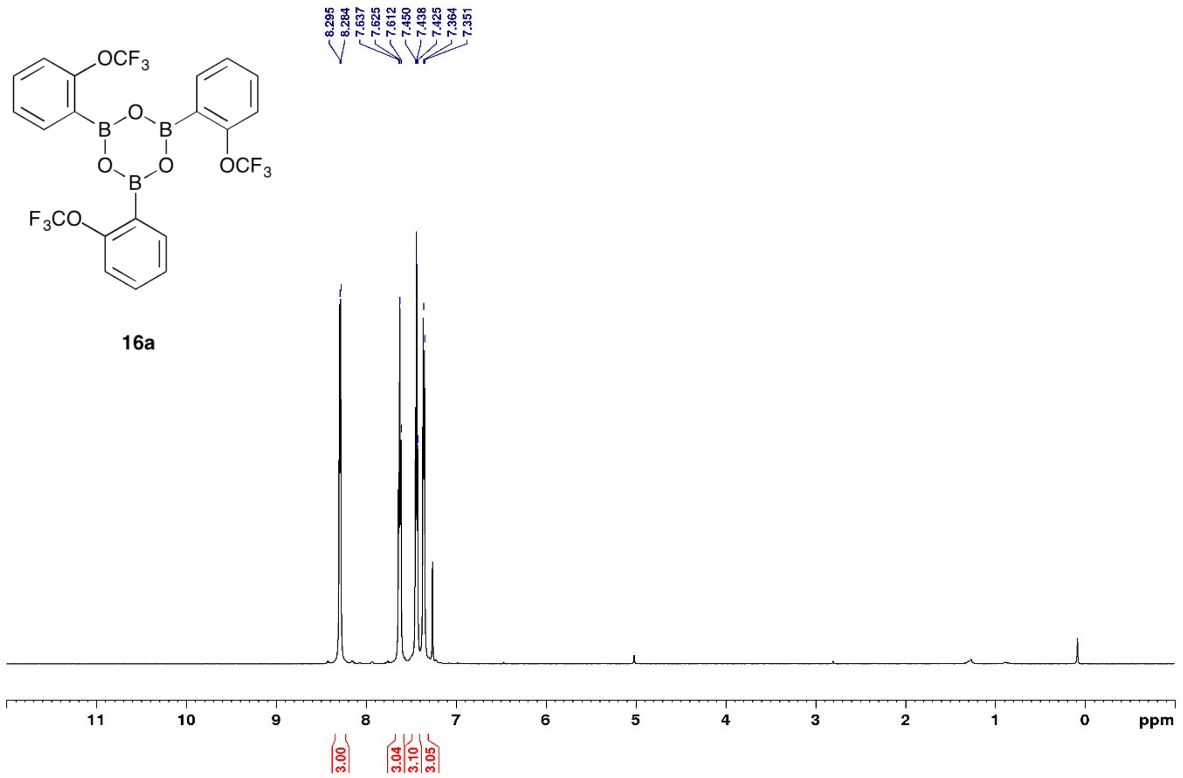

^{13}C NMR, 150 MHz, $CDCl_3$:

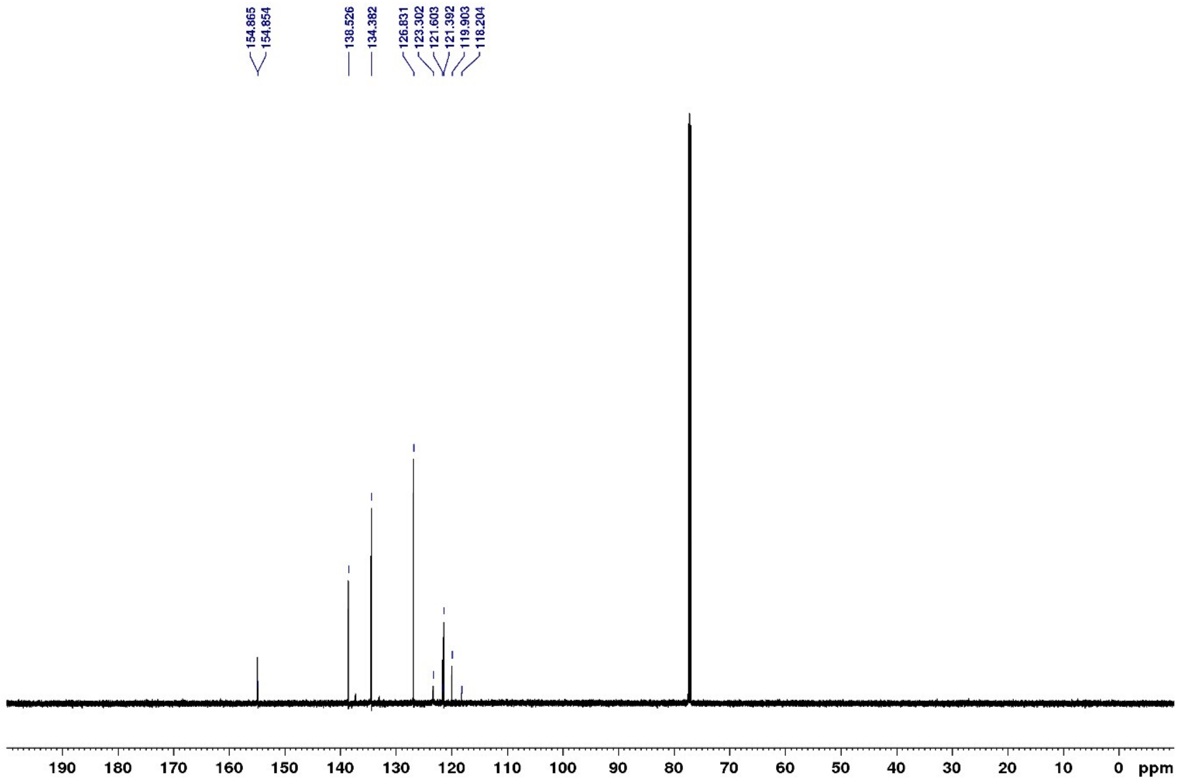
154.865
154.854
138.526
134.382
126.831
123.302
121.603
121.392
119.903
118.204
190 180 170 160 150 140 130 120 110 100 90 80 70 60 50 40 30 20 10 0 ppm

2,4,6-tris(4-bromophenyl)-1,3,5,2,4,6-trioxatriborinane (17a):

^{1}H NMR, 600 MHz, $(CD_3)_2SO$:

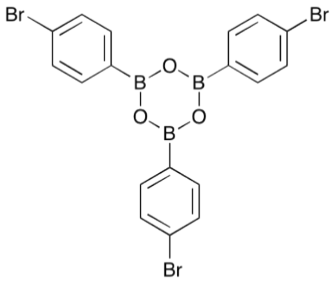

17a

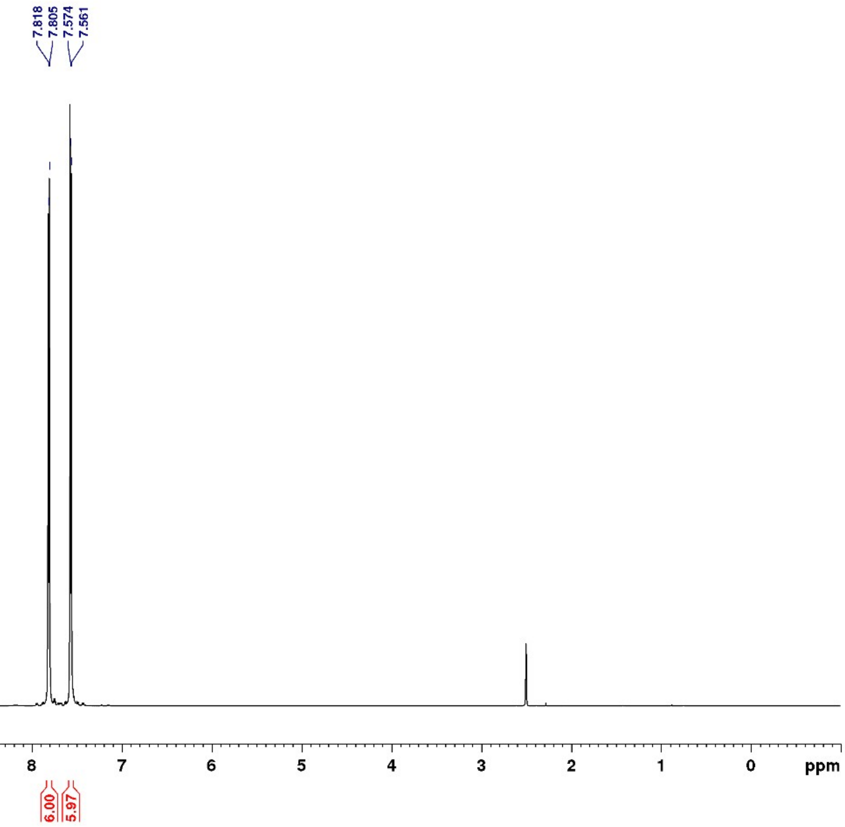

^{13}C NMR, 150 MHz, $(CD_3)_2SO$:

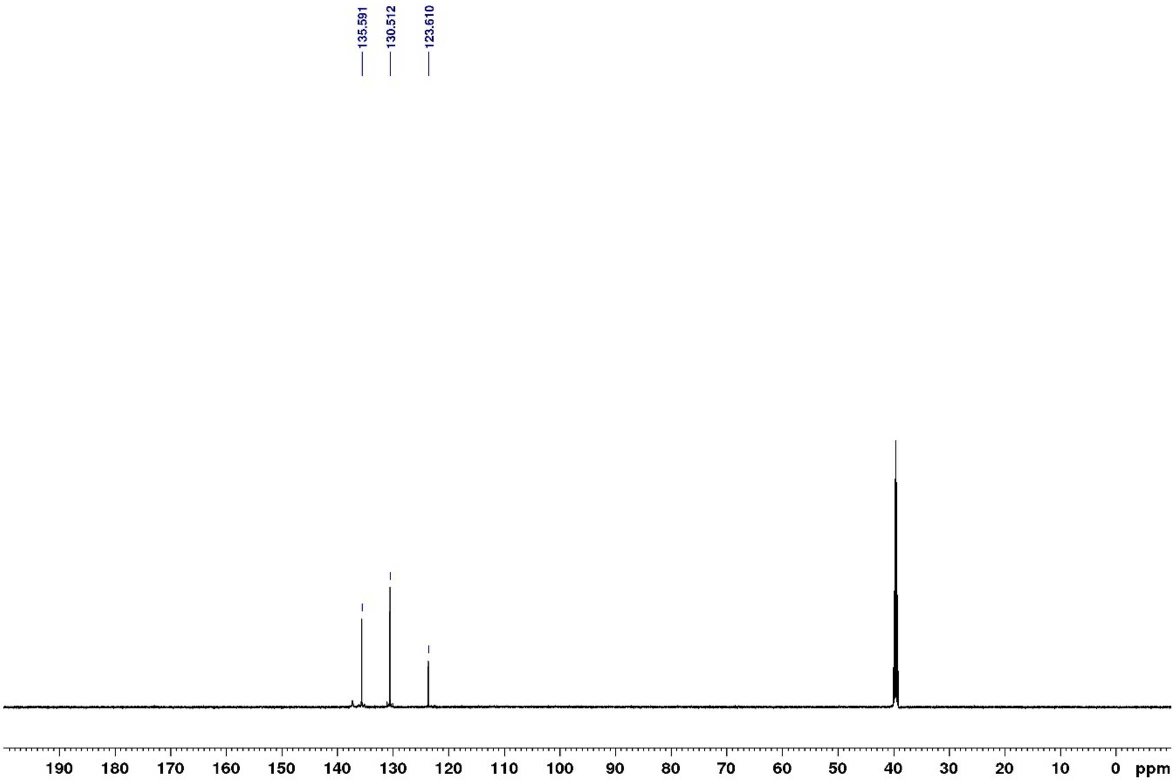

***N*,*N*',*N*''-((1,3,5,2,4,6-trioxatriborinane-2,4,6-triyl)tris(benzene-3,1-diyl))triacetamide (24a):**

^{1}H NMR, 600 MHz, $(CD_3)_2SO$:

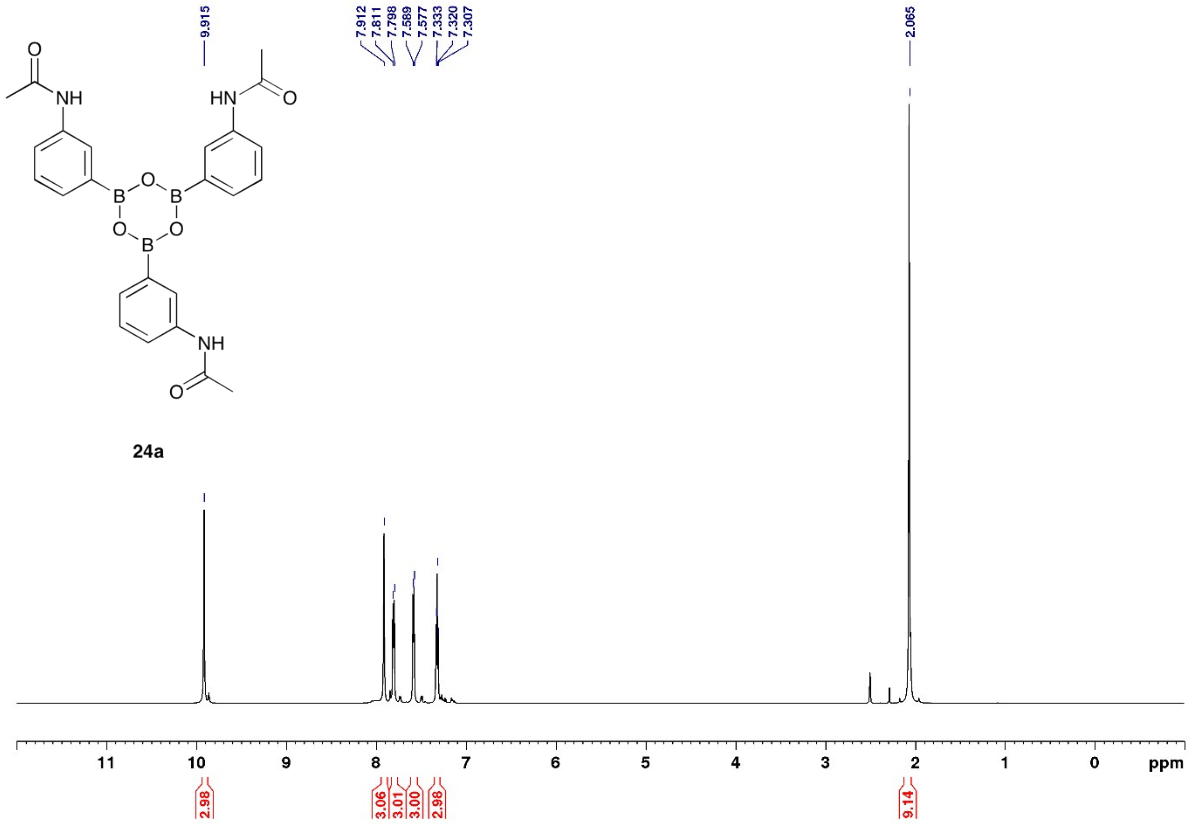

^{13}C NMR, 150 MHz, $(CD_3)_2SO$:

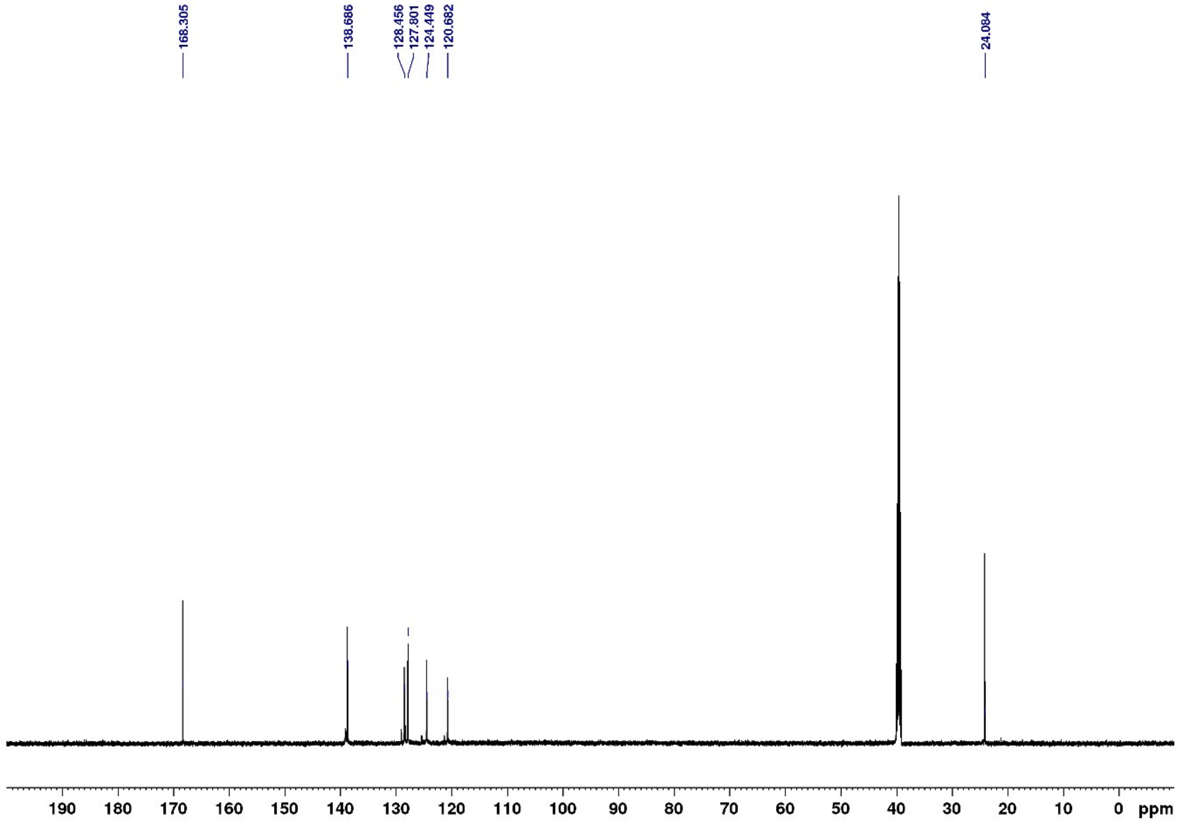

2,4,6-tris(3,5-di-*tert*-butylphenyl)-1,3,5,2,4,6-trioxatriborinane (28a):

^{1}H NMR, 600 MHz, $CDCl_3$:

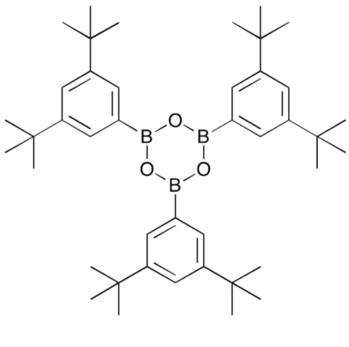

28a

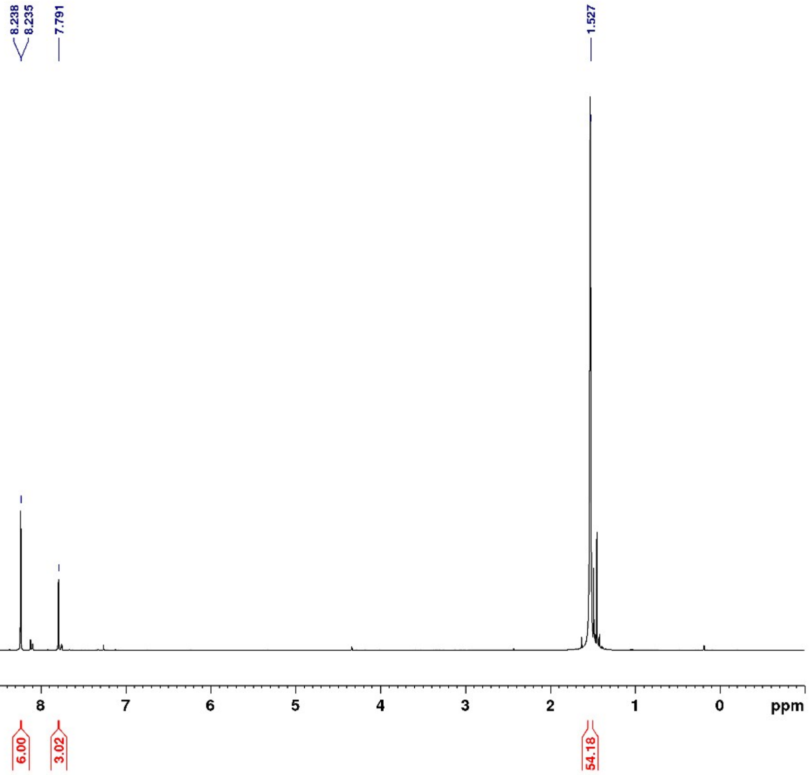

^{13}C NMR, 150 MHz, $CDCl_3$:

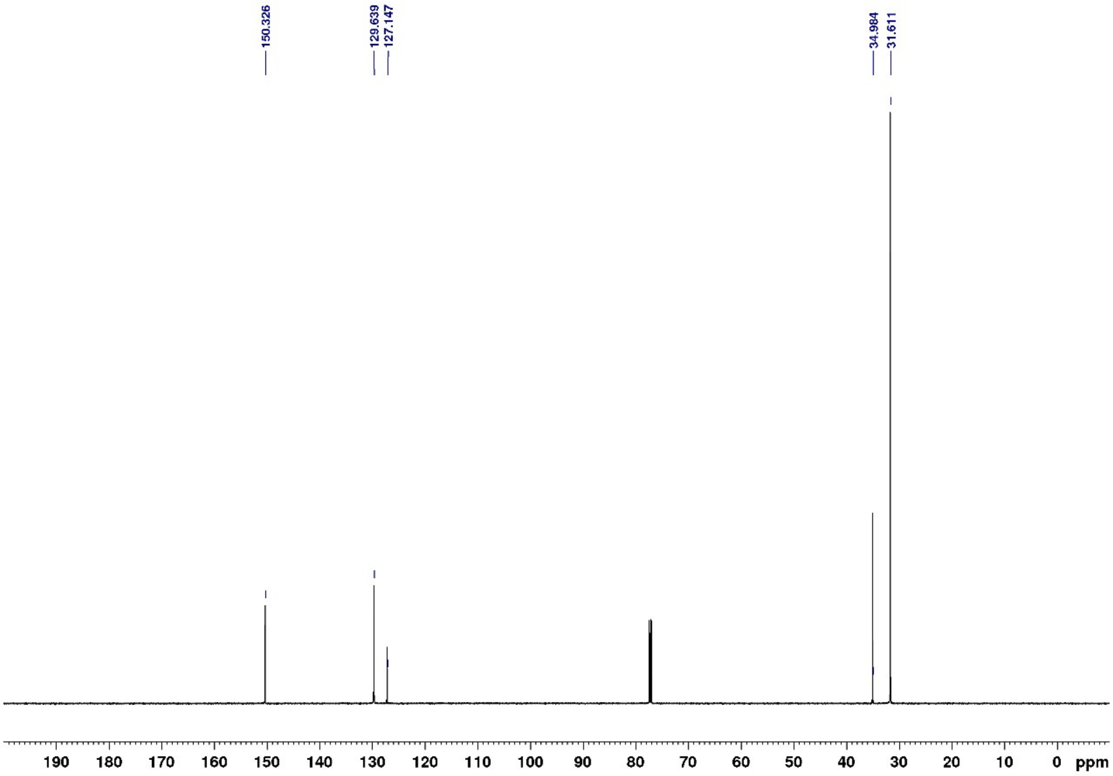

trimethyl 4,4',4''-(1,3,5,2,4,6-trioxatriborinane-2,4,6-triyl)tribenzoate (29a):

^{1}H NMR, 600 MHz, CDCl$_3$:

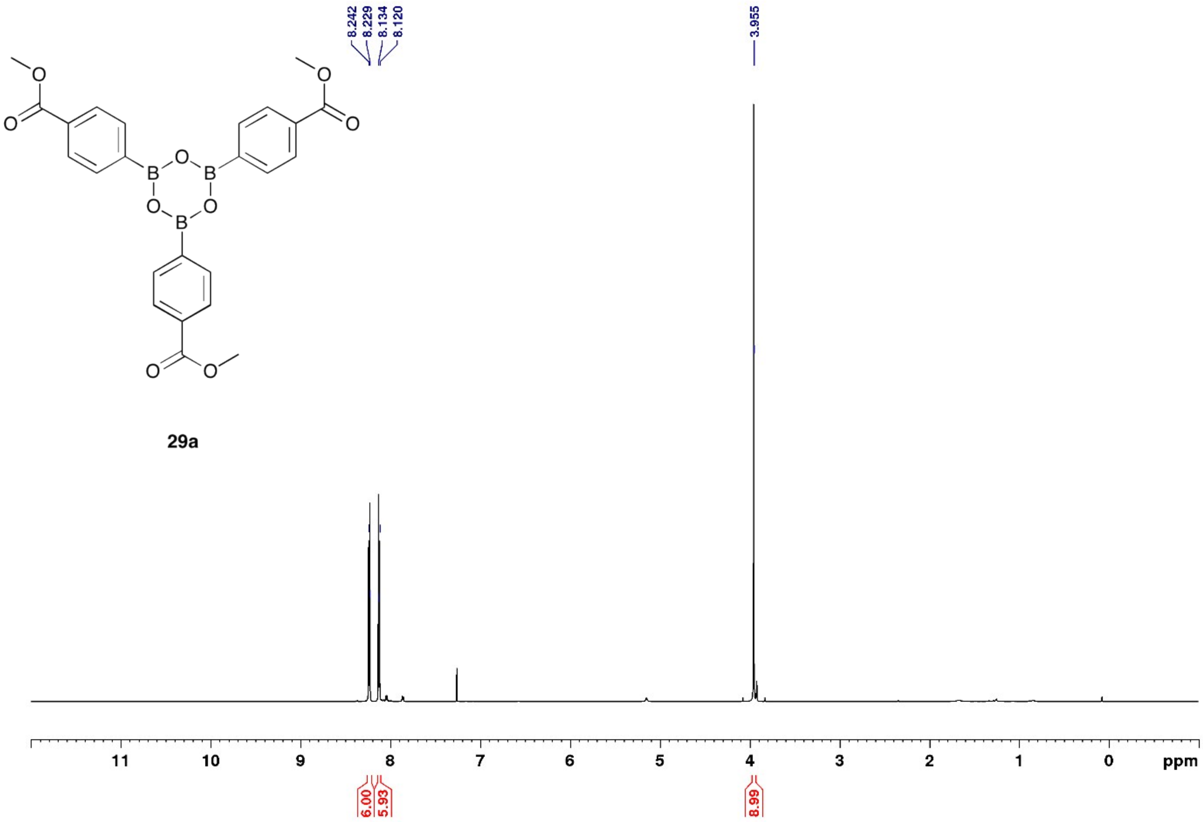

^{13}C NMR, 150 MHz, CDCl$_3$:

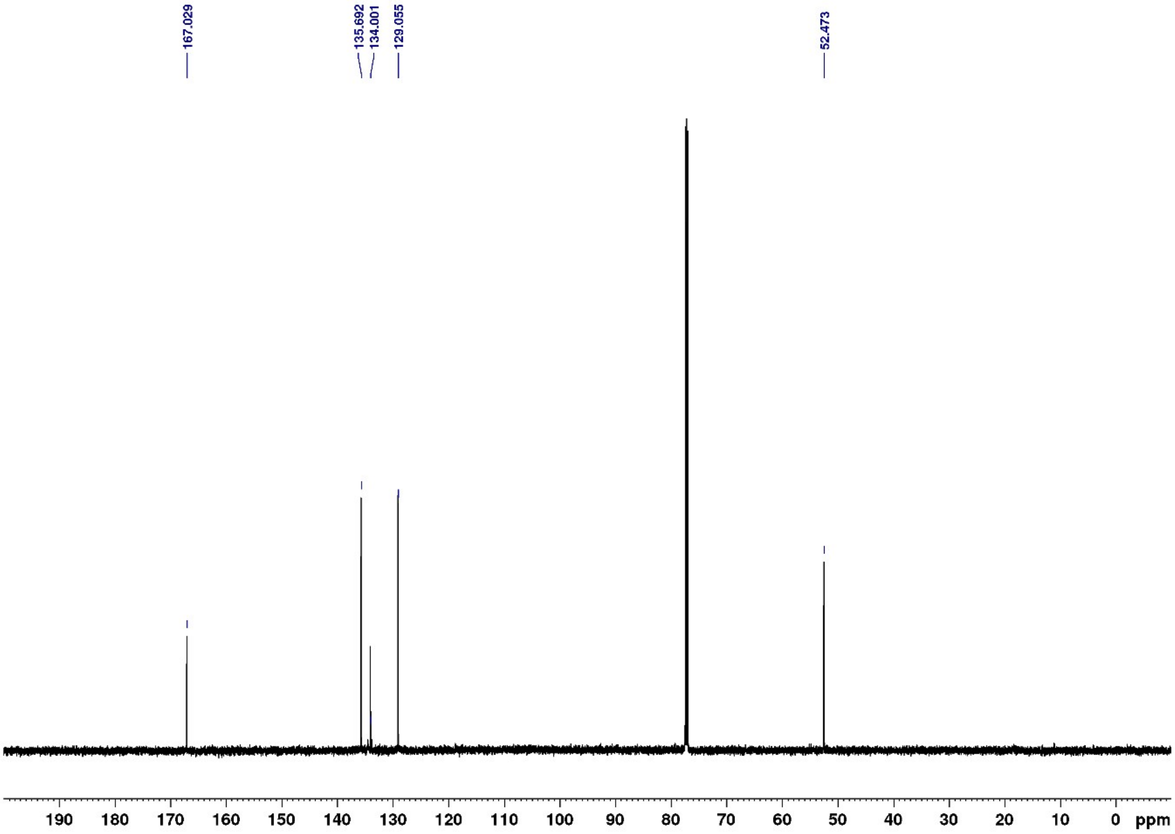

2,4,6-tris((*E*)-4-fluorostyryl)-1,3,5,2,4,6-trioxatriborinane (31a):

^{1}H NMR, 600 MHz, $CDCl_3$:

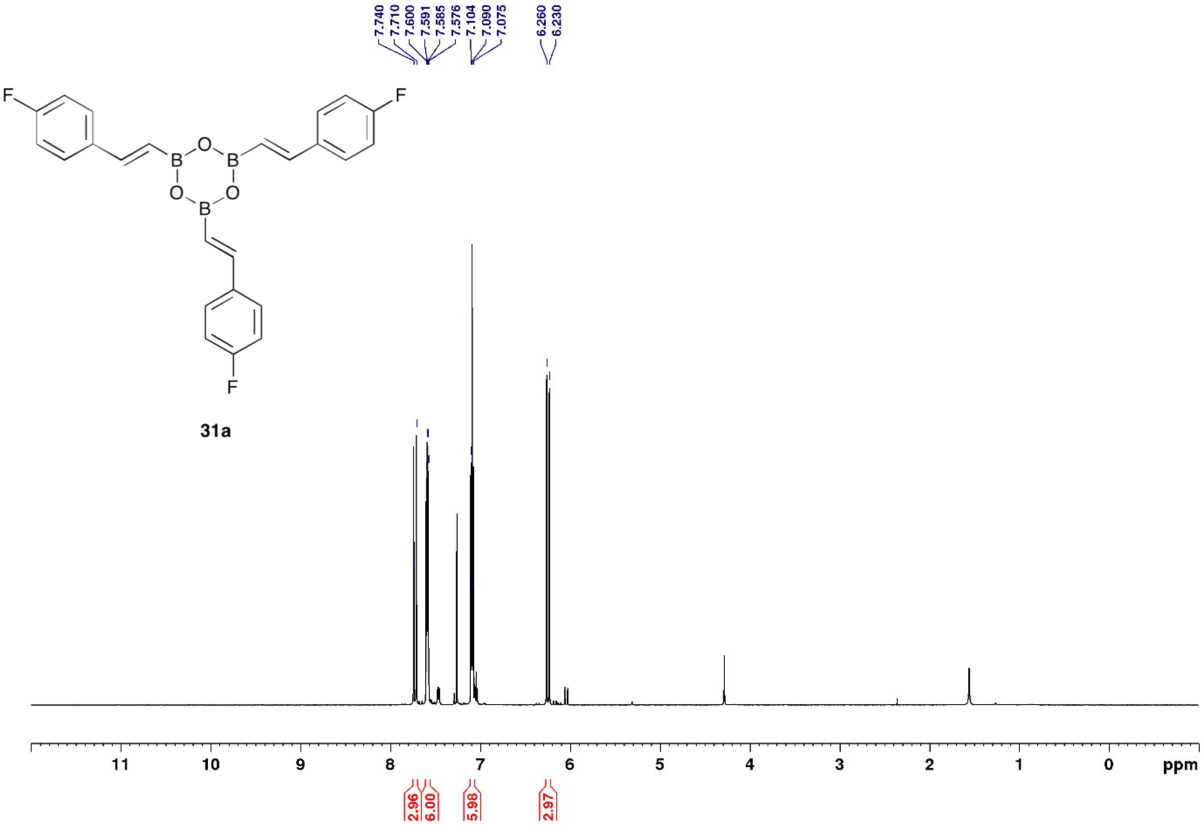

^{13}C NMR, 150 MHz, $CDCl_3$:

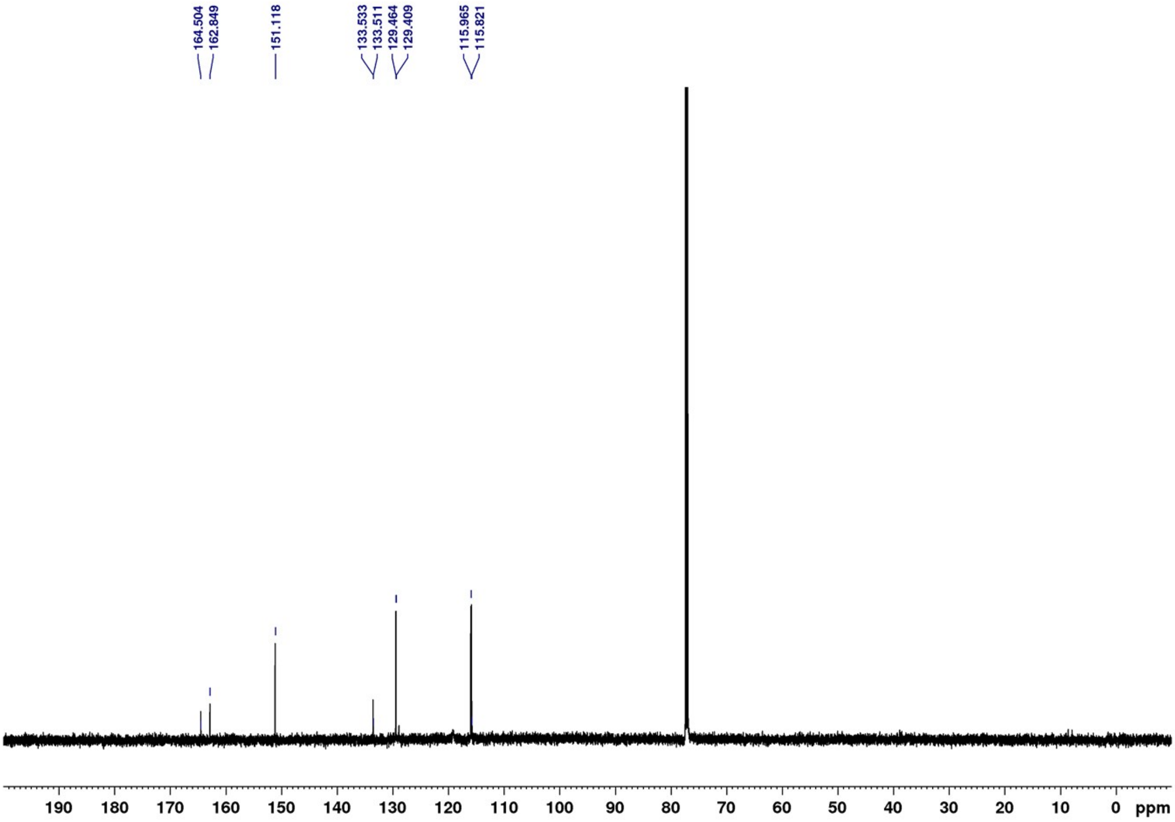

2,4,6-tris((*E*)-3-phenylprop-1-en-1-yl)-1,3,5,2,4,6-trioxatriborinane (33a):

^{1}H NMR, 600 MHz, $CDCl_3$:

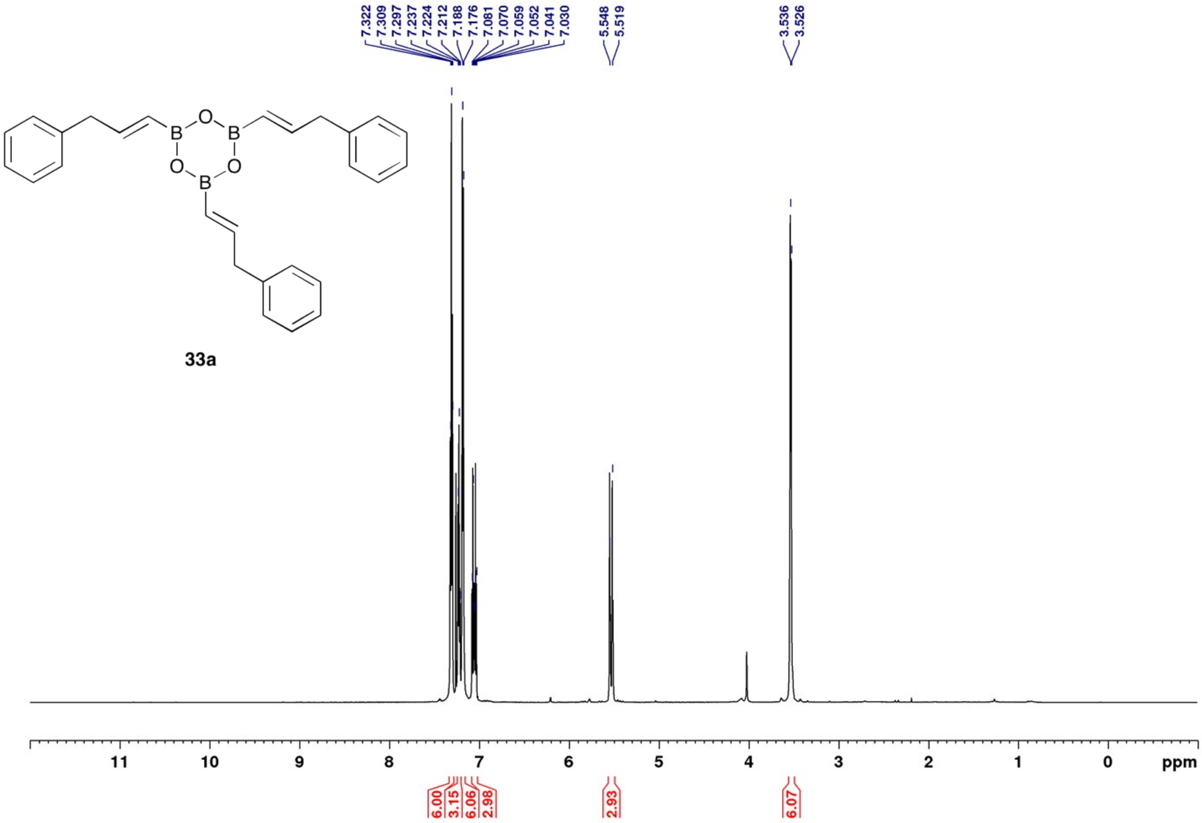

^{13}C NMR, 150 MHz, $CDCl_3$:

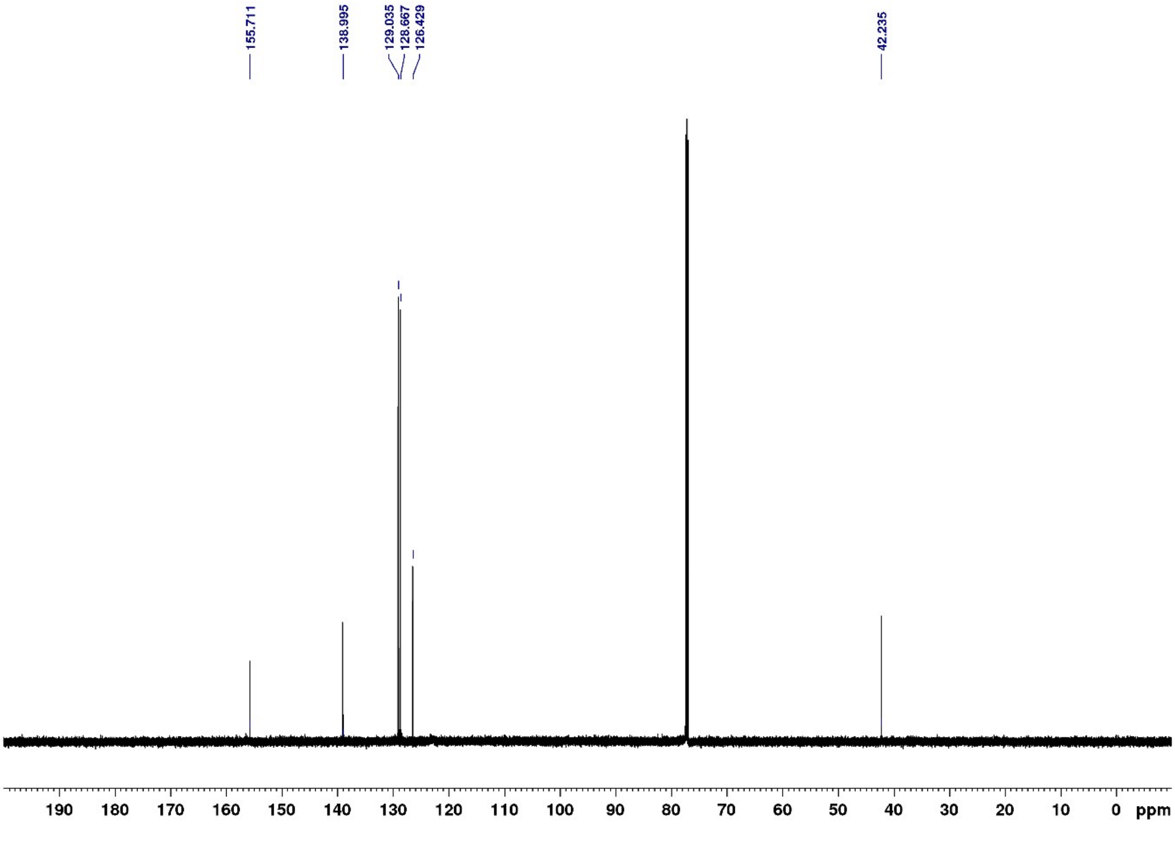

2,4,6-tri((*E*)-pent-1-en-1-yl)-1,3,5,2,4,6-trioxatriborinane (34a):

^{1}H NMR, 400 MHz, $CDCl_3$:

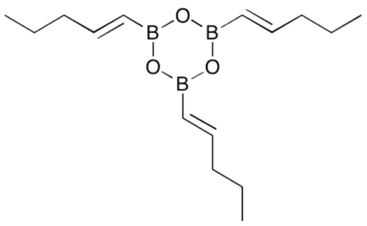

34a

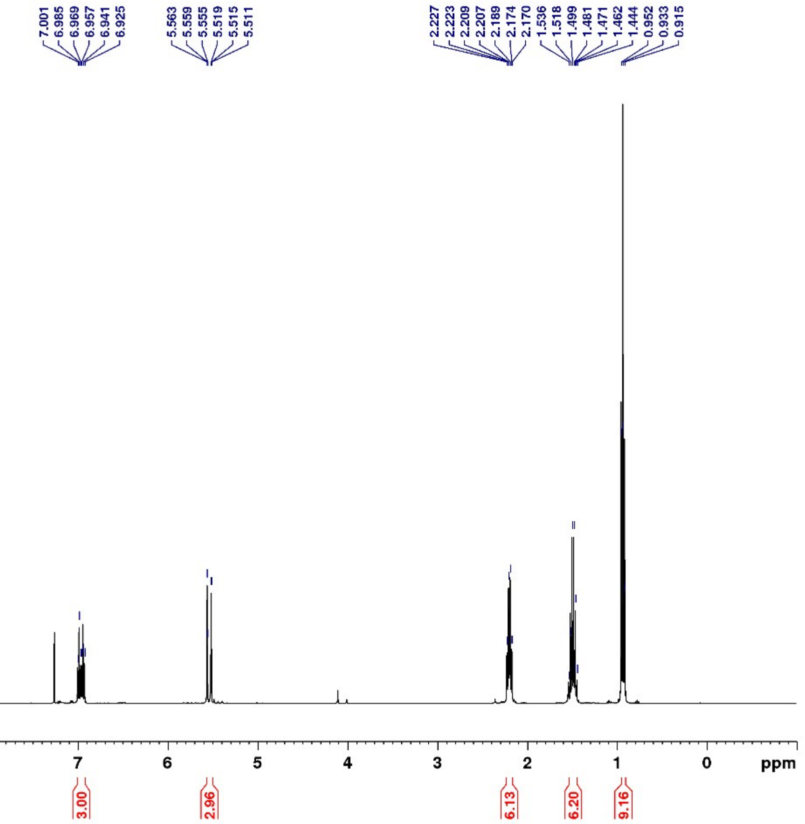

^{13}C NMR, 150 MHz, $CDCl_3$:

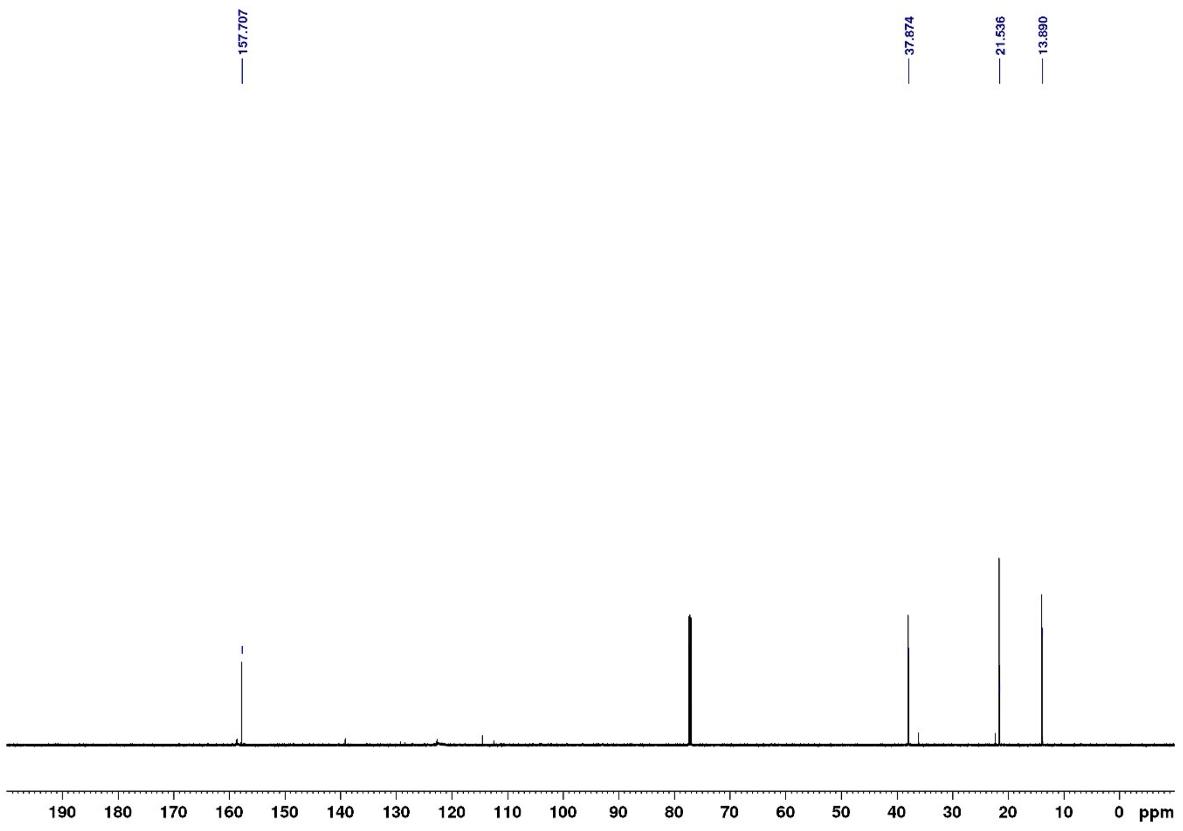

157.707
37.874
21.536
13.890
190 180 170 160 150 140 130 120 110 100 90 80 70 60 50 40 30 20 10 0 ppm

5. ^{1}H and ^{13}C NMR Spectra of TMS-Bpin

((4-methoxyphenyl)(4,4,5,5-tetramethyl-1,3,2-dioxaborolan-2-yl)methyl)trimethylsilane (5):

^{1}H NMR, 600 MHz, CDCl$_3$:

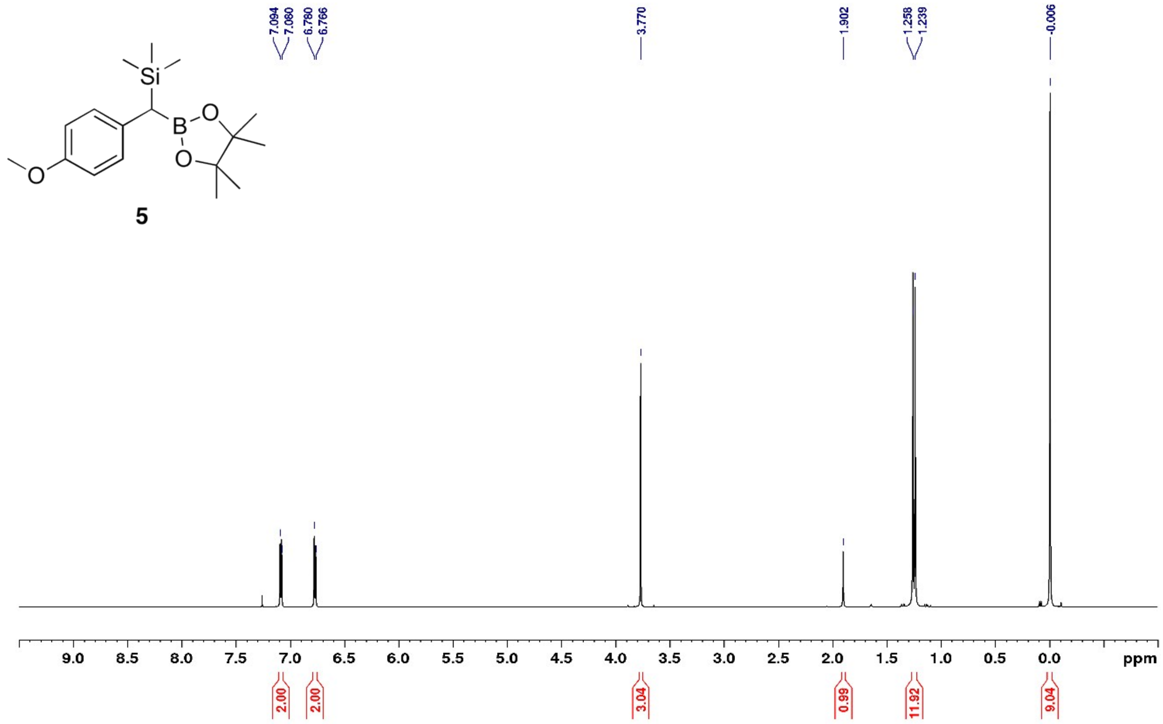

^{13}C NMR, 150 MHz, CDCl$_3$:

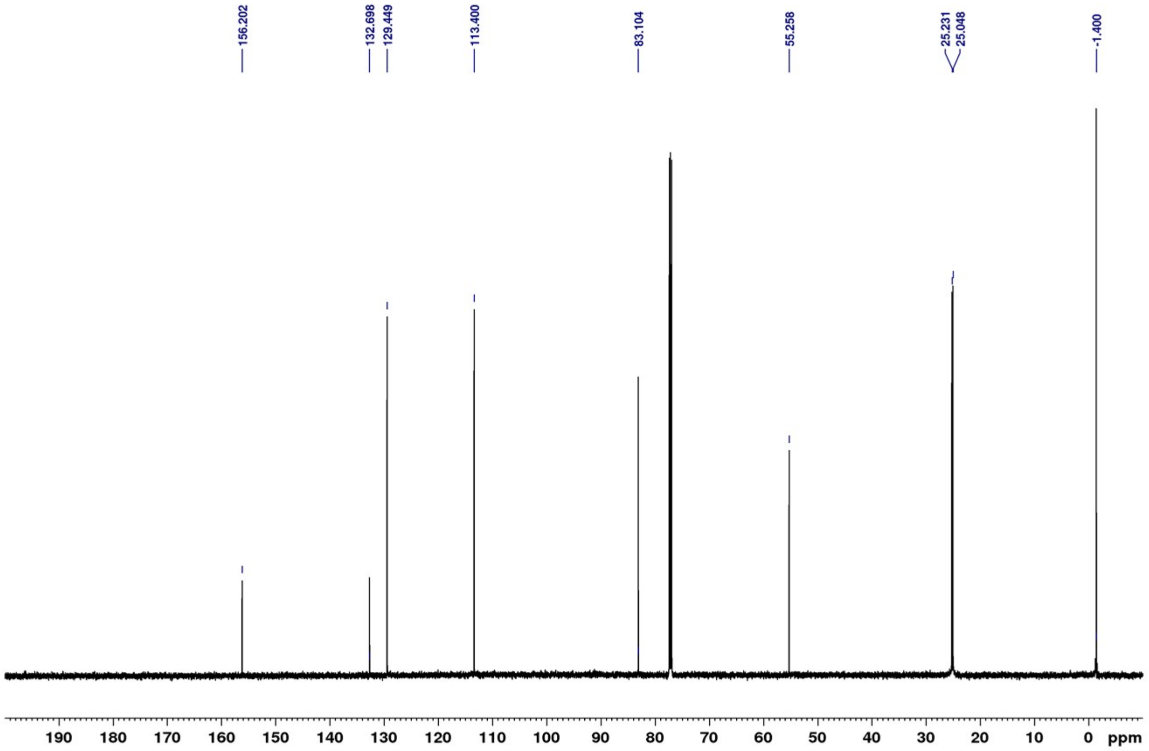

trimethyl((3-nitrophenyl)(4,4,5,5-tetramethyl-1,3,2-dioxaborolan-2-yl)methyl)silane (15):

^{1}H NMR, 600 MHz, CDCl$_3$:

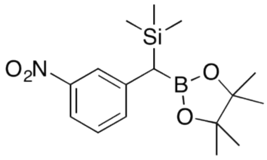

15

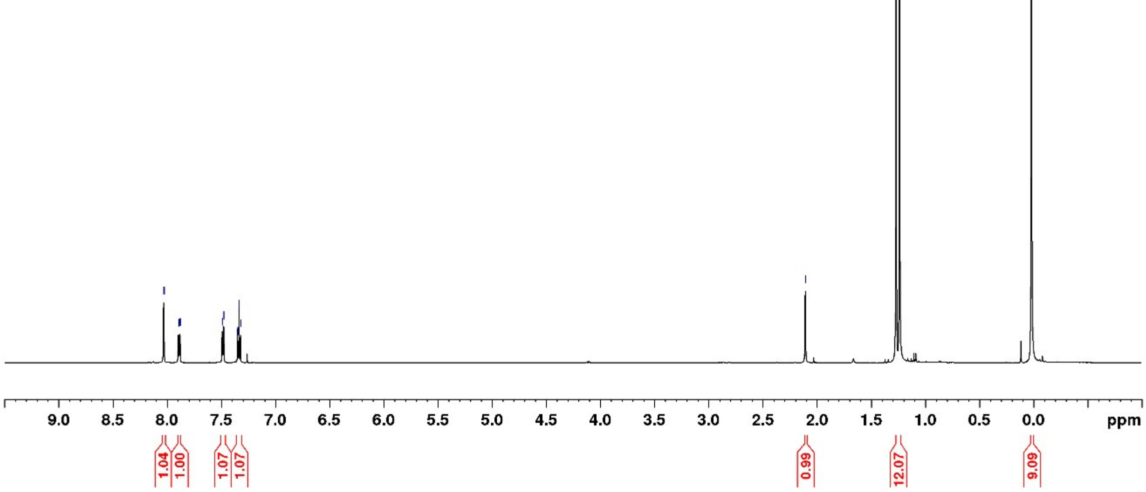

^{13}C NMR, 150 MHz, CDCl$_3$:

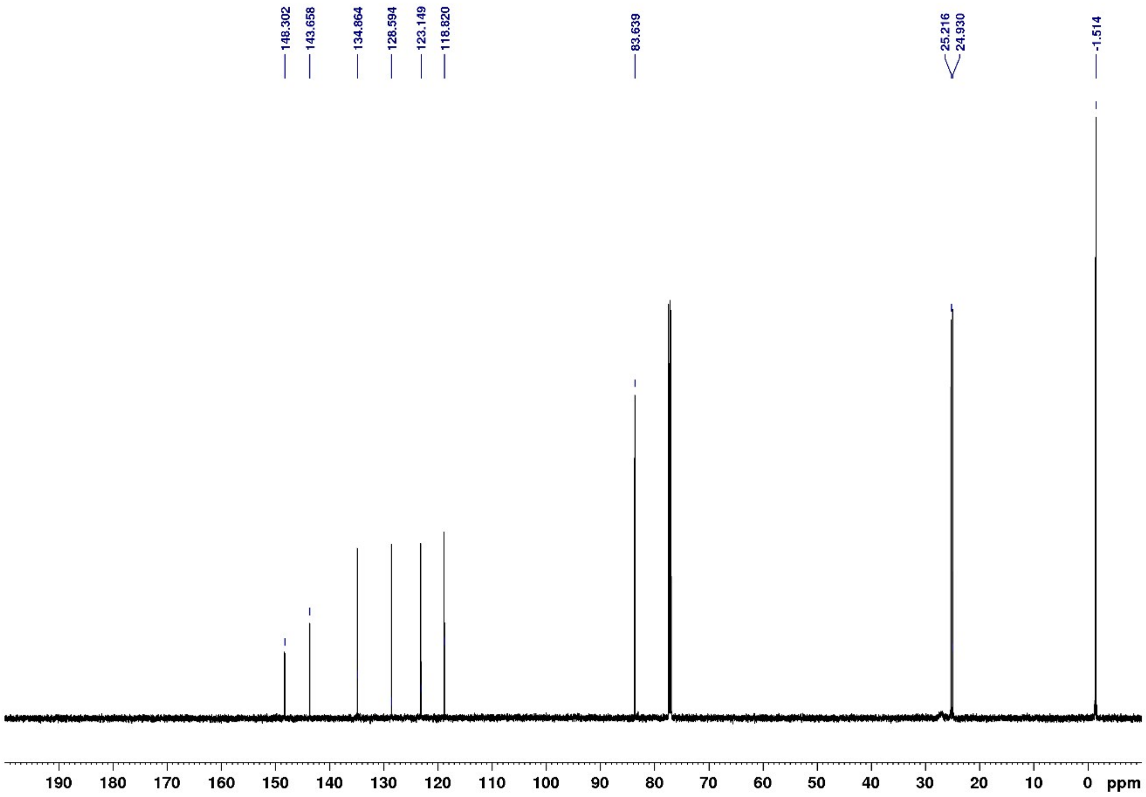

trimethyl((4,4,5,5-tetramethyl-1,3,2-dioxaborolan-2-yl)(2-(trifluoromethoxy)phenyl)methyl)silane (16):

^{1}H NMR, 600 MHz, $CDCl_3$:

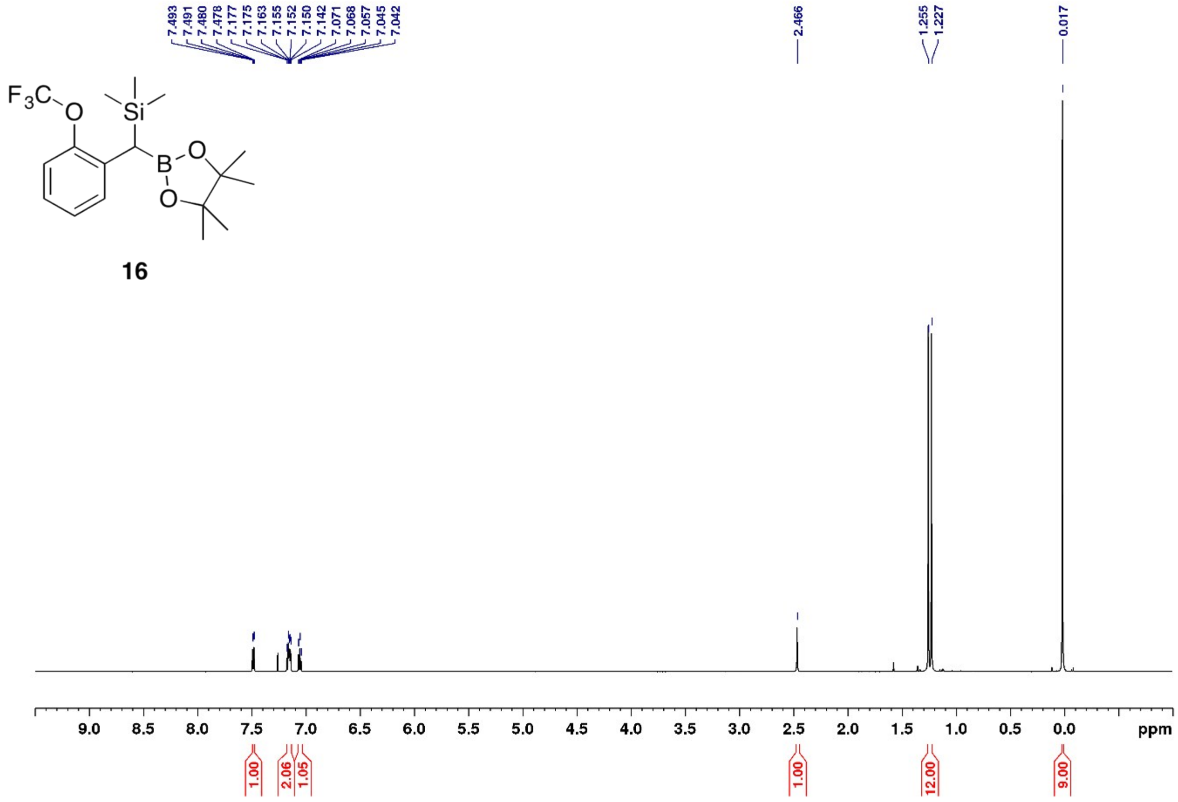

^{13}C NMR, 150 MHz, $CDCl_3$:

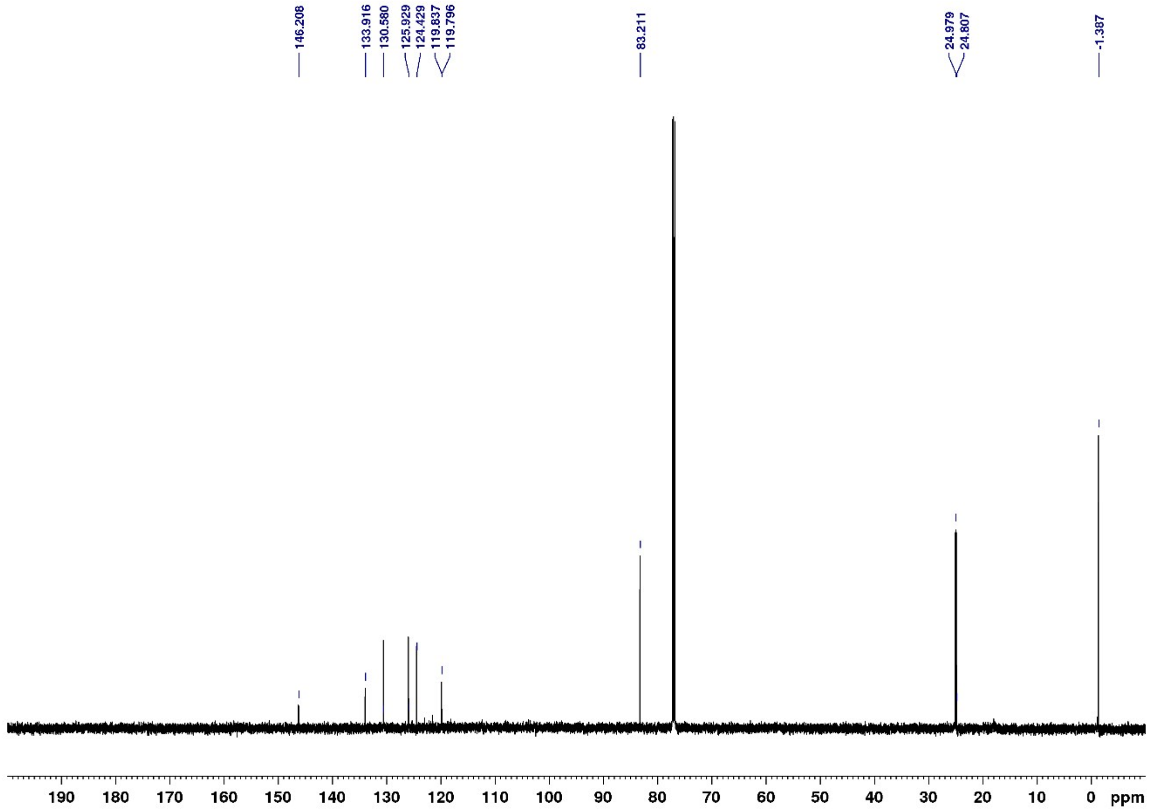

((4-bromophenyl)(4,4,5,5-tetramethyl-1,3,2-dioxaborolan-2-yl)methyl)trimethylsilane (17):

^{1}H NMR, 600 MHz, $CDCl_3$:

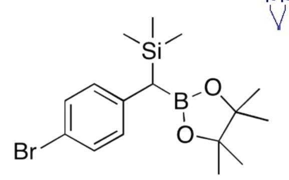

17

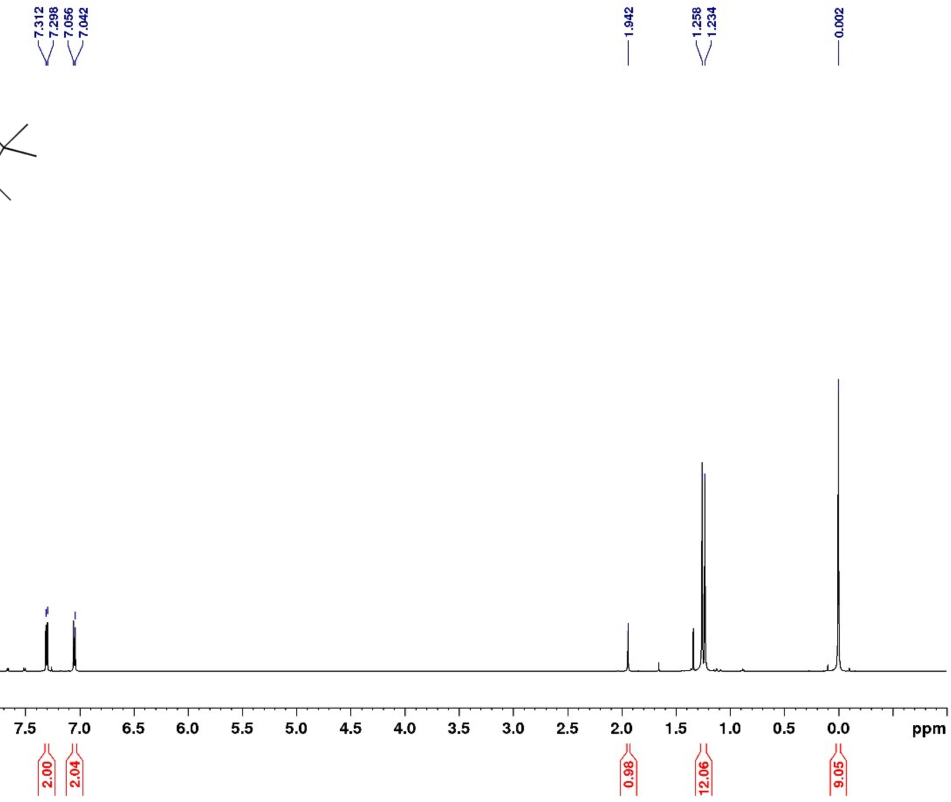

^{13}C NMR, 150 MHz, $CDCl_3$:

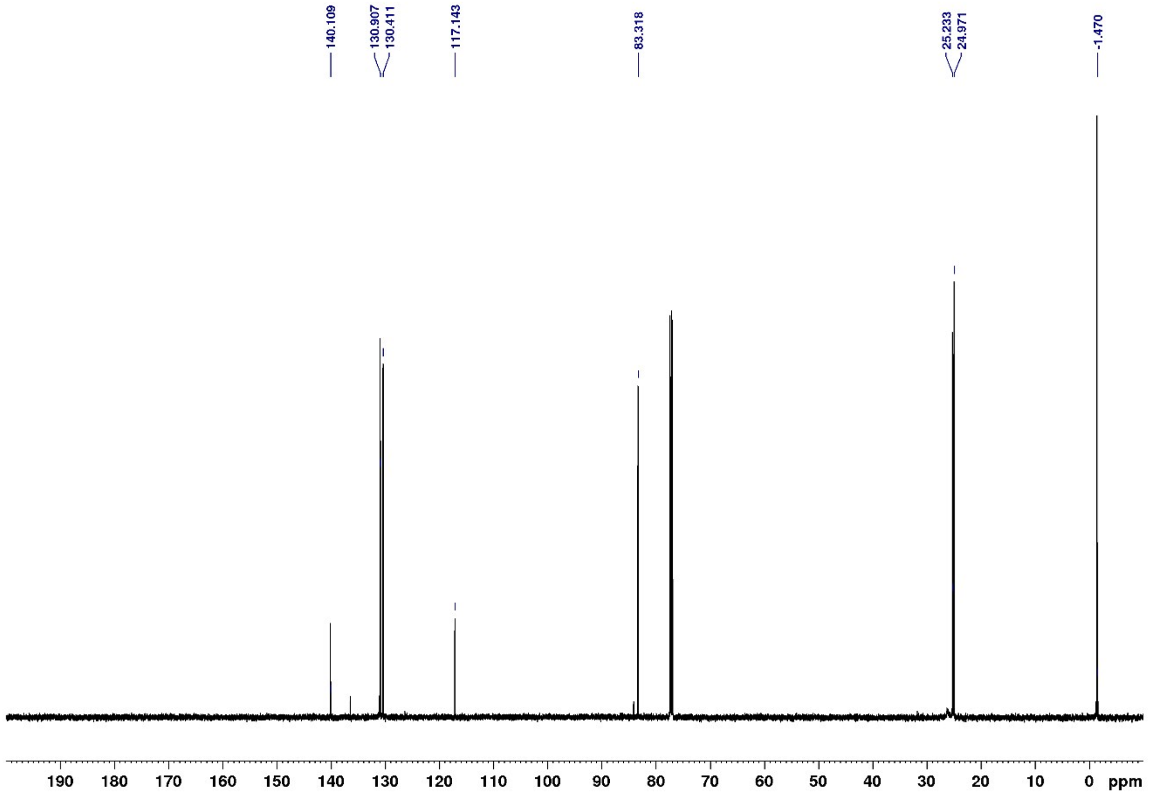

((4-chlorophenyl)(4,4,5,5-tetramethyl-1,3,2-dioxaborolan-2-yl)methyl)trimethylsilane (18):

^{1}H NMR, 600 MHz, CDCl$_3$:

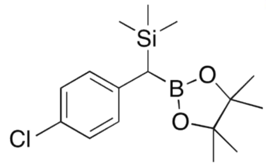

18

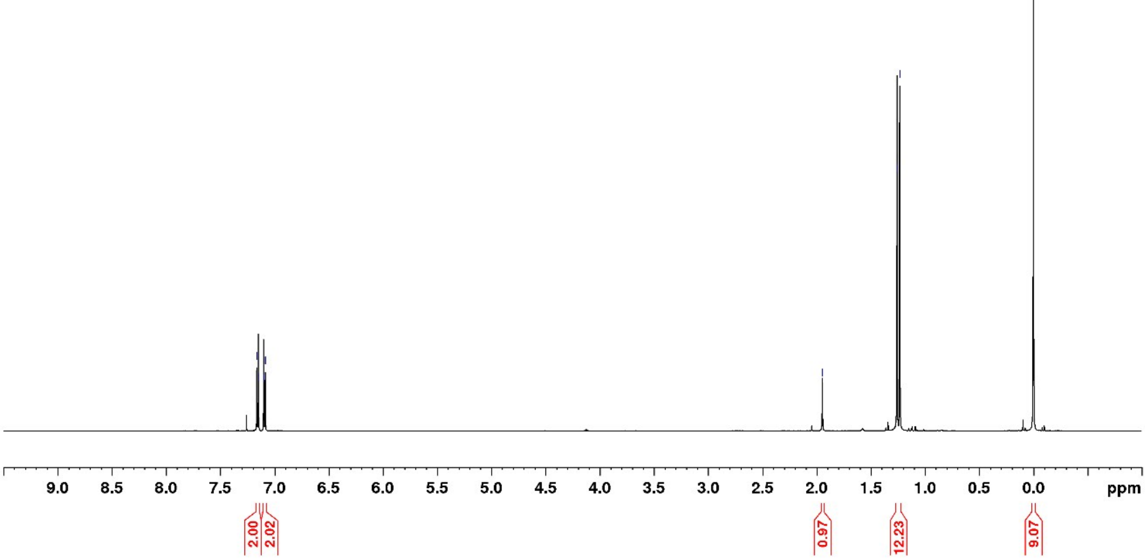

^{13}C NMR, 150 MHz, CDCl$_3$:

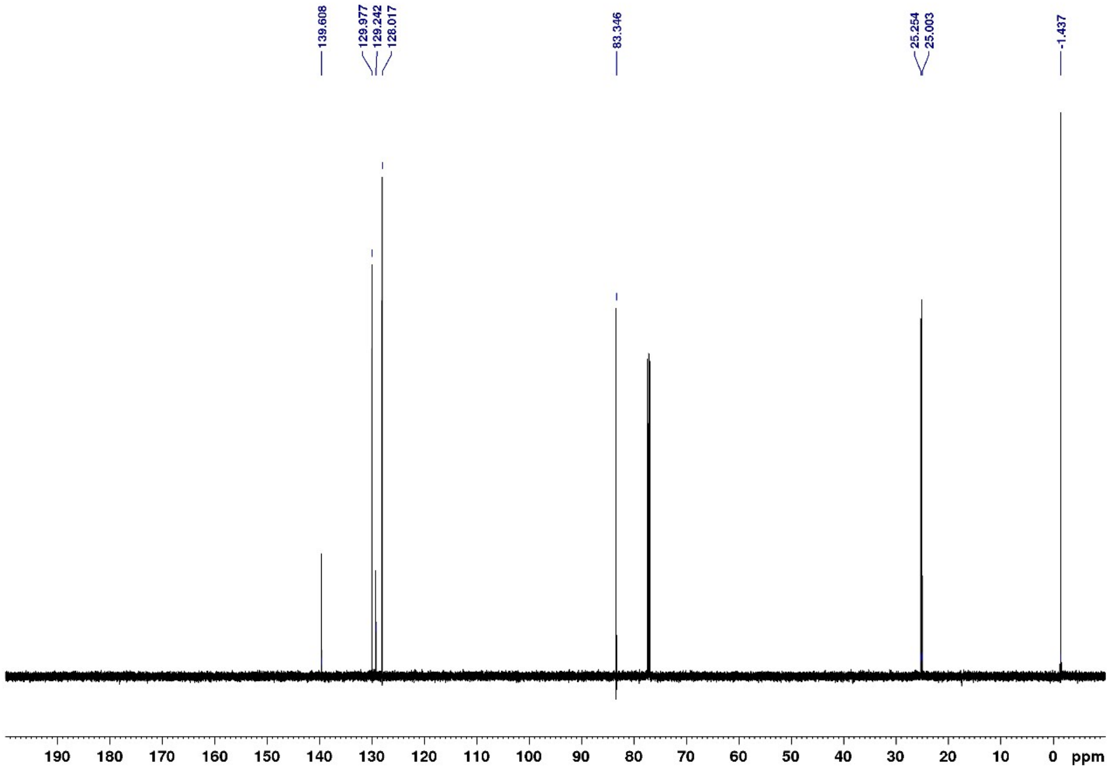

trimethyl(phenyl(4,4,5,5-tetramethyl-1,3,2-dioxaborolan-2-yl)methyl)silane (19):

^{1}H NMR, 600 MHz, $CDCl_3$:

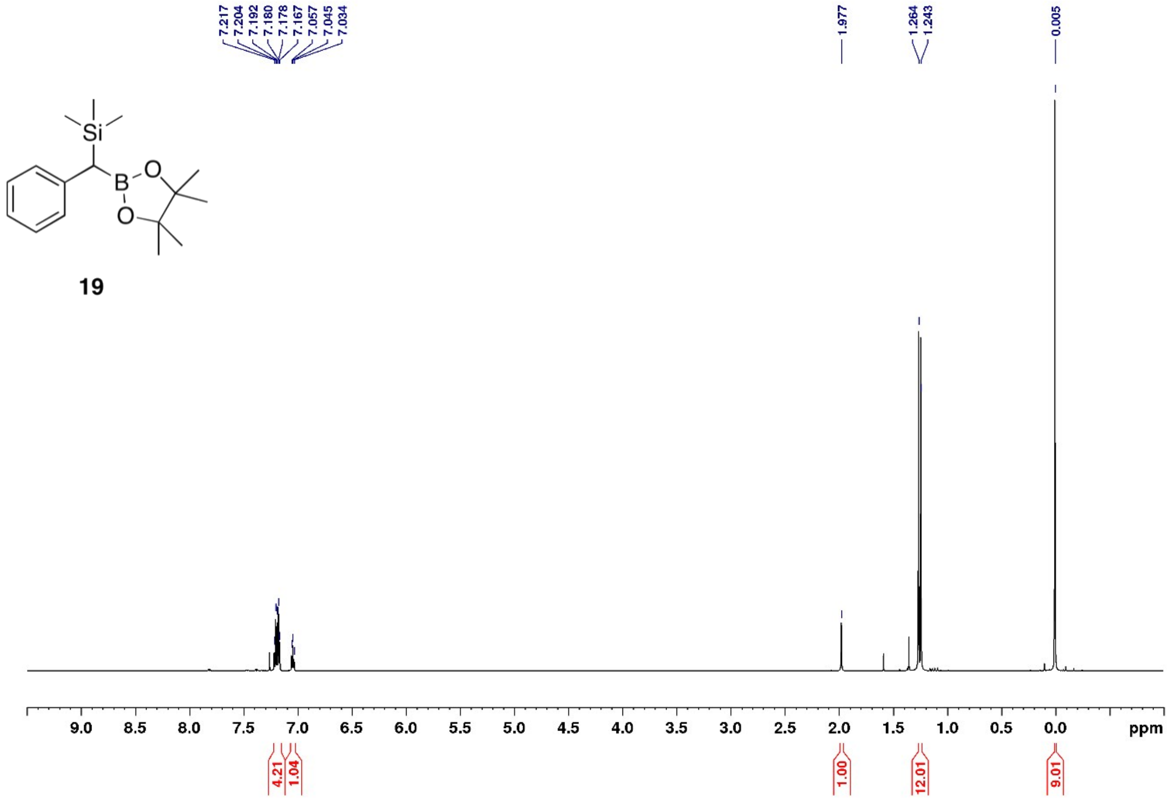

19

^{13}C NMR, 150 MHz, $CDCl_3$:

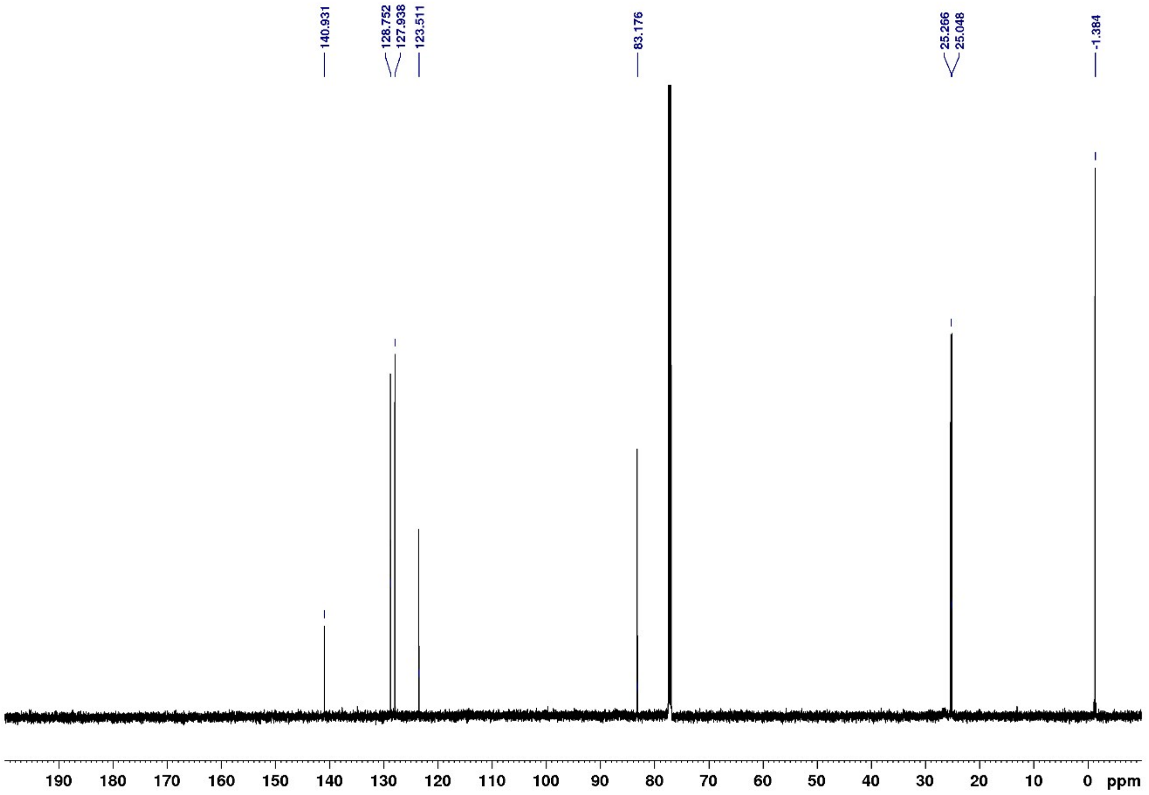

((4-(*tert*-butyl)phenyl)(4,4,5,5-tetramethyl-1,3,2-dioxaborolan-2-yl)methyl)trimethylsilane (20):

^{1}H NMR, 600 MHz, $CDCl_3$:

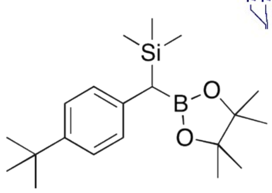

20

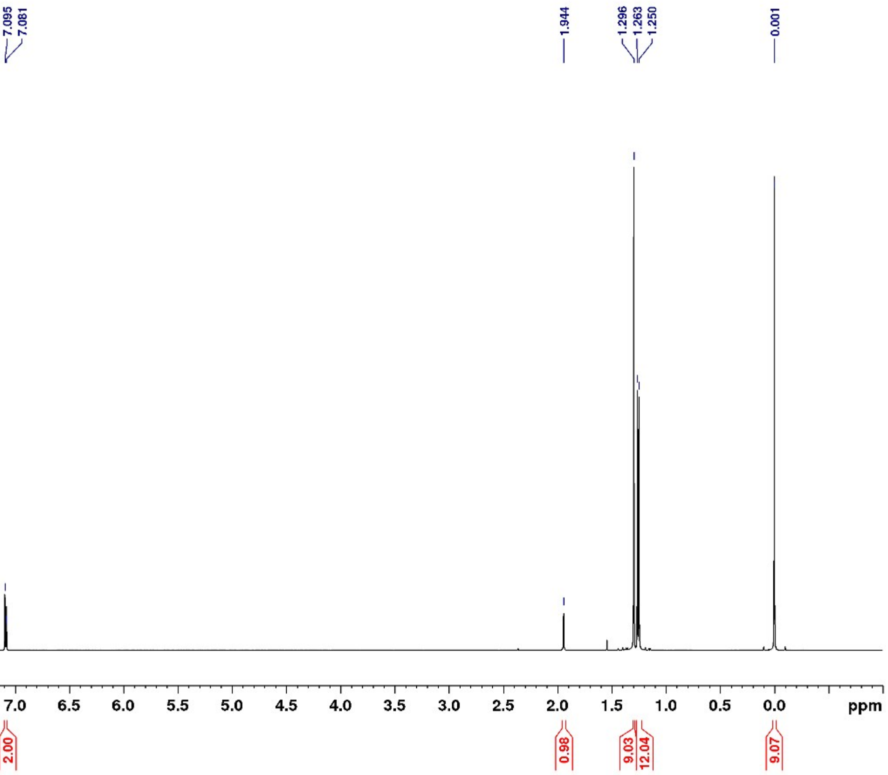

^{13}C NMR, 150 MHz, $CDCl_3$:

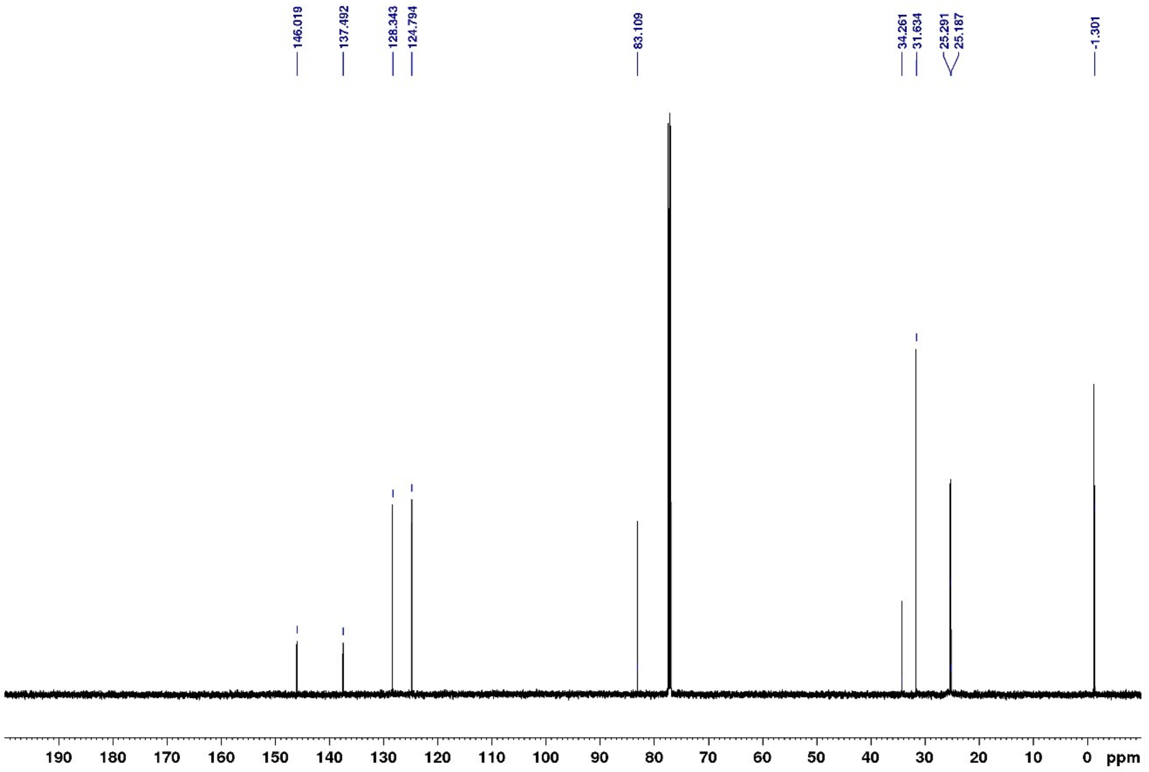

((4-fluorophenyl)(4,4,5,5-tetramethyl-1,3,2-dioxaborolan-2-yl)methyl)trimethylsilane (21):

^{1}H NMR, 600 MHz, CDCl$_3$:

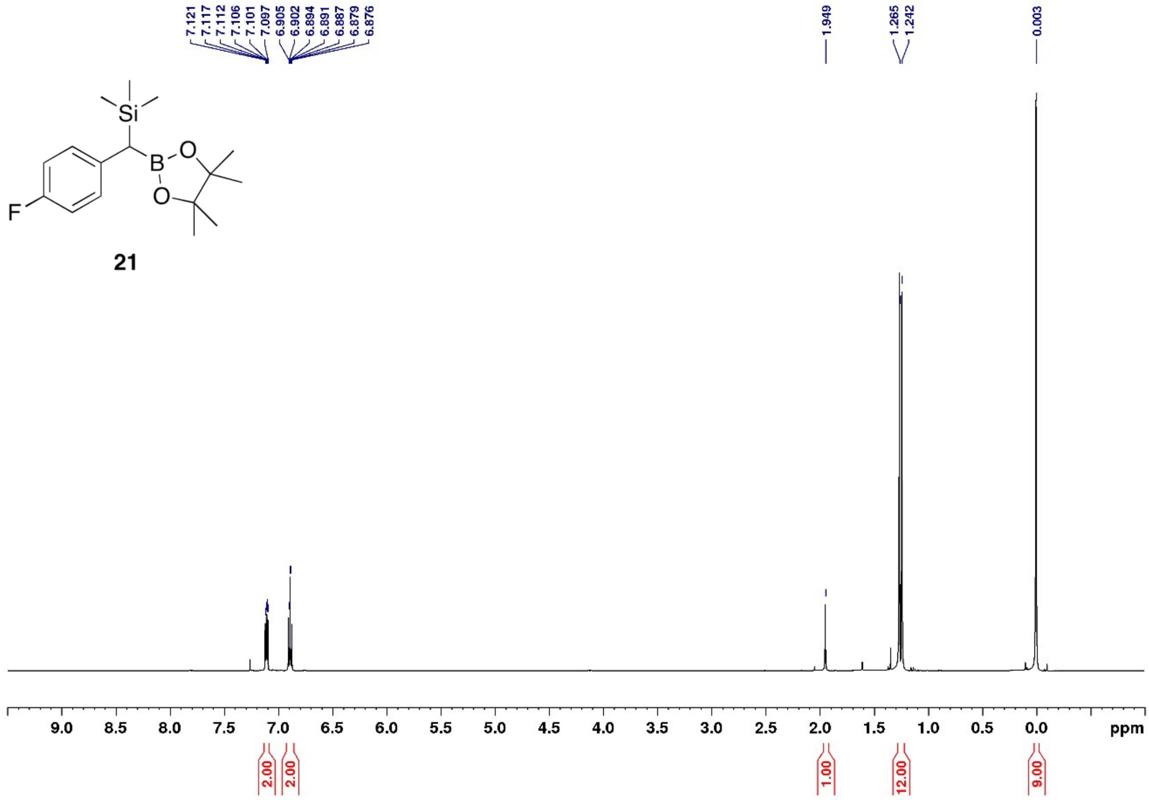

^{13}C NMR, 150 MHz, CDCl$_3$:

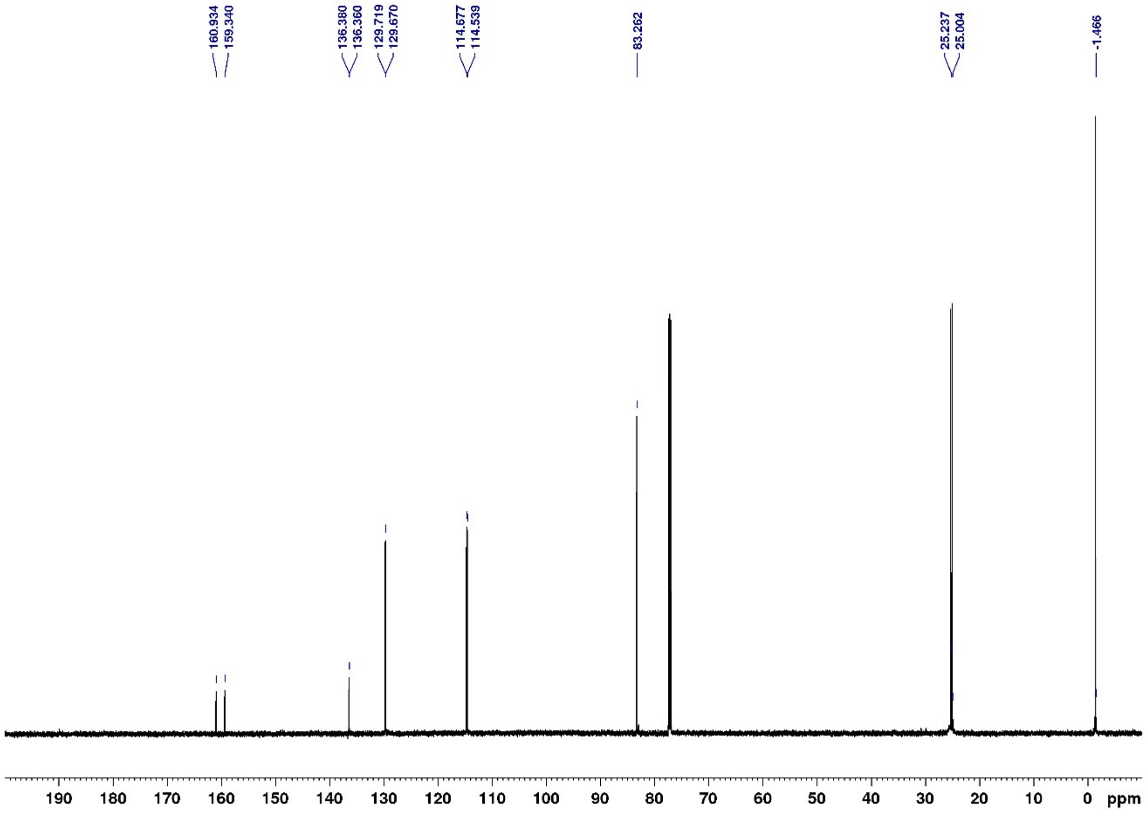

trimethyl((4,4,5,5-tetramethyl-1,3,2-dioxaborolan-2-yl)(*o*-tolyl)methyl)silane (22):

^{1}H NMR, 600 MHz, $CDCl_3$:

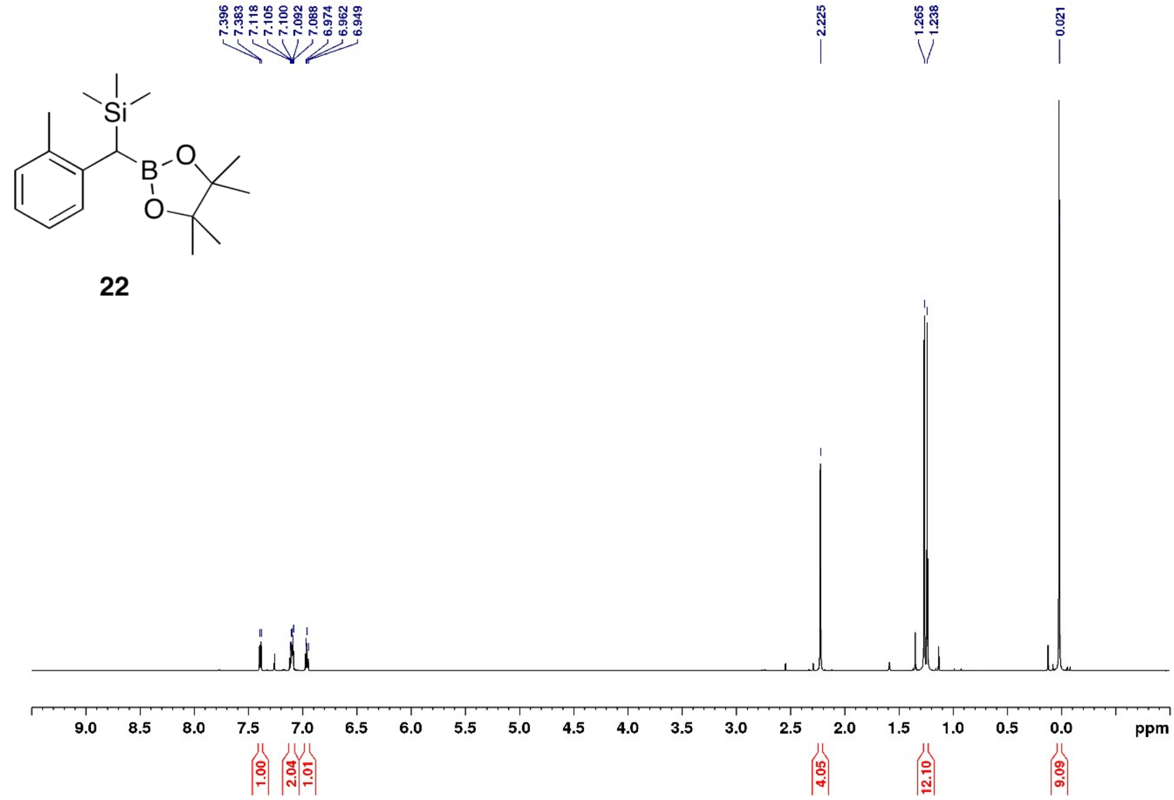

^{13}C NMR, 150 MHz, $CDCl_3$:

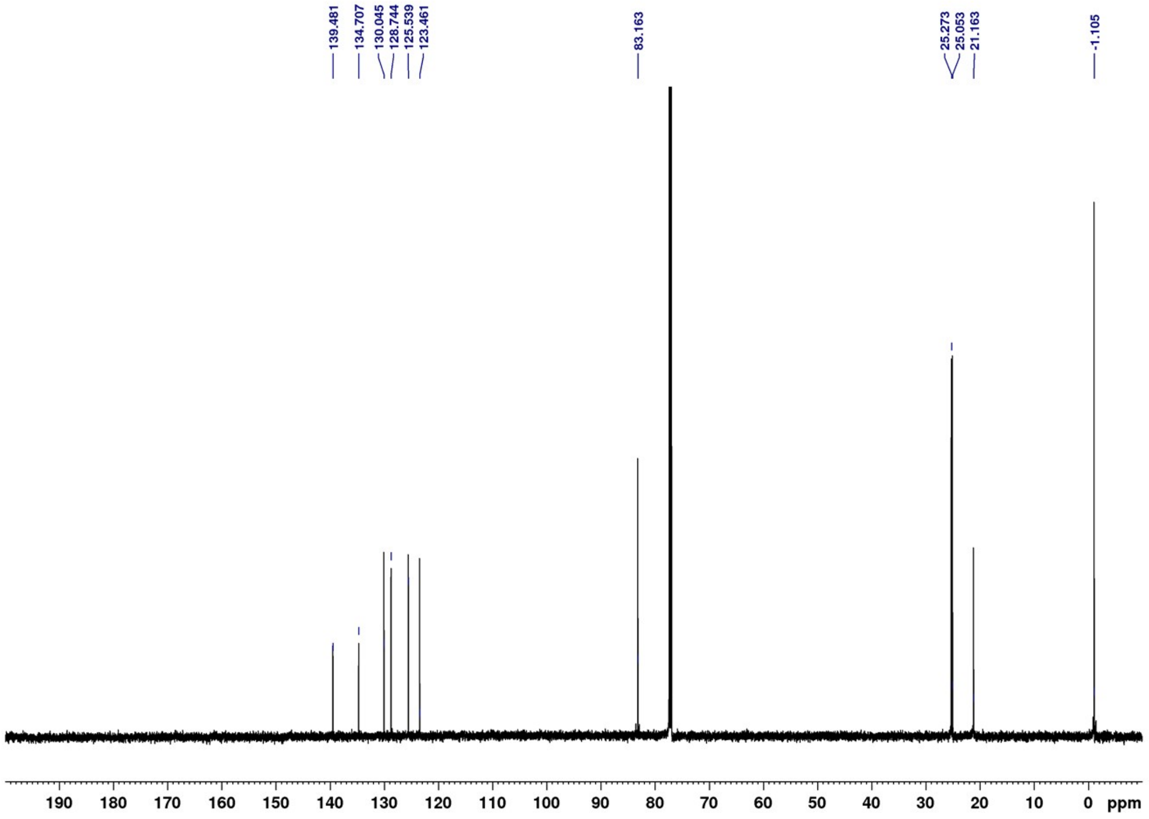

trimethyl((4,4,5,5-tetramethyl-1,3,2-dioxaborolan-2-yl)(4-(trifluoromethyl)phenyl)methyl)silane (23):

^{1}H NMR, 600 MHz, CDCl$_3$:

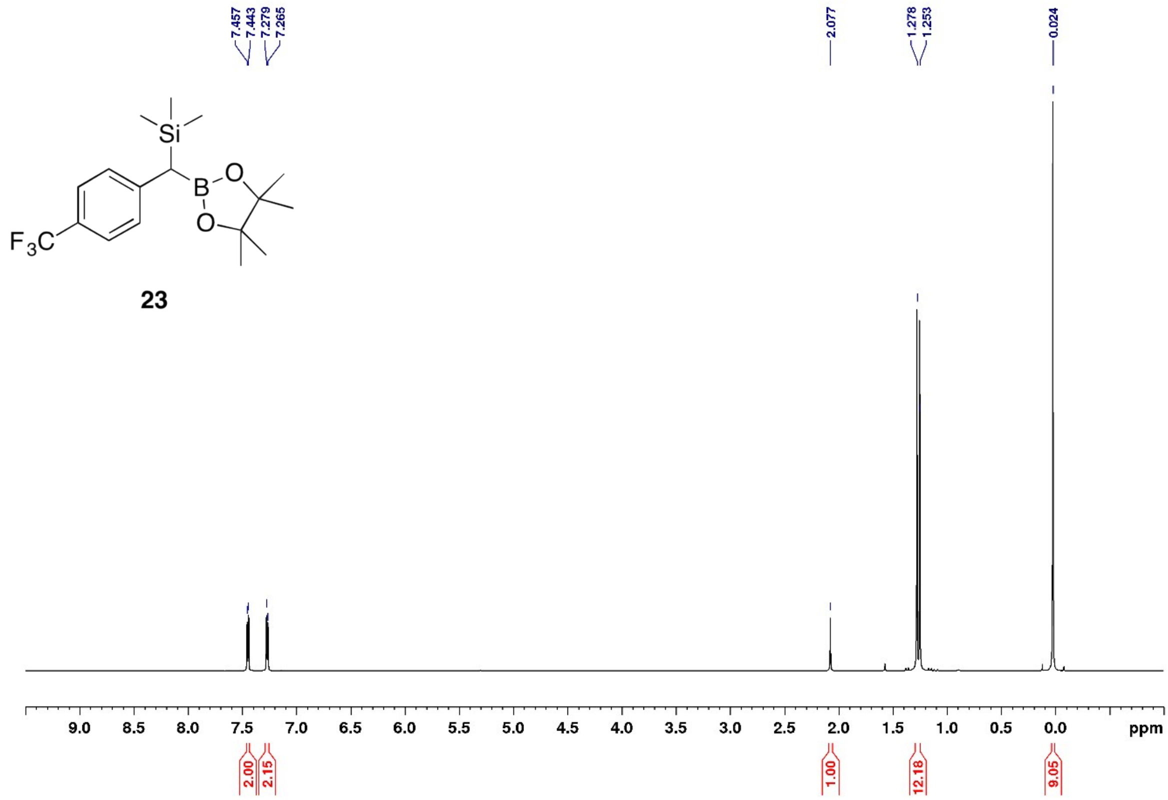

^{13}C NMR, 150 MHz, CDCl$_3$:

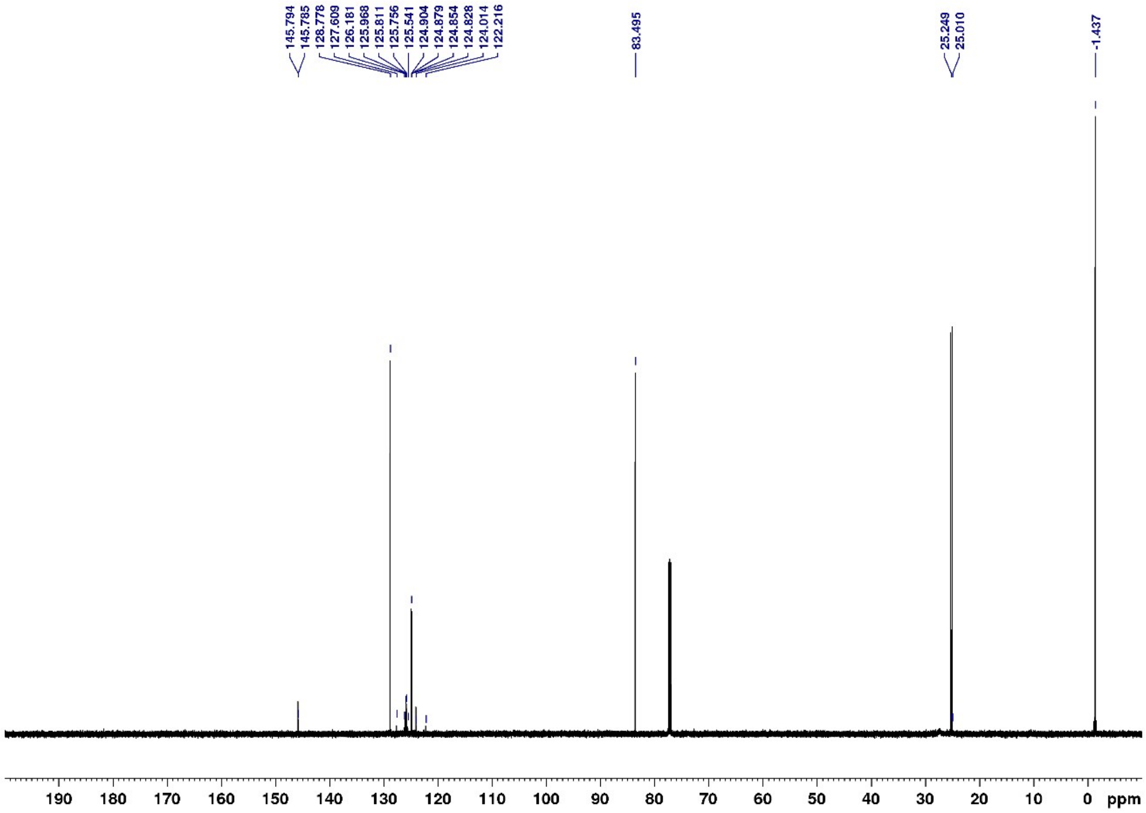

***N*-(3-((4,4,5,5-tetramethyl-1,3,2-dioxaborolan-2-yl)(trimethylsilyl)methyl)phenyl)acetamide (24):**

^{1}H NMR, 600 MHz, $CDCl_3$:

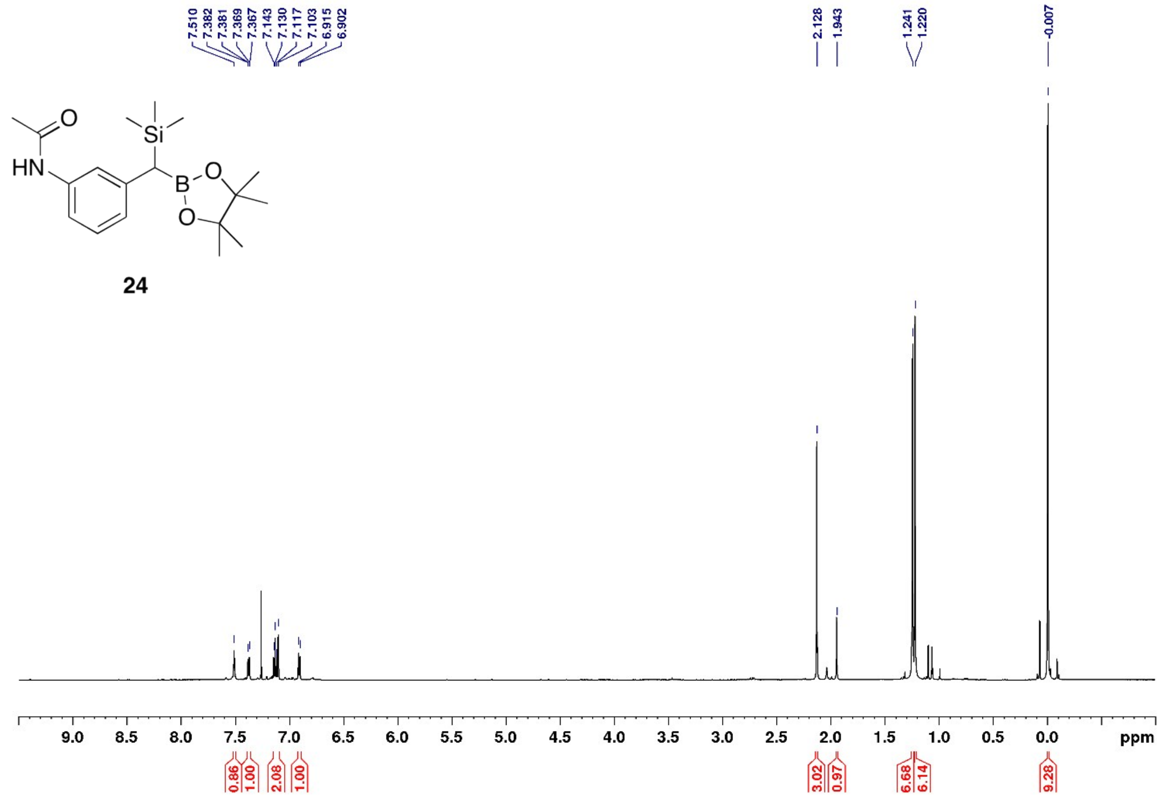

^{13}C NMR, 150 MHz, $CDCl_3$:

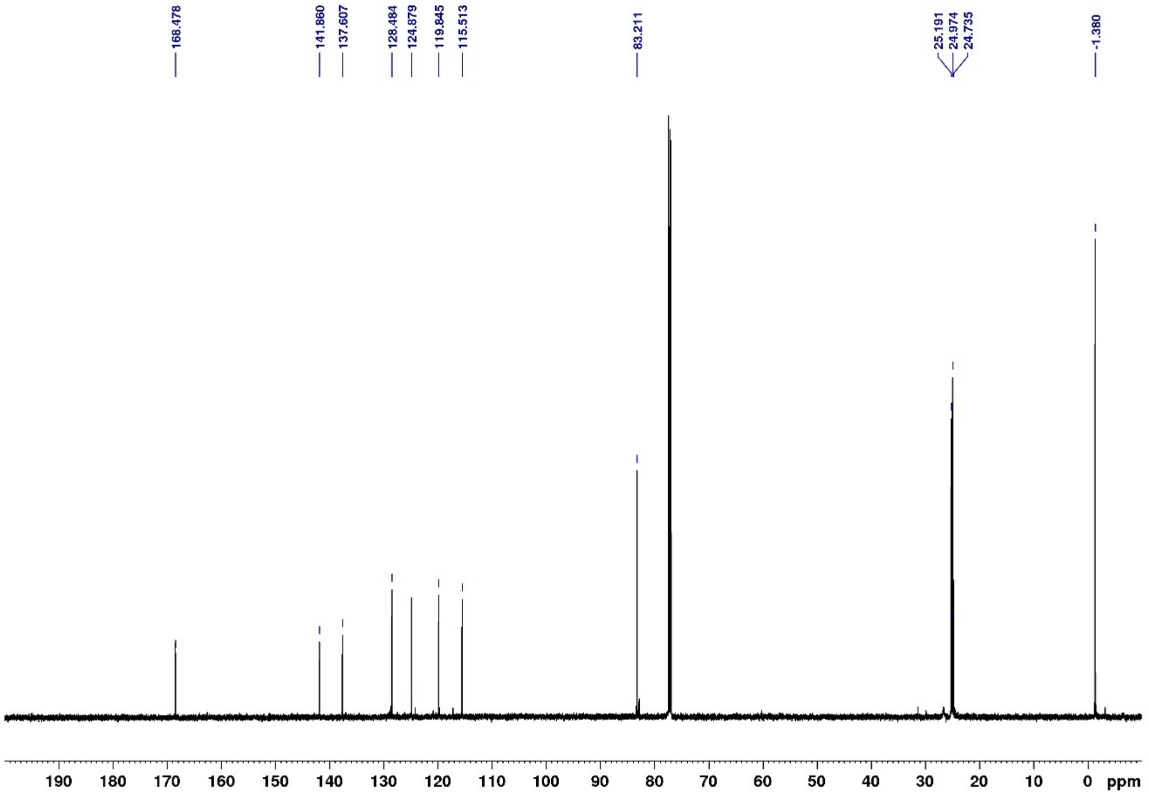

trimethyl((4-methyl-3-nitrophenyl)(4,4,5,5-tetramethyl-1,3,2-dioxaborolan-2-yl)methyl)silane (25):

^{1}H NMR, 600 MHz, $CDCl_3$:

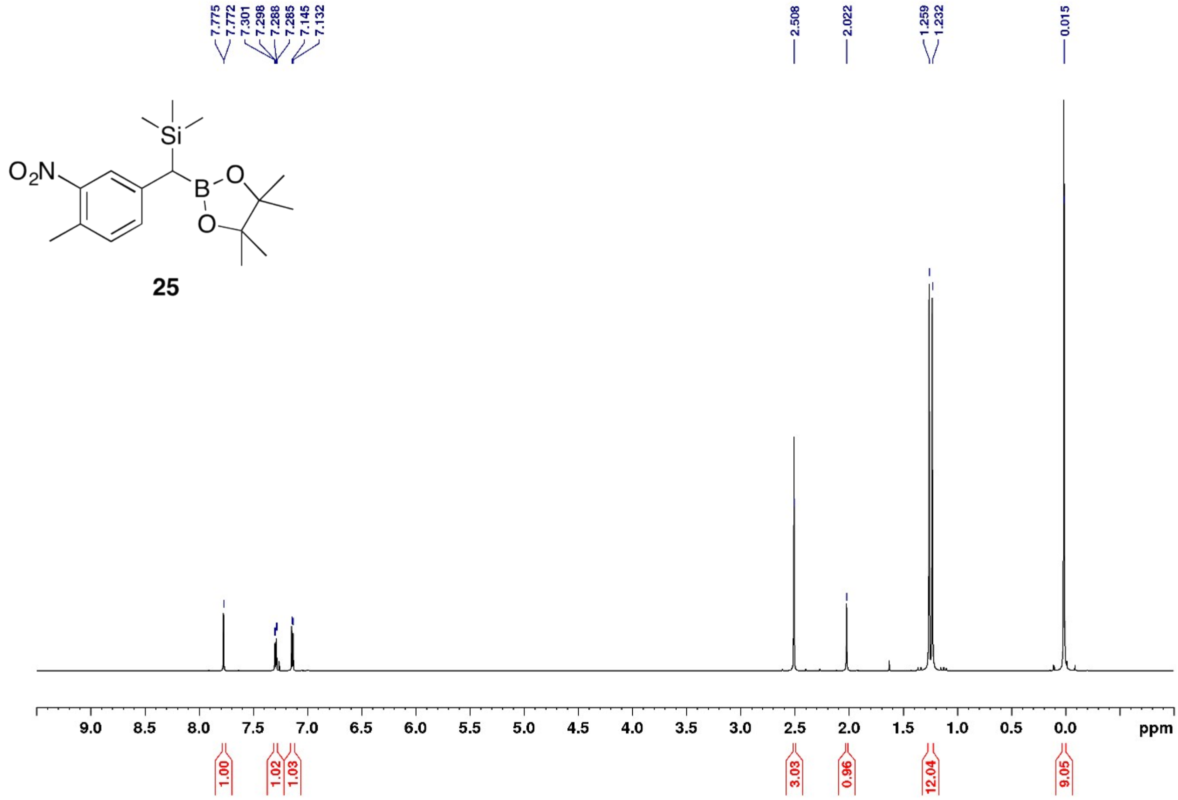

^{13}C NMR, 150 MHz, $CDCl_3$:

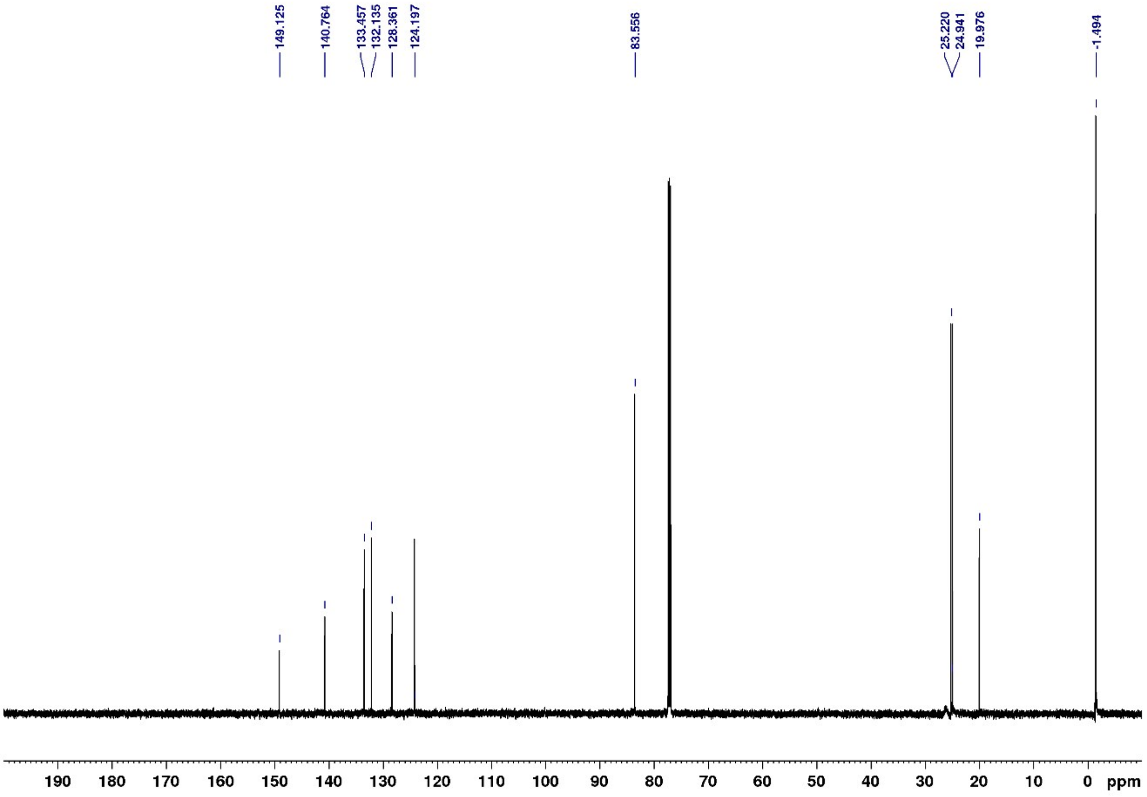

trimethyl((4,4,5,5-tetramethyl-1,3,2-dioxaborolan-2-yl)(4-vinylphenyl)methyl)silane (26):

^{1}H NMR, 600 MHz, $CDCl_3$:

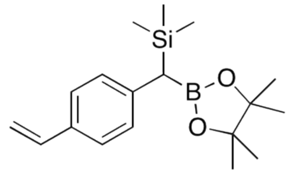

26

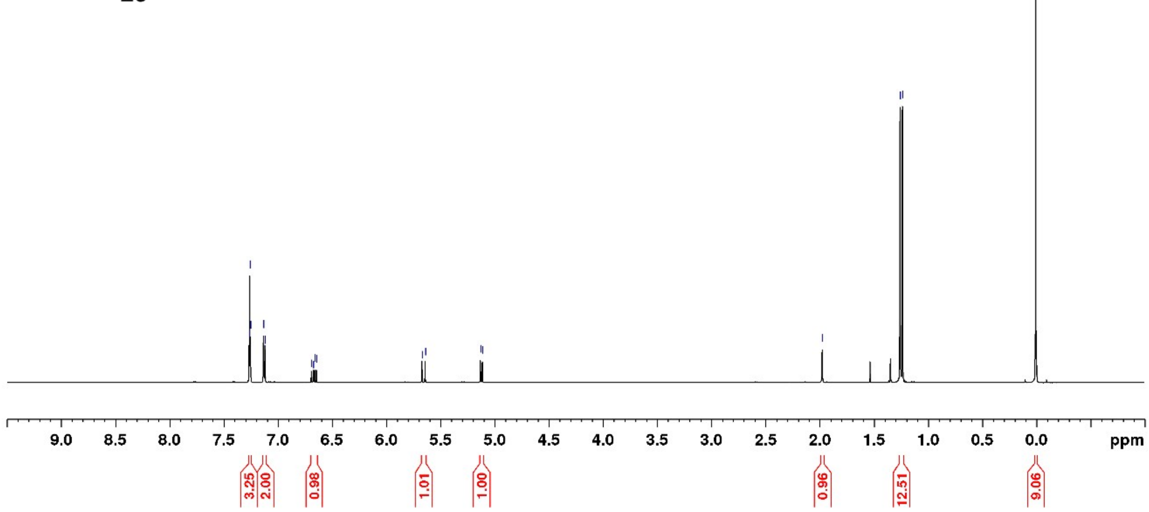

^{13}C NMR, 150 MHz, $CDCl_3$:

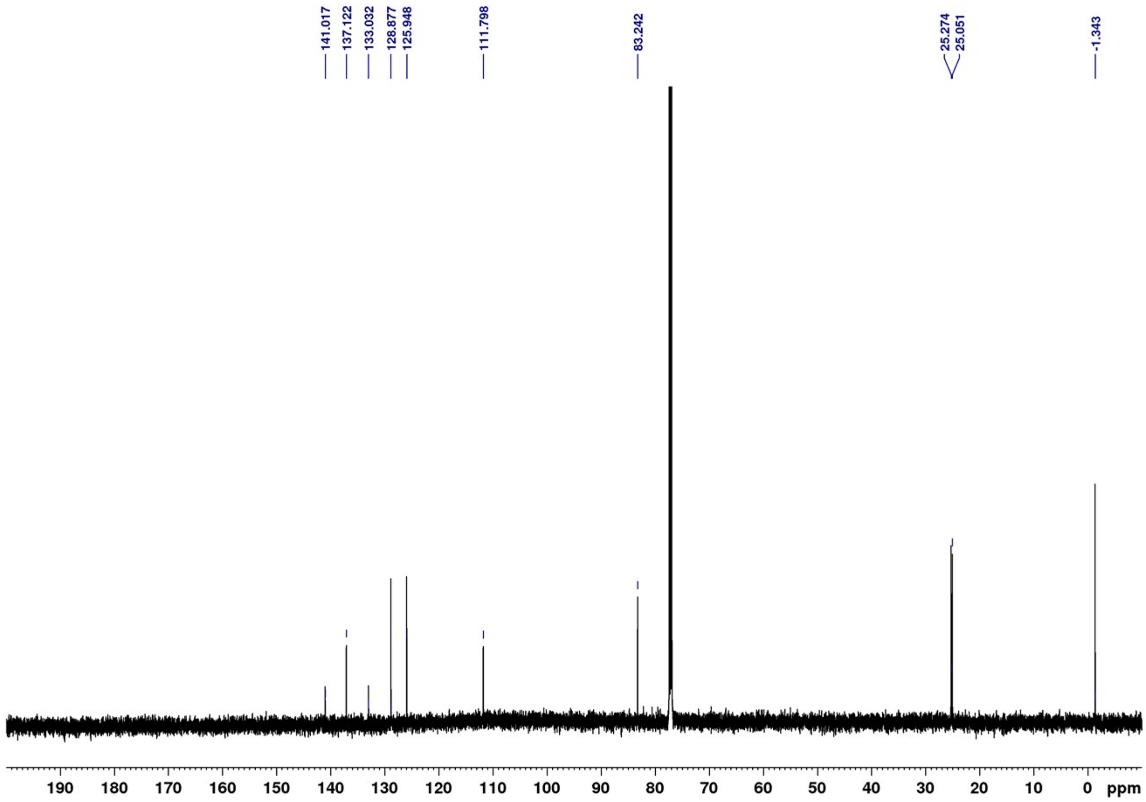

trimethyl((4,4,5,5-tetramethyl-1,3,2-dioxaborolan-2-yl)(thiophen-2-yl)methyl)silane (27):

^{1}H NMR, 600 MHz, CDCl$_3$:

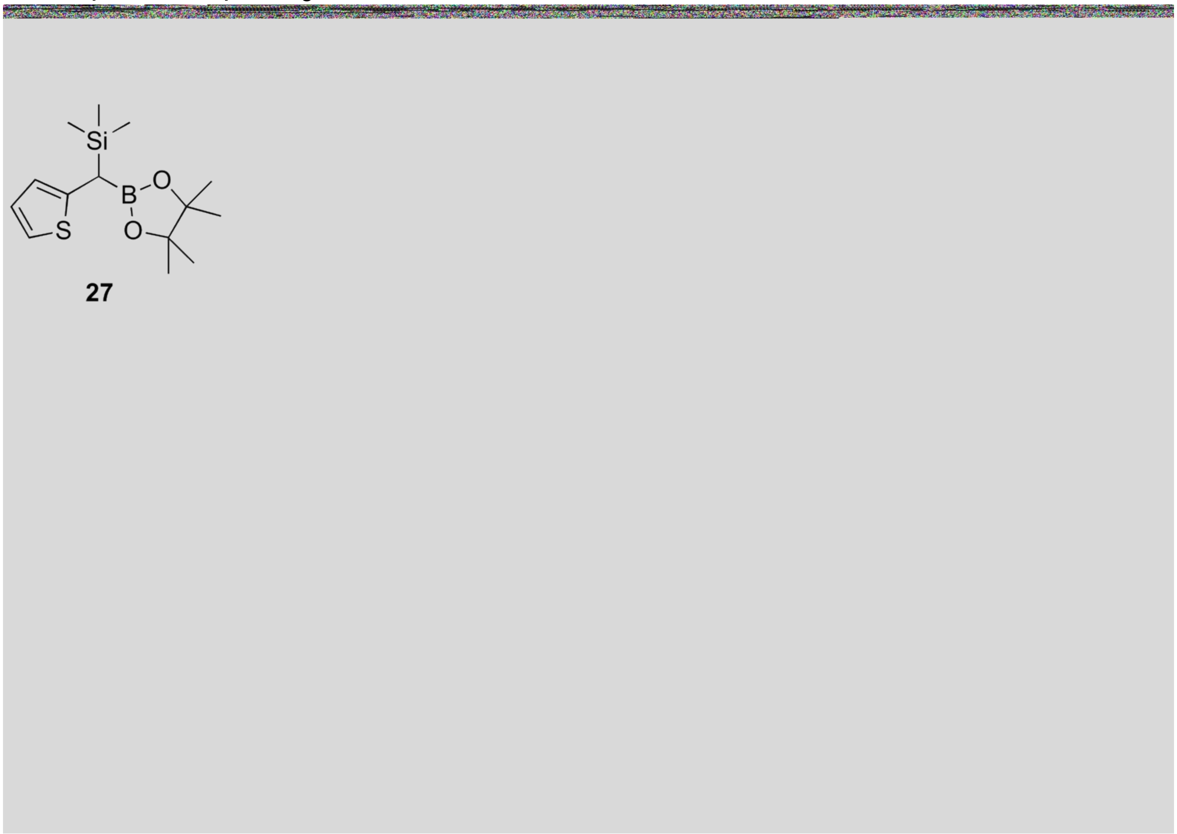

^{13}C NMR, 150 MHz, CDCl$_3$:

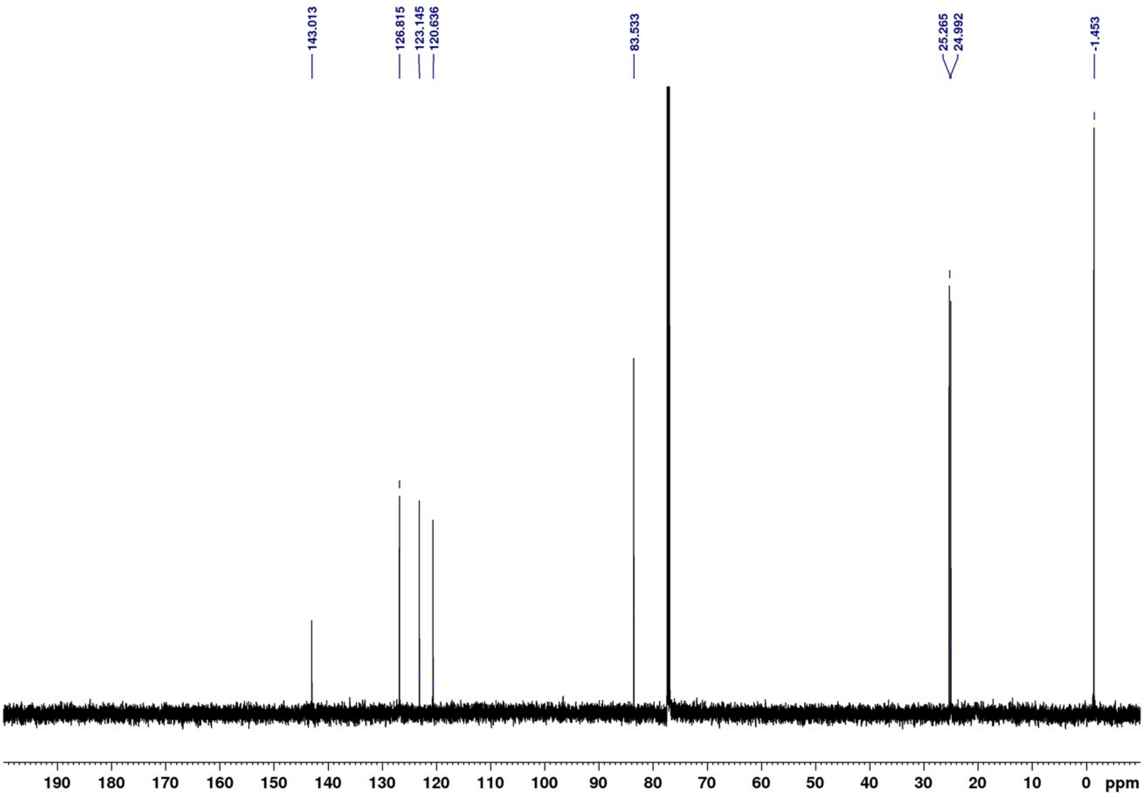

((3,5-di-*tert*-butylphenyl)(4,4,5,5-tetramethyl-1,3,2-dioxaborolan-2-yl)methyl)trimethylsilane (28):

^{1}H NMR, 600 MHz, $CDCl_3$:

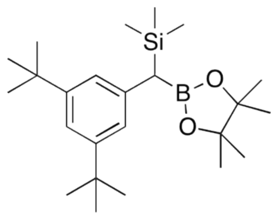

28

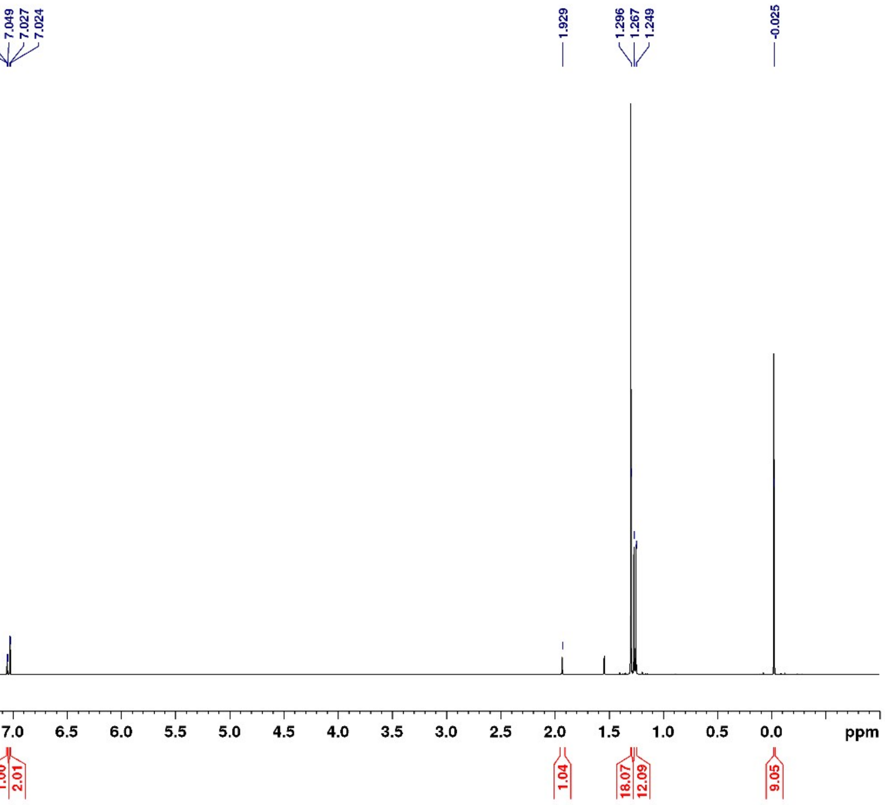

^{13}C NMR, 150 MHz, $CDCl_3$:

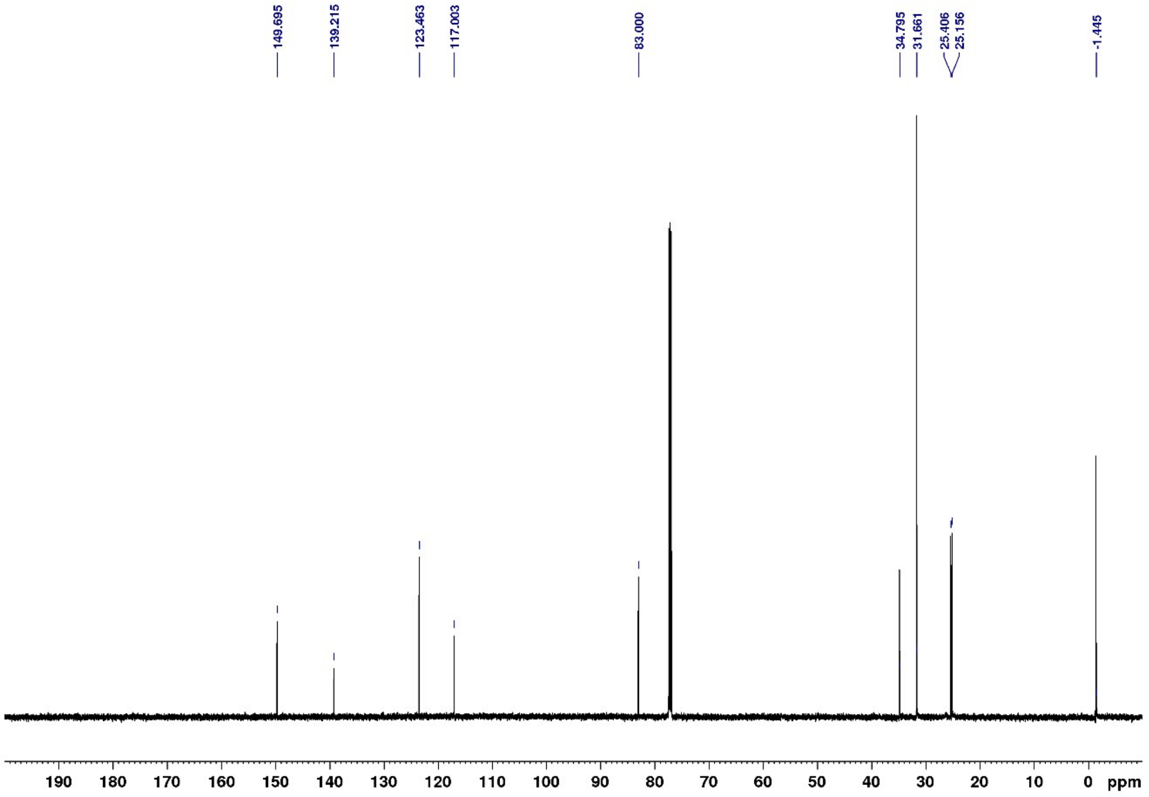

methyl 4-((4,4,5,5-tetramethyl-1,3,2-dioxaborolan-2-yl)(trimethylsilyl)methyl)benzoate (29):

^{1}H NMR, 600 MHz, $CDCl_3$:

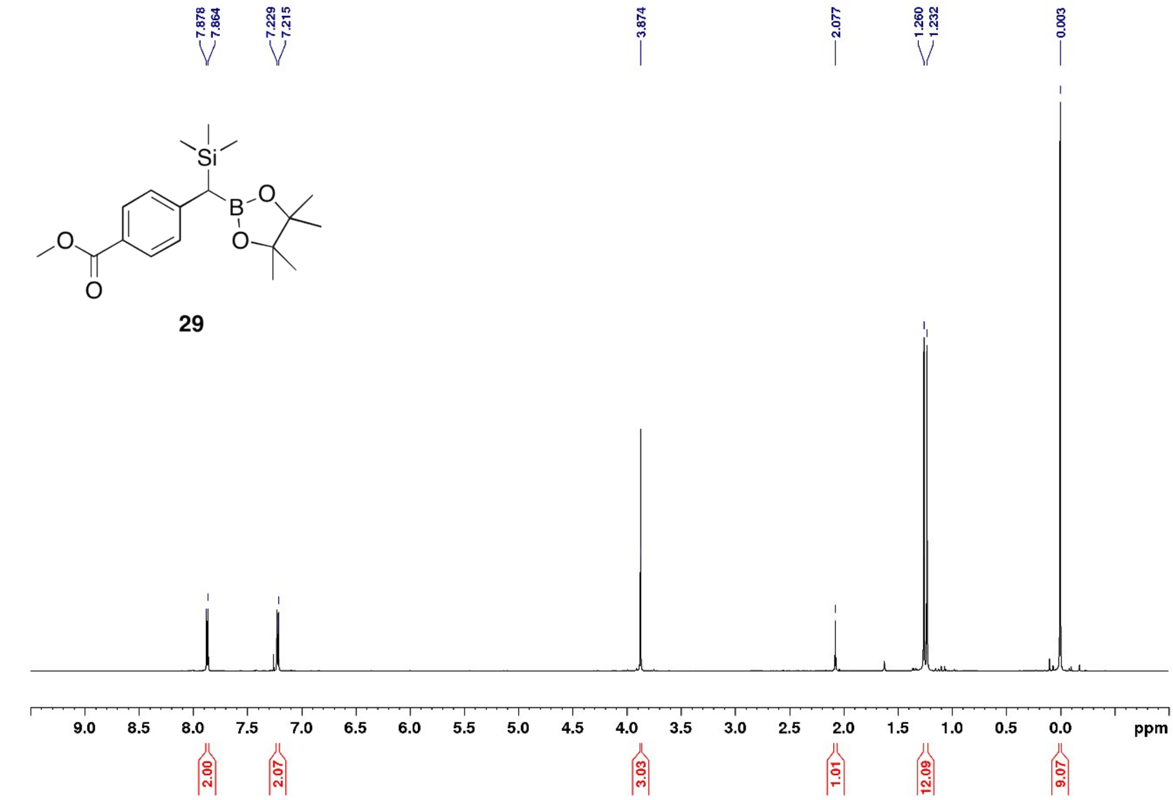

^{13}C NMR, 150 MHz, $CDCl_3$:

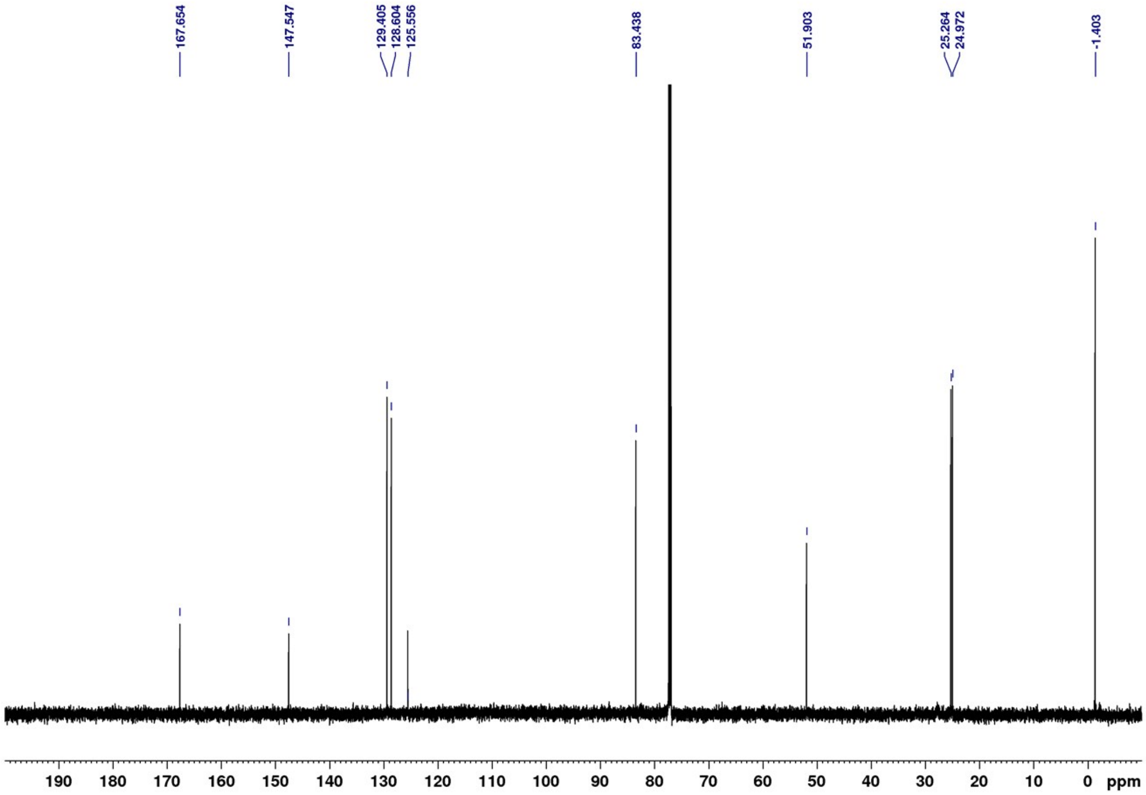

4-((4,4,5,5-tetramethyl-1,3,2-dioxaborolan-2-yl)(trimethylsilyl)methyl)benzonitrile (30):

^{1}H NMR, 600 MHz, $CDCl_3$:

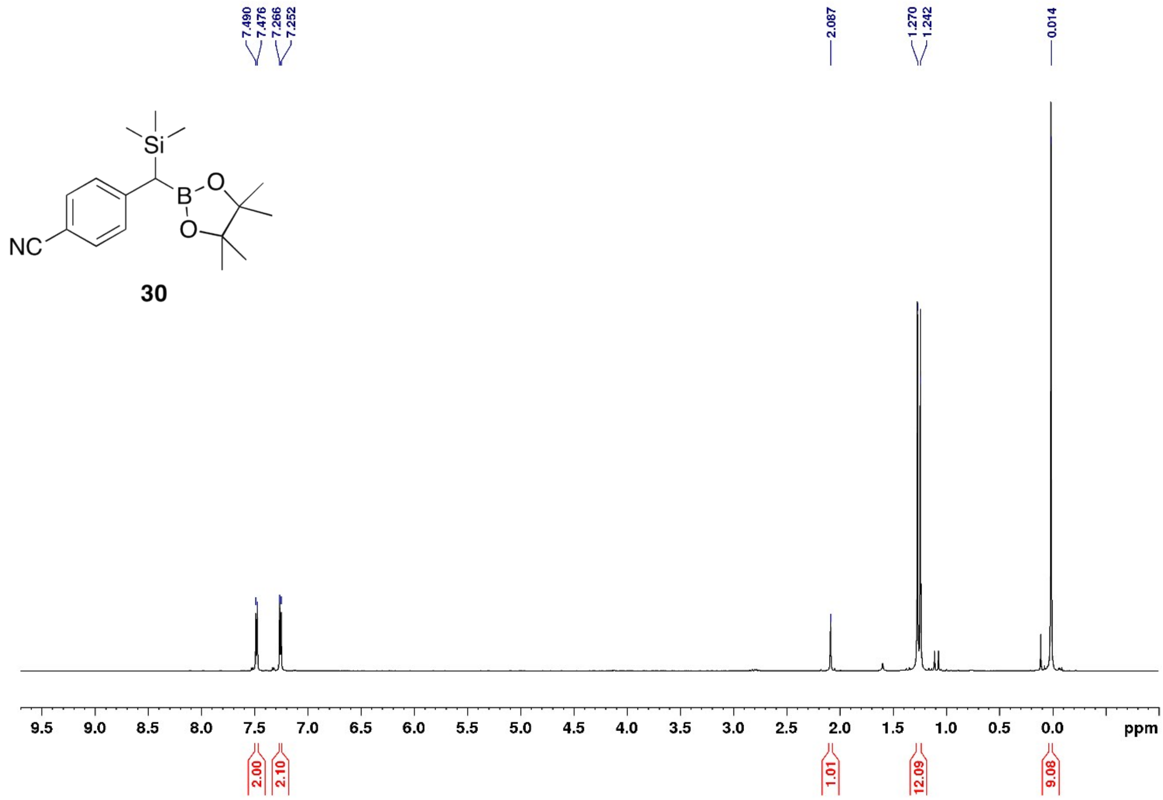

^{13}C NMR, 150 MHz, $CDCl_3$:

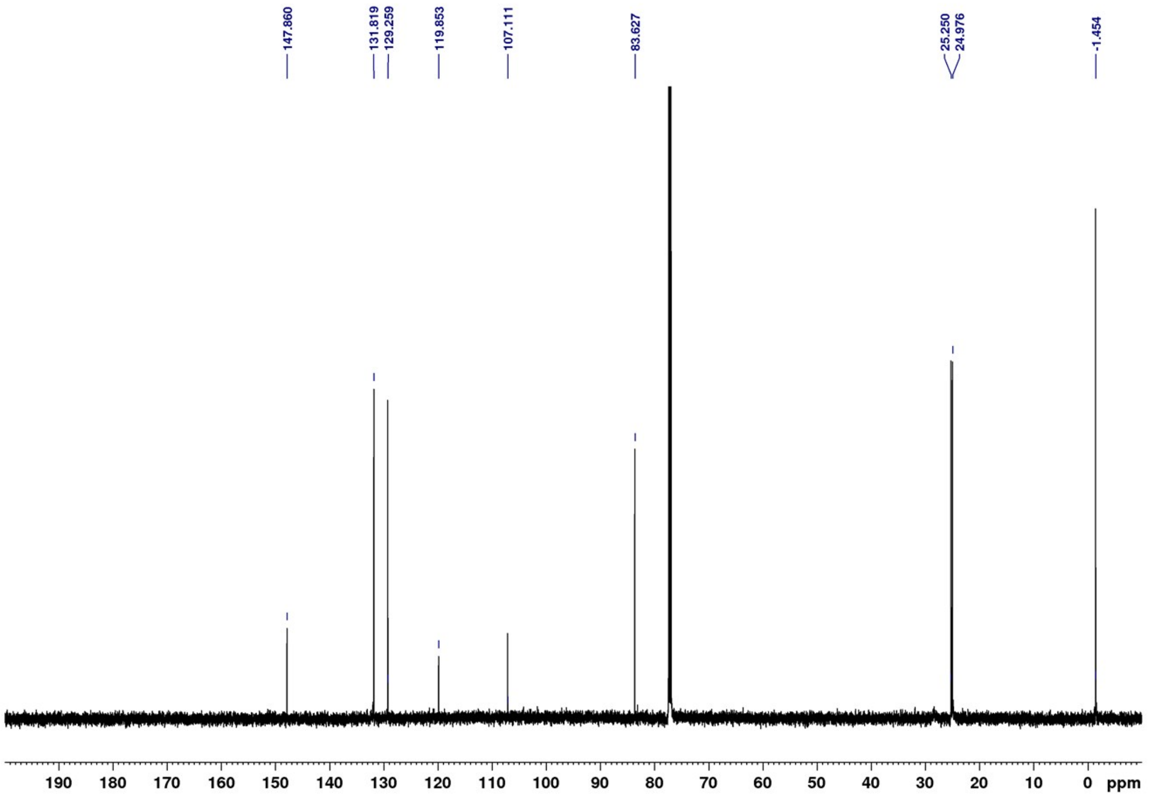

(*E*)-(3-(4-fluorophenyl)-1-(4,4,5,5-tetramethyl-1,3,2-dioxaborolan-2-yl)allyl)trimethylsilane (31):

^{1}H NMR, 600 MHz, $CDCl_3$:

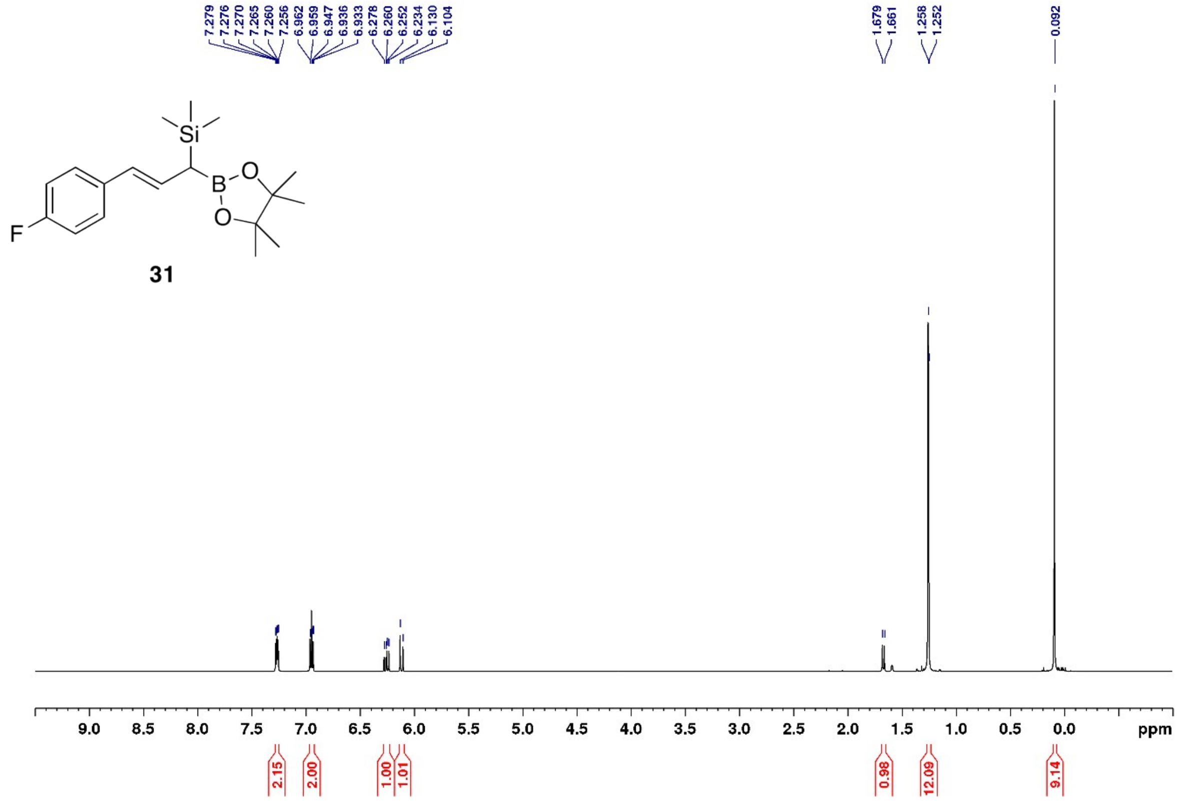

^{13}C NMR, 150 MHz, $CDCl_3$:

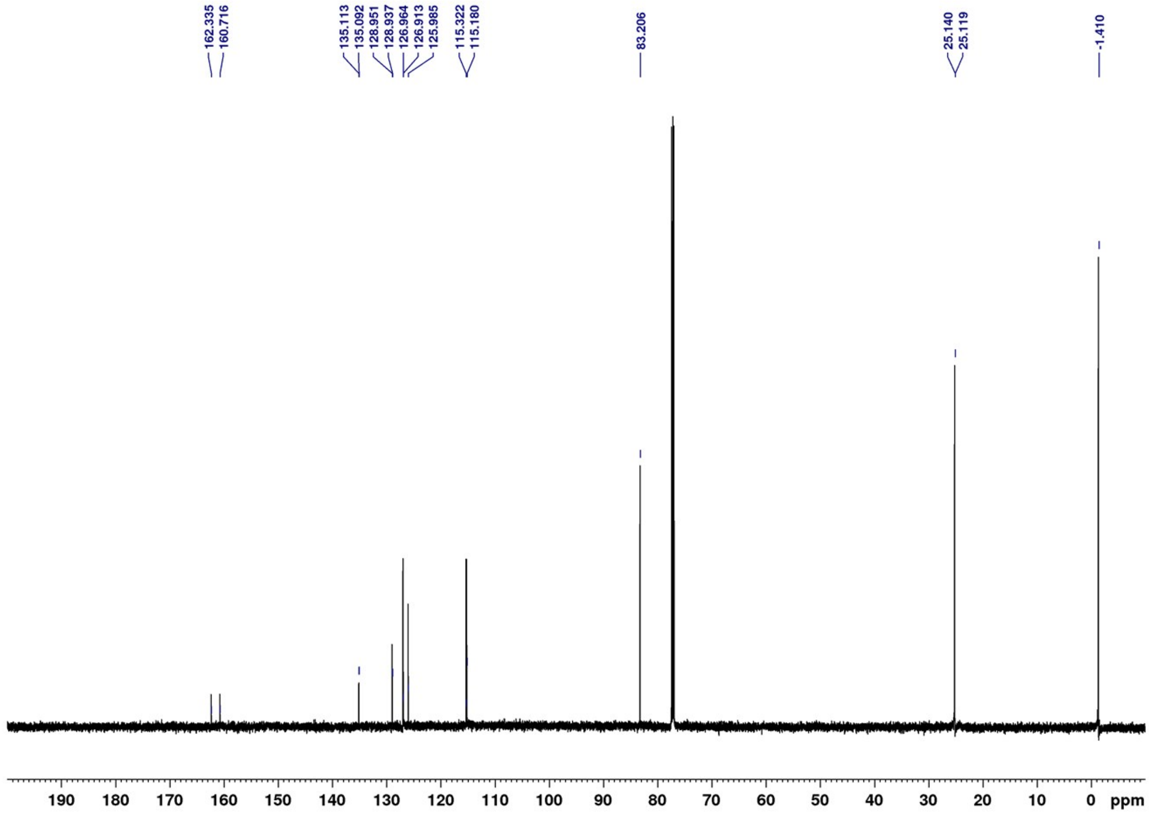

(*E*)-trimethyl(1-(4,4,5,5-tetramethyl-1,3,2-dioxaborolan-2-yl)-3-(*p*-tolyl)allyl)silane (32):

^{1}H NMR, 600 MHz, $CDCl_3$:

32

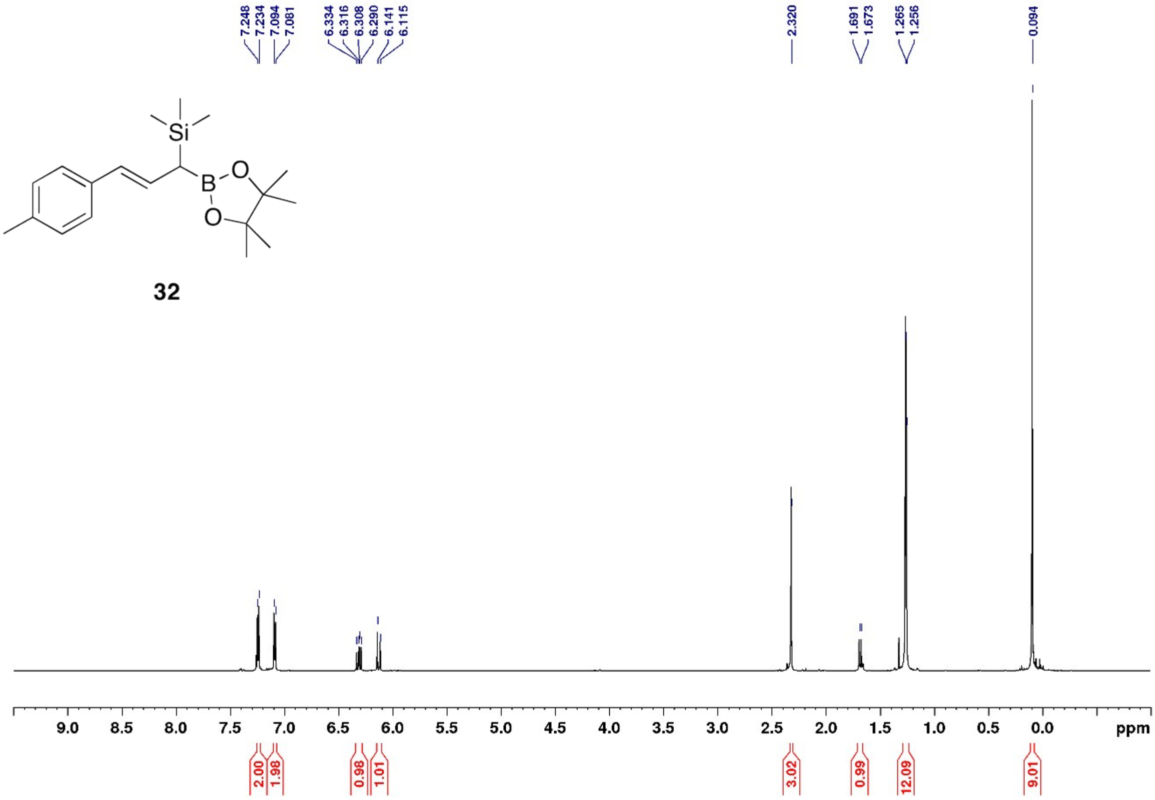

^{13}C NMR, 150 MHz, $CDCl_3$:

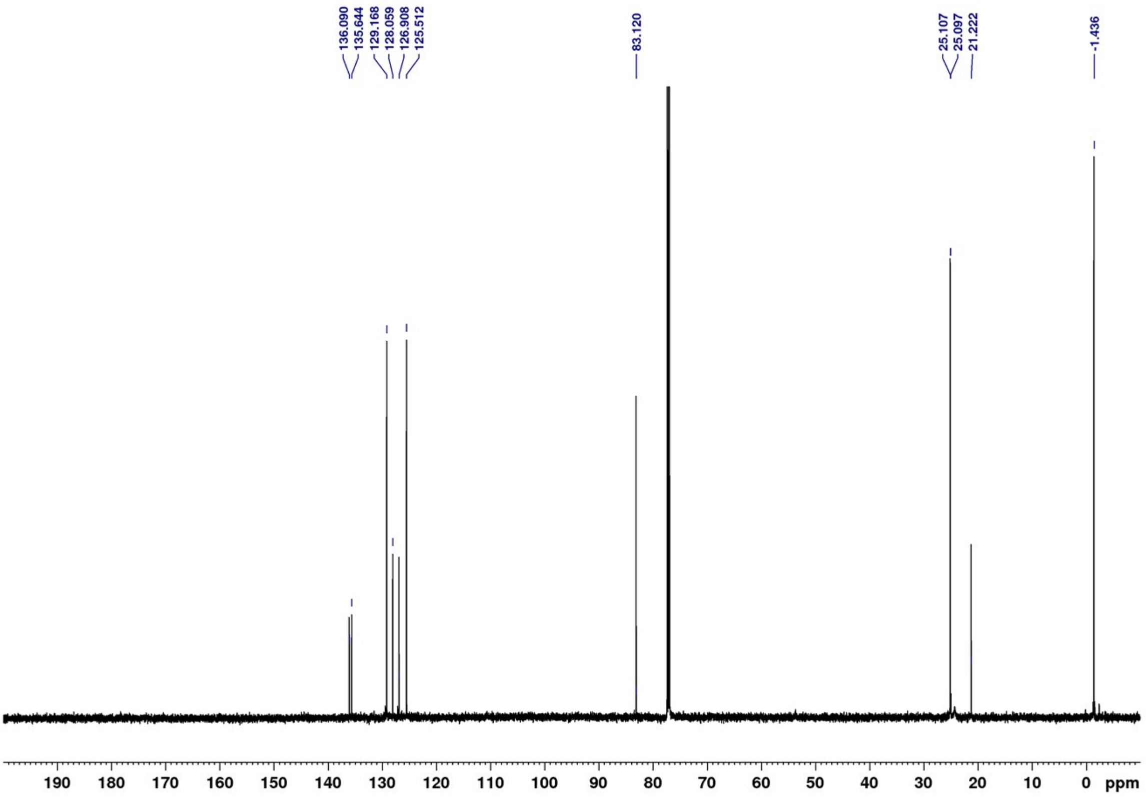

(*E*)-trimethyl(4-phenyl-1-(4,4,5,5-tetramethyl-1,3,2-dioxaborolan-2-yl)but-2-en-1-yl)silane (33):

^{1}H NMR, 600 MHz, $CDCl_3$:

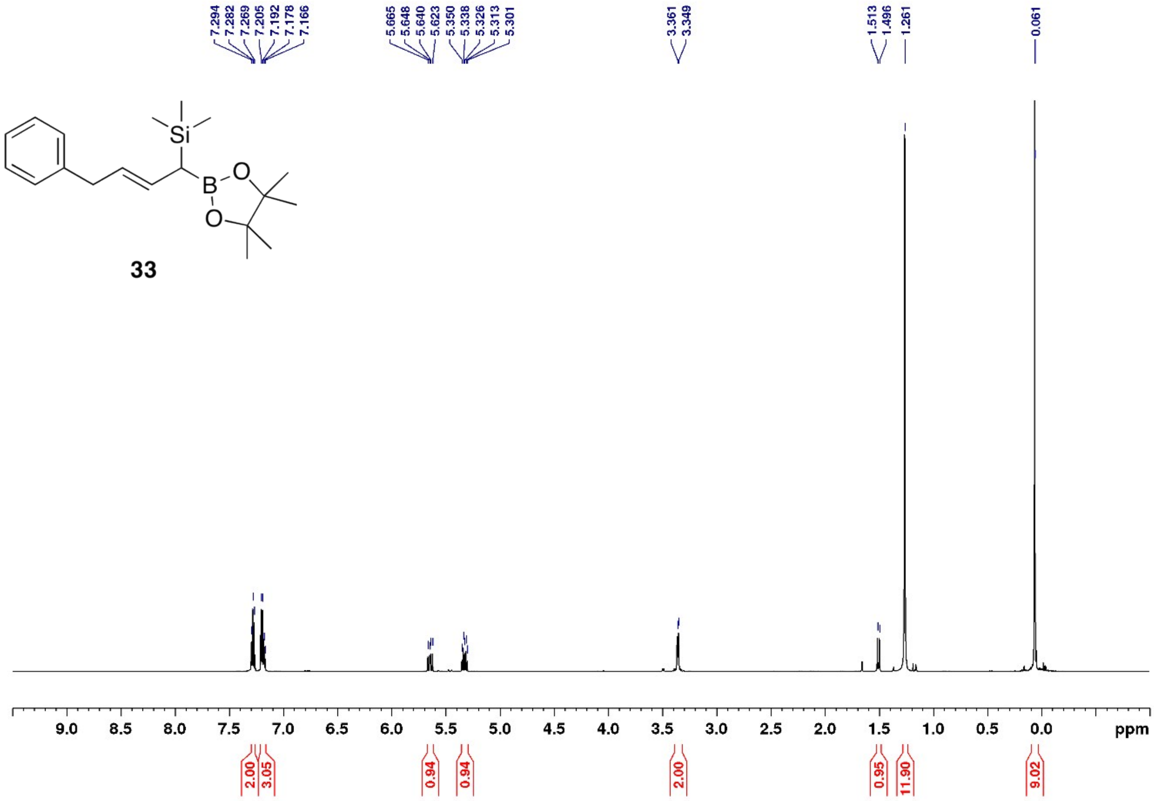

^{13}C NMR, 150 MHz, $CDCl_3$:

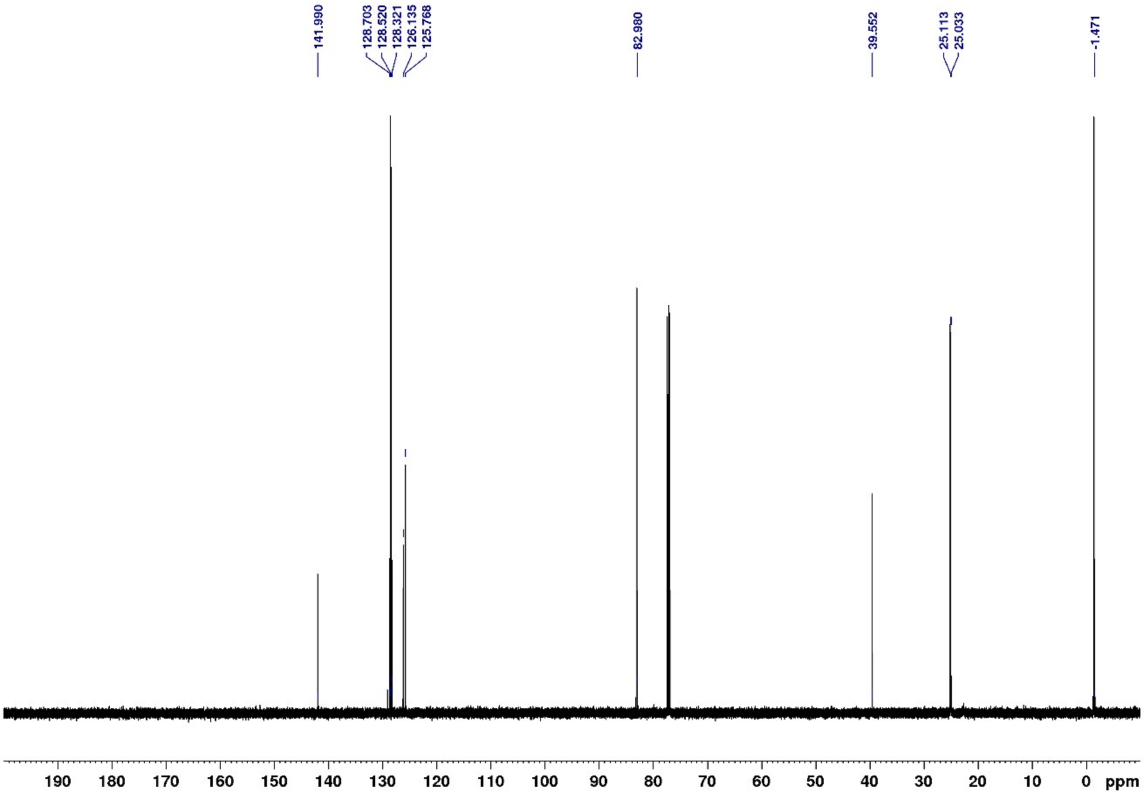

(*E*)-trimethyl(1-(4,4,5,5-tetramethyl-1,3,2-dioxaborolan-2-yl)hex-2-en-1-yl)silane (34):

^{1}H NMR, 600 MHz, $CDCl_3$:

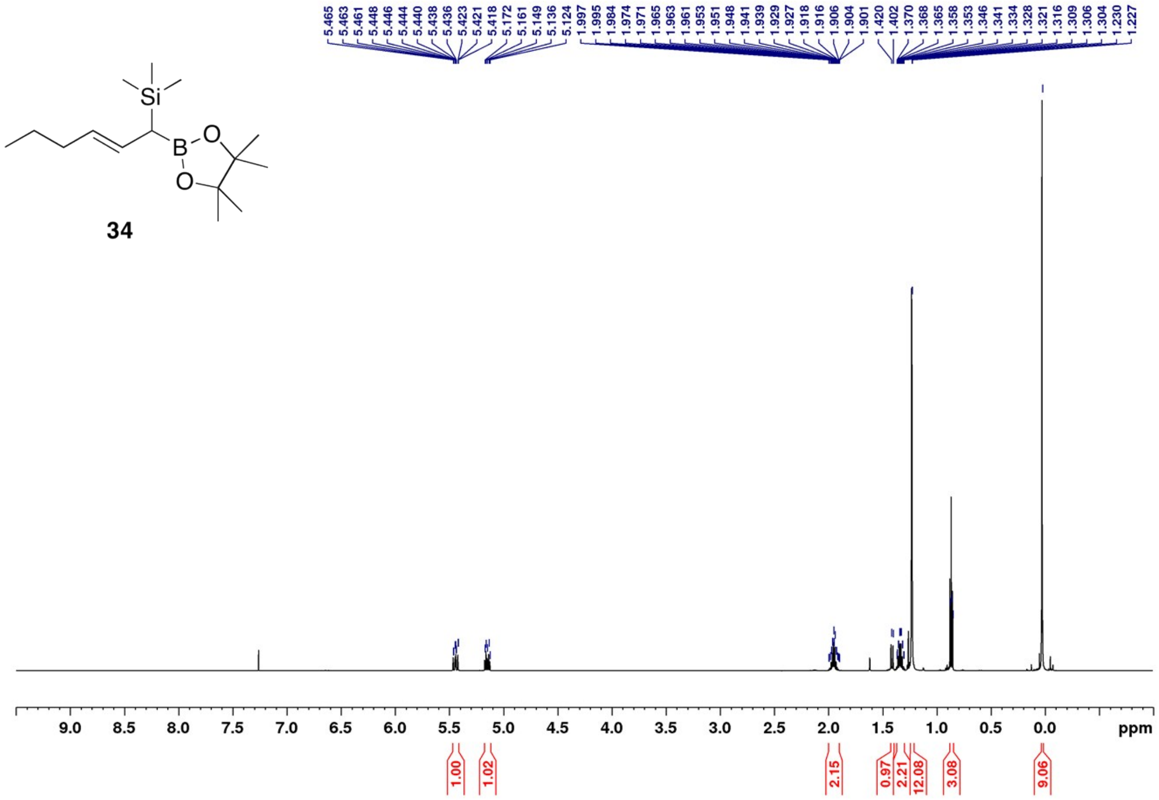

^{13}C NMR, 150 MHz, $CDCl_3$:

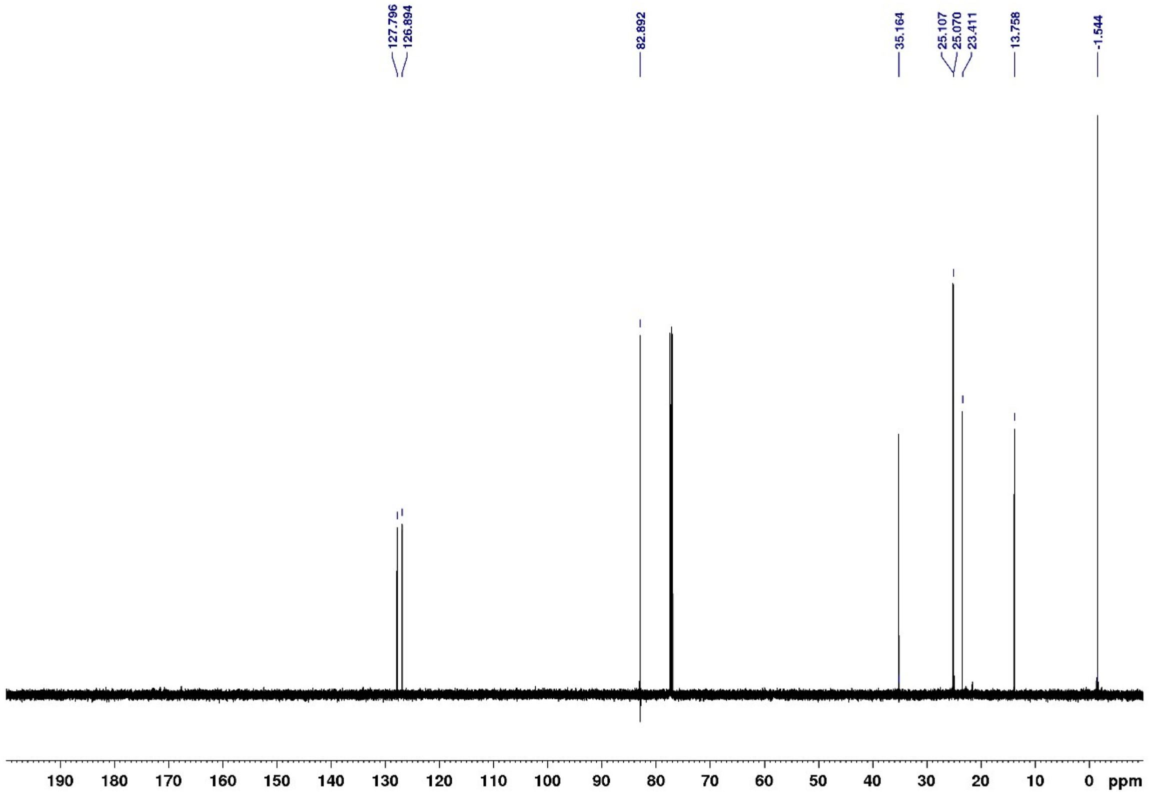

6. Computational methods

Geometries of all structures (minima and saddle points) were optimised at the ωB97xd/cc-PVDZ level ωB97xD/cc-PVTZ//ωB97xD/cc-PVDZ level calculations using the implicit Solvation Model based on Density (SMD) implemented in Gaussian 09 software in dichloromethane as a solvent (using the SMD solvation model, ε=8.93). Subsequent vibrational frequency calculations were performed at the same level for all calculated structures. When needed, multiple initial guesses (no more than four) were used to explore the conformational space fully. All transition states thus found possess exactly one negative Hessian eigenvalue, while all other stationary points were confirmed to be genuine minima on the potential energy surface (PES). Intrinsic reaction coordinate (IRC) analysis was performed to unambiguously assign located transition states when needed. Electronic energies were obtained by performing single-point calculations at the ωB97xd/cc-pVTZ level in solvent. Gibbs energies were calculated as $\Delta G = \Delta H - T\Delta S$ at 298 K where enthalpies and entropies were obtained by using standard statistical mechanical formulae for the ideal gas, rigid rotor, and harmonic oscillator approximations following the normal-mode analysis in vacuum. A correction of ($1.9\cdot\Delta n$) kcal mol^{-1} (corresponding to the difference between the concentration of the ideal gas at 298 K and 1 atm and its 1 mol l^{-1} concentration; Δn is the change in number of moles in the reaction) has been applied so that the computed values refer to 1 mol l^{-1} standard state at a given temperature.

Cartesian coordinates and uncorrected electronic energies of all computed structures optimised at ωB97xd/cc-PVDZ + SMD level of theory.

For all the computed pathways, the following identification was used:

Start – the initial boronic species
TS1 – coordination transition state
Intermediate – tetra-coordinated boron intermediate
TS2 – migratory insertion transition state
Product – the final product of the migratory insertion

For the reaction pathways involving the same initial boronic species, the same coordinates were used.

The computed reaction pathway for the coupling between 7 and TMS diazomethane 2

Start

C	-1.382711000	1.504711000	1.062234000
C	-2.370569000	-0.723629000	0.653853000
H	-0.552120000	1.971449000	1.610116000
H	-2.324834000	1.734161000	1.574278000
H	-2.744314000	-1.253014000	1.537843000
B	-0.293194000	-0.091120000	-0.465757000
N	-1.136222000	0.049749000	0.960050000
O	-0.853470000	-1.324894000	-1.028018000
O	-0.807096000	1.110432000	-1.173422000
C	-1.359980000	2.011279000	-0.372868000
O	-1.766487000	3.088065000	-0.730077000
C	-2.002976000	-1.682457000	-0.467085000
O	-2.666948000	-2.625583000	-0.812329000
H	-3.161350000	-0.053909000	0.286834000
C	1.291105000	-0.119840000	-0.303628000
C	1.988476000	-1.333127000	-0.195860000
C	2.036735000	1.067585000	-0.236305000
C	3.372056000	-1.363233000	-0.020186000
H	1.437898000	-2.275655000	-0.257501000
C	3.420501000	1.048332000	-0.057583000
H	1.532037000	2.033088000	-0.334552000
C	4.091963000	-0.169830000	0.051705000
H	3.892088000	-2.320849000	0.056960000
H	3.978081000	1.986496000	-0.010573000
H	5.175596000	-0.189328000	0.187407000
C	-0.452467000	-0.503797000	2.151226000
H	-0.255401000	-1.570632000	1.985358000
H	0.497297000	0.022387000	2.298079000
H	-1.096255000	-0.376948000	3.031934000

E(RwB97XD) = -806.864428604

TS1

C	-1.480839000	-0.889932000	-0.941276000
H	-1.439586000	-0.556855000	-1.986580000
N	-1.265711000	-2.203169000	-0.892412000
N	-0.990199000	-3.279560000	-0.734179000
C	0.229846000	1.274245000	-0.276814000
C	-0.022473000	1.992984000	0.901517000
C	0.270730000	1.993261000	-1.481318000
C	-0.226537000	3.373125000	0.882060000
H	-0.062354000	1.471877000	1.861536000
C	0.061568000	3.371657000	-1.511694000
H	0.470062000	1.462221000	-2.415860000
C	-0.189751000	4.067306000	-0.327775000
H	-0.419517000	3.907857000	1.814828000

H	0.095445000	3.906967000	-2.463380000
Si	-3.018526000	-0.331683000	0.063850000
C	-4.433405000	-1.483095000	-0.405341000
H	-5.352751000	-1.211718000	0.140514000
H	-4.649590000	-1.430273000	-1.485047000
H	-4.193535000	-2.530708000	-0.156485000
C	-2.661729000	-0.498172000	1.898304000
H	-3.597033000	-0.376571000	2.469815000
H	-2.246532000	-1.492297000	2.127234000
H	-1.943795000	0.256866000	2.249201000
C	-3.387427000	1.425375000	-0.479358000
H	-2.583372000	2.121924000	-0.197675000
H	-3.527846000	1.476751000	-1.571300000
H	-4.320794000	1.769582000	-0.003062000
H	-0.354951000	5.146670000	-0.348409000
C	3.216404000	-1.141559000	-0.280785000
C	2.501227000	-0.179894000	1.842065000
H	3.178708000	-2.039598000	0.357711000
H	4.252786000	-1.059433000	-0.645226000
H	3.299078000	-0.703021000	2.400595000
H	2.348886000	0.794720000	2.330942000
B	0.476367000	-0.286949000	-0.268486000
O	0.421833000	-1.056477000	0.951473000
O	1.093071000	-0.906797000	-1.419502000
C	1.211439000	-0.967583000	2.024143000
O	0.909281000	-1.497794000	3.065634000
C	2.320829000	-1.425659000	-1.478120000
O	2.677918000	-2.079652000	-2.427893000
N	2.778183000	0.029072000	0.445448000
C	3.494979000	1.244829000	0.109350000
H	4.546683000	1.232874000	0.456162000
H	3.491186000	1.380325000	-0.982682000
H	2.984467000	2.110176000	0.556276000

E(RwB97XD) = -1364.18409862

Intermediate

C	-1.459042000	-0.725861000	-0.899035000
H	-1.523723000	-0.431265000	-1.959115000
N	-1.556363000	-2.099487000	-0.893905000
N	-1.558926000	-3.202034000	-0.775073000
C	0.322198000	1.179961000	-0.299603000
C	0.169979000	1.929943000	0.876514000
C	0.575031000	1.888637000	-1.484610000
C	0.276841000	3.321465000	0.877379000
H	-0.037869000	1.422642000	1.822116000

C	0.680230000	3.280128000	-1.497051000
H	0.702813000	1.336604000	-2.420048000
C	0.530534000	4.003956000	-0.313229000
H	0.157525000	3.875978000	1.811295000
H	0.880306000	3.803606000	-2.435202000
Si	-2.999321000	0.024523000	0.053164000
C	-4.478076000	-0.996035000	-0.498384000
H	-5.397955000	-0.586648000	-0.048000000
H	-4.600662000	-0.975811000	-1.593548000
H	-4.393943000	-2.047686000	-0.177700000
C	-2.744127000	-0.146739000	1.899158000
H	-2.385133000	-1.154618000	2.158358000
H	-2.017830000	0.582154000	2.285797000
H	-3.704682000	0.020675000	2.414631000
C	-3.149309000	1.789754000	-0.554072000
H	-2.315605000	2.420606000	-0.212440000
H	-3.186248000	1.823696000	-1.654892000
H	-4.089066000	2.223441000	-0.171969000
H	0.611550000	5.093180000	-0.318319000
C	2.997558000	-1.454723000	-0.213527000
C	2.325734000	-0.420736000	1.885645000
H	2.745913000	-2.342220000	0.392013000
H	4.056118000	-1.565477000	-0.502004000
H	3.026696000	-1.010276000	2.511062000
H	2.233600000	0.567200000	2.364239000
B	0.164945000	-0.416074000	-0.341847000
O	0.178288000	-1.154794000	0.943310000
O	0.923336000	-1.083493000	-1.438297000
C	0.960910000	-1.086679000	2.013396000
O	0.598998000	-1.562666000	3.068144000
C	2.162249000	-1.552130000	-1.482905000
O	2.589967000	-2.072551000	-2.491993000
N	2.737348000	-0.243916000	0.524188000
C	3.664254000	0.835175000	0.271369000
H	4.674735000	0.658103000	0.696728000
H	3.772752000	0.981530000	-0.814393000
H	3.267583000	1.771023000	0.694283000

E(RwB97XD) = -1364.18980819

TS2

C	-1.355119000	-0.493921000	-0.833833000
H	-1.434331000	-0.325937000	-1.921014000
N	-1.637404000	-2.395347000	-1.003919000
N	-1.462046000	-3.461066000	-0.797956000
C	0.263701000	1.150122000	-0.356419000

C	0.061099000	1.939891000	0.788088000
C	0.546067000	1.811291000	-1.563349000
C	0.186350000	3.327197000	0.743174000
H	-0.189204000	1.464768000	1.739387000
C	0.662971000	3.200443000	-1.618791000
H	0.699243000	1.223515000	-2.472238000
C	0.486015000	3.962771000	-0.463720000
H	0.041146000	3.916792000	1.651207000
H	0.892287000	3.691139000	-2.567453000
Si	-2.981211000	0.041403000	0.082323000
C	-4.362300000	-1.043706000	-0.592221000
H	-5.331877000	-0.653893000	-0.238956000
H	-4.380191000	-1.042736000	-1.694247000
H	-4.277069000	-2.085780000	-0.244318000
C	-2.803456000	-0.196465000	1.931386000
H	-2.125175000	0.543799000	2.380847000
H	-3.789468000	-0.091200000	2.413905000
H	-2.409862000	-1.198692000	2.160148000
C	-3.276698000	1.823774000	-0.432067000
H	-2.516248000	2.505934000	-0.024040000
H	-3.276743000	1.919060000	-1.530063000
H	-4.265086000	2.148561000	-0.064865000
H	0.577541000	5.050319000	-0.503559000
C	3.035859000	-1.388251000	-0.122621000
C	2.317079000	-0.233884000	1.897674000
H	2.803437000	-2.240037000	0.539070000
H	4.099629000	-1.487196000	-0.396099000
H	3.043928000	-0.727794000	2.574539000
H	2.159137000	0.781712000	2.295290000
B	0.171034000	-0.478478000	-0.317907000
O	0.206800000	-1.135714000	1.011343000
O	0.957592000	-1.168145000	-1.377472000
C	0.993054000	-0.970091000	2.066263000
O	0.666451000	-1.409184000	3.149104000
C	2.212908000	-1.589110000	-1.387653000
O	2.669208000	-2.147394000	-2.364351000
N	2.734354000	-0.138811000	0.530448000
C	3.624224000	0.950923000	0.205932000
H	4.638159000	0.840916000	0.646077000
H	3.734491000	1.023704000	-0.887189000
H	3.192781000	1.899949000	0.560526000

E(RwB97XD) = -1364.16494101

<u>The computed reaction pathway for the coupling between 8 and TMS diazomethane 2</u>

Start

C	1.676481000	0.000041000	-0.000048000
C	2.396519000	1.191472000	0.182215000
C	2.396475000	-1.191433000	-0.182262000
C	3.790658000	1.194749000	0.184838000
H	1.853853000	2.129531000	0.324234000
C	3.790607000	-1.194775000	-0.184810000
H	1.853791000	-2.129478000	-0.324316000
C	4.488883000	-0.000024000	0.000029000
H	4.336206000	2.129776000	0.329748000
H	4.336128000	-2.129823000	-0.329686000
H	5.581291000	-0.000060000	0.000057000
C	-2.014226000	-0.778377000	0.102786000
C	-2.014326000	0.778341000	-0.102811000
O	-0.636242000	-1.129050000	-0.191100000
O	-0.636260000	1.129121000	0.191013000
C	-2.275354000	-1.194225000	1.549191000
H	-3.324302000	-1.024530000	1.832739000
H	-2.058587000	-2.267106000	1.657914000
H	-1.629701000	-0.643511000	2.249559000
C	-2.927093000	-1.545183000	-0.839894000
H	-2.866904000	-2.622123000	-0.623070000
H	-3.972653000	-1.229324000	-0.703225000
H	-2.649287000	-1.392609000	-1.890962000
C	-2.275529000	1.194285000	-1.549174000
H	-3.324594000	1.025016000	-1.832530000
H	-2.058349000	2.267079000	-1.657910000
H	-1.630218000	0.643307000	-2.249654000
C	-2.927123000	1.545043000	0.840008000
H	-2.867155000	2.621991000	0.623154000
H	-3.972674000	1.229038000	0.703593000
H	-2.649074000	1.392493000	1.891020000
B	0.117282000	0.000059000	-0.000073000

E(RwB97XD) = -642.792588286

TS1

C	-0.748427000	-1.104540000	-0.960303000
H	-1.410288000	-0.625440000	-1.696765000
N	0.172549000	-1.789201000	-1.647080000
N	1.042869000	-2.287102000	-2.146290000
C	-0.921386000	1.544286000	-0.158960000
C	-1.379941000	2.054010000	1.064555000
C	-1.500612000	2.058187000	-1.330178000
C	-2.390842000	3.015812000	1.123788000
H	-0.931180000	1.683455000	1.989852000

C	-2.507533000	3.022229000	-1.284974000
H	-1.153397000	1.697220000	-2.303643000
C	-2.959976000	3.502077000	-0.053562000
H	-2.733609000	3.391073000	2.091335000
H	-2.939899000	3.406777000	-2.212062000
Si	-1.707044000	-2.189406000	0.309472000
C	-2.562002000	-3.555865000	-0.662991000
H	-3.131236000	-4.213114000	0.015825000
H	-3.267067000	-3.141762000	-1.402576000
H	-1.829091000	-4.179852000	-1.201178000
C	-0.495778000	-2.914863000	1.539415000
H	0.270462000	-3.529899000	1.040557000
H	0.005698000	-2.105846000	2.089717000
H	-1.031831000	-3.559629000	2.255683000
C	-2.968582000	-1.077565000	1.136193000
H	-2.489382000	-0.343310000	1.799900000
H	-3.579744000	-0.532569000	0.399083000
H	-3.645732000	-1.699289000	1.746378000
H	-3.749332000	4.256126000	-0.013793000
C	2.545106000	0.795027000	-0.434820000
C	2.268078000	0.035901000	0.917500000
O	0.843782000	0.133761000	1.054346000
O	1.316244000	0.640263000	-1.154681000
B	0.273596000	0.476243000	-0.198889000
C	2.905814000	0.685926000	2.141900000
H	2.668461000	0.096085000	3.040680000
H	4.001832000	0.723768000	2.042232000
H	2.531597000	1.705956000	2.299689000
C	2.674079000	-1.438818000	0.880210000
H	3.768044000	-1.550279000	0.839835000
H	2.318065000	-1.936833000	1.793544000
H	2.247587000	-1.965084000	0.016633000
C	2.772687000	2.297003000	-0.237436000
H	3.736906000	2.508610000	0.248448000
H	2.768292000	2.787078000	-1.222569000
H	1.969708000	2.748287000	0.364500000
C	3.677225000	0.210938000	-1.269370000
H	3.798463000	0.798536000	-2.192257000
H	4.630889000	0.242700000	-0.719788000
H	3.470839000	-0.828835000	-1.556972000

E(RwB97XD) = -1200.13201270

Intermediate

C	-0.814072000	-1.040982000	-0.944321000
H	-1.491610000	-0.602813000	-1.694709000

N	0.025649000	-1.849761000	-1.640331000
N	0.826020000	-2.446120000	-2.133877000
C	-0.815181000	1.550541000	-0.190335000
C	-1.089780000	2.178375000	1.033761000
C	-1.416279000	2.095562000	-1.336276000
C	-1.936724000	3.285745000	1.118451000
H	-0.621375000	1.783552000	1.939210000
C	-2.262034000	3.203596000	-1.268098000
H	-1.214980000	1.646820000	-2.314910000
C	-2.528162000	3.802641000	-0.034978000
H	-2.133881000	3.751054000	2.087748000
H	-2.712529000	3.605991000	-2.179080000
Si	-1.829660000	-2.073418000	0.352184000
C	-2.948985000	-3.202075000	-0.651648000
H	-3.566824000	-3.827297000	0.014673000
H	-3.628025000	-2.619983000	-1.295900000
H	-2.360808000	-3.877367000	-1.295168000
C	-0.636855000	-3.070848000	1.393048000
H	0.013322000	-3.708083000	0.771532000
H	-0.006614000	-2.397332000	1.990711000
H	-1.201867000	-3.730905000	2.072237000
C	-2.830043000	-0.861225000	1.366083000
H	-2.184084000	-0.240755000	2.003753000
H	-3.435886000	-0.197051000	0.729701000
H	-3.516607000	-1.428942000	2.016951000
H	-3.191029000	4.669009000	0.025101000
C	2.503529000	0.657809000	-0.441382000
C	2.212747000	-0.132109000	0.889164000
O	0.790344000	-0.064315000	1.002593000
O	1.318675000	0.443910000	-1.204706000
B	0.218468000	0.303146000	-0.272359000
C	2.825600000	0.496569000	2.137943000
H	2.582066000	-0.115920000	3.020034000
H	3.922944000	0.550257000	2.058064000
H	2.437400000	1.508642000	2.313322000
C	2.648102000	-1.599272000	0.818447000
H	3.744640000	-1.695277000	0.813685000
H	2.267757000	-2.134759000	1.701035000
H	2.260997000	-2.102608000	-0.077897000
C	2.665404000	2.165727000	-0.211941000
H	3.615923000	2.408918000	0.287039000
H	2.649698000	2.673431000	-1.188394000
H	1.840370000	2.574344000	0.389611000
C	3.693790000	0.138343000	-1.239270000
H	3.836634000	0.758158000	-2.138080000
H	4.621341000	0.183177000	-0.647017000
H	3.535748000	-0.898120000	-1.567557000

E(RwB97XD) = -1200.13267963

TS2

C	-0.752314000	-0.939918000	-0.795685000
H	-0.977114000	-0.825208000	-1.868999000
N	0.081990000	-2.572876000	-0.937470000
N	0.813105000	-3.276846000	-0.506095000
C	-0.617523000	1.321453000	-0.255729000
C	-1.031597000	1.824502000	0.988214000
C	-1.076857000	1.972665000	-1.410837000
C	-1.857933000	2.943778000	1.078253000
H	-0.673743000	1.334704000	1.896616000
C	-1.917083000	3.084852000	-1.331804000
H	-0.761793000	1.609500000	-2.393409000
C	-2.311402000	3.571733000	-0.084894000
H	-2.153722000	3.329460000	2.057014000
H	-2.264543000	3.575139000	-2.244313000
Si	-2.337221000	-1.471524000	0.195556000
C	-2.843405000	-3.175422000	-0.430622000
H	-3.828299000	-3.432135000	-0.005168000
H	-2.936522000	-3.197316000	-1.528722000
H	-2.136552000	-3.962946000	-0.124537000
C	-1.924097000	-1.568324000	2.021821000
H	-0.988251000	-2.129362000	2.171976000
H	-1.799264000	-0.572109000	2.470713000
H	-2.731546000	-2.090521000	2.561717000
C	-3.704813000	-0.251029000	-0.213907000
H	-3.487299000	0.762353000	0.154435000
H	-3.869964000	-0.200152000	-1.302322000
H	-4.643414000	-0.594781000	0.253509000
H	-2.968447000	4.441915000	-0.018455000
C	2.775355000	0.151667000	-0.627372000
C	2.403151000	0.167022000	0.908093000
O	1.068190000	-0.330825000	0.929183000
O	1.515867000	0.248090000	-1.286116000
B	0.446292000	0.035412000	-0.324963000
C	3.266974000	-0.745879000	1.772475000
H	4.329813000	-0.462358000	1.716712000
H	2.951899000	-0.667606000	2.824804000
H	3.167539000	-1.797253000	1.469605000
C	2.418429000	1.579114000	1.507861000
H	1.983608000	1.539745000	2.518532000
H	3.440580000	1.978347000	1.593188000
H	1.820547000	2.282354000	0.911280000
C	3.454866000	-1.148450000	-1.071395000

H	3.490131000	-1.172505000	-2.171571000
H	4.486419000	-1.225090000	-0.694296000
H	2.903677000	-2.035399000	-0.733170000
C	3.648164000	1.325927000	-1.068788000
H	4.608997000	1.337538000	-0.529827000
H	3.866183000	1.238104000	-2.144735000
H	3.143081000	2.287652000	-0.907219000

E(RwB97XD) = -1200.10421469

The computed reaction pathway for the coupling between 9 and TMS diazomethane 2

Start

B	-1.752640000	-0.000602000	0.000074000
C	-0.176801000	0.010665000	0.000181000
C	0.537275000	-1.199474000	0.000092000
C	0.561478000	1.204910000	0.000182000
C	1.931128000	-1.217476000	-0.000134000
H	-0.012708000	-2.143430000	0.000158000
C	1.955964000	1.197300000	0.000083000
H	0.048367000	2.171549000	0.000168000
C	2.643436000	-0.016889000	-0.000138000
H	2.465755000	-2.169888000	-0.000270000
H	2.507965000	2.139547000	0.000062000
H	3.735677000	-0.027347000	-0.000324000
O	-2.530066000	1.126791000	-0.000243000
H	-2.023697000	1.948190000	-0.000191000
O	-2.385687000	-1.212013000	0.000046000
H	-3.347023000	-1.108054000	-0.000001000

E(RwB97XD) = -408.177727942

TS1

C	1.178397000	0.478696000	-0.676448000
H	0.925440000	0.503317000	-1.746863000
B	-0.073707000	1.576060000	0.188857000
O	-0.015242000	2.748332000	-0.644159000
H	-0.889023000	3.151404000	-0.688680000
O	0.444582000	1.832672000	1.501145000
N	2.306813000	1.208429000	-0.516857000
N	3.174448000	1.852716000	-0.241800000
H	0.282341000	1.095475000	2.098543000
C	-1.442329000	0.716663000	0.083727000
C	-2.068467000	0.143634000	1.200830000
C	-2.053834000	0.509075000	-1.164939000
C	-3.231760000	-0.620659000	1.082607000

H	-1.645701000	0.296345000	2.198819000
C	-3.215757000	-0.250342000	-1.299012000
H	-1.602775000	0.951290000	-2.058809000
C	-3.806975000	-0.825271000	-0.171749000
H	-3.693058000	-1.055536000	1.972687000
H	-3.665201000	-0.396891000	-2.284431000
Si	1.431646000	-1.330131000	-0.027931000
C	3.108575000	-1.904857000	-0.656776000
H	3.311165000	-2.935187000	-0.319848000
H	3.148705000	-1.893226000	-1.758231000
H	3.920717000	-1.262528000	-0.276832000
C	1.418903000	-1.316432000	1.849093000
H	2.112048000	-0.561183000	2.253353000
H	0.409557000	-1.125854000	2.246125000
H	1.742137000	-2.301717000	2.224831000
C	0.044651000	-2.364193000	-0.747814000
H	-0.930455000	-2.099132000	-0.312226000
H	-0.016745000	-2.248394000	-1.841658000
H	0.240688000	-3.428229000	-0.532033000
H	-4.716320000	-1.422348000	-0.270397000

E(RwB97XD) = -965.513515257

Intermediate

C	1.174257000	0.481233000	-0.662072000
H	0.947801000	0.494624000	-1.739654000
B	-0.057817000	1.570969000	0.147356000
O	-0.140202000	2.772763000	-0.641793000
H	0.559407000	3.380658000	-0.373073000
O	0.513644000	1.735493000	1.466982000
N	2.333170000	1.170074000	-0.500083000
N	3.221035000	1.790059000	-0.237130000
H	-0.071561000	2.290984000	1.995278000
C	-1.432802000	0.726165000	0.047577000
C	-2.000404000	0.128824000	1.182542000
C	-2.101887000	0.544181000	-1.174293000
C	-3.173950000	-0.625843000	1.108061000
H	-1.505379000	0.256076000	2.149543000
C	-3.270345000	-0.212348000	-1.265204000
H	-1.695037000	1.003533000	-2.080383000
C	-3.810146000	-0.804701000	-0.120625000
H	-3.592696000	-1.078272000	2.010767000
H	-3.766340000	-0.342140000	-2.230565000
Si	1.397359000	-1.344275000	-0.022255000
C	3.052076000	-1.936700000	-0.696113000
H	3.237575000	-2.978086000	-0.383491000

H	3.071329000	-1.906049000	-1.797989000
H	3.887921000	-1.322977000	-0.320212000
C	1.417827000	-1.355384000	1.849384000
H	2.150720000	-0.634298000	2.243117000
H	0.430818000	-1.096517000	2.257848000
H	1.695382000	-2.360704000	2.208541000
C	-0.005430000	-2.348075000	-0.753713000
H	-0.978092000	-2.076640000	-0.317398000
H	-0.061855000	-2.221286000	-1.846681000
H	0.178716000	-3.416446000	-0.548096000
H	-4.724787000	-1.398543000	-0.187047000

E(RwB97XD) = -965.513272024

TS2

C	0.896005000	0.403708000	-0.588797000
H	0.764760000	0.455943000	-1.682755000
B	0.089260000	1.578578000	0.194806000
O	-0.016993000	2.837554000	-0.524683000
H	0.751812000	3.379540000	-0.310552000
O	0.587223000	1.685660000	1.554544000
N	2.625123000	1.107126000	-0.624850000
N	3.396445000	1.784567000	-0.225251000
H	0.033675000	2.311934000	2.035283000
C	-1.341184000	0.751298000	0.039880000
C	-1.910848000	0.120117000	1.158147000
C	-2.042189000	0.678261000	-1.175306000
C	-3.136200000	-0.540776000	1.075470000
H	-1.380708000	0.165306000	2.112820000
C	-3.263456000	0.008951000	-1.271736000
H	-1.629148000	1.164595000	-2.063366000
C	-3.813227000	-0.603998000	-0.144781000
H	-3.566711000	-1.010502000	1.963370000
H	-3.790320000	-0.034400000	-2.228103000
Si	1.235845000	-1.421091000	-0.027079000
C	2.874861000	-1.942959000	-0.796821000
H	3.036478000	-3.019740000	-0.619831000
H	2.884041000	-1.774704000	-1.886237000
H	3.728433000	-1.402437000	-0.356225000
C	1.336894000	-1.534999000	1.840621000
H	2.019848000	-0.771559000	2.243691000
H	0.351418000	-1.389779000	2.306986000
H	1.713370000	-2.529548000	2.133472000
C	-0.152518000	-2.466158000	-0.743373000
H	-1.128493000	-2.207889000	-0.304900000
H	-0.217705000	-2.343424000	-1.836578000

H	0.048348000	-3.530763000	-0.533869000
H	-4.768875000	-1.128425000	-0.216117000

E(RwB97XD) = -965.486754677

Product

C	0.419475000	0.363302000	-0.682355000
H	0.578695000	0.129825000	-1.751038000
B	1.141234000	1.730401000	-0.370023000
O	2.230442000	2.168788000	-1.087008000
O	0.740488000	2.510663000	0.681819000
C	-1.055153000	0.244706000	-0.382185000
C	-1.918170000	-0.331887000	-1.327929000
C	-1.609558000	0.647022000	0.844231000
C	-3.277839000	-0.497932000	-1.066371000
H	-1.512856000	-0.658386000	-2.289612000
C	-2.968937000	0.481005000	1.108096000
H	-0.967050000	1.106739000	1.595373000
C	-3.812656000	-0.092685000	0.156542000
H	-3.922508000	-0.948198000	-1.824862000
H	-3.371814000	0.806043000	2.070297000
Si	1.387768000	-1.045452000	0.201010000
C	0.581508000	-2.694683000	-0.219082000
H	0.556869000	-2.864282000	-1.308470000
H	1.146301000	-3.524357000	0.238775000
H	-0.453024000	-2.742126000	0.157583000
C	3.164974000	-1.024173000	-0.432689000
H	3.202494000	-1.176842000	-1.524552000
H	3.666844000	-0.070586000	-0.201850000
H	3.749093000	-1.833568000	0.036529000
C	1.377430000	-0.782741000	2.066189000
H	1.770951000	0.212030000	2.331487000
H	0.360338000	-0.870041000	2.480742000
H	2.007364000	-1.541543000	2.560365000
H	-4.876558000	-0.221527000	0.365852000
H	2.460991000	1.575800000	-1.812820000
H	1.295053000	3.298129000	0.767008000

E(RwB97XD) = -856.129912922

<u>The computed reaction pathway for the coupling between 9 and diazomethane</u>

TS1

C	-1.595619000	-0.411033000	-1.058181000
H	-1.318431000	-1.461092000	-1.212669000
B	-0.871343000	0.255263000	0.482483000

O	-1.400820000	-0.614480000	1.498411000
H	-0.699007000	-0.884696000	2.099081000
O	-1.394201000	1.587015000	0.623322000
N	-2.945439000	-0.358826000	-0.812238000
N	-3.984002000	-0.237496000	-0.439291000
H	-0.990472000	2.195157000	-0.005342000
C	0.718759000	0.105482000	0.187523000
C	1.540513000	1.204460000	-0.107226000
C	1.334142000	-1.158460000	0.219594000
C	2.906327000	1.056194000	-0.359044000
H	1.112986000	2.211882000	-0.133668000
C	2.697475000	-1.321747000	-0.028211000
H	0.728698000	-2.041643000	0.448742000
C	3.490193000	-0.210319000	-0.321527000
H	3.518894000	1.933229000	-0.582453000
H	3.145434000	-2.317981000	0.006883000
H	4.558031000	-0.331006000	-0.517641000
H	-1.383905000	0.226349000	-1.926081000

E(RwB97XD) = -556.859285721

Intermediate

C	-1.567903000	0.119317000	-1.111206000
H	-1.337894000	-0.730027000	-1.766812000
B	-0.865184000	-0.106324000	0.520241000
O	-1.256423000	-1.407065000	1.001291000
H	-2.086286000	-1.328213000	1.486151000
O	-1.425959000	1.019081000	1.242209000
N	-2.932917000	0.134075000	-0.913284000
N	-3.986622000	0.113534000	-0.567476000
H	-0.950841000	1.126337000	2.074750000
C	0.723459000	-0.042652000	0.207858000
C	1.402654000	1.185061000	0.152102000
C	1.475601000	-1.204248000	-0.031396000
C	2.769207000	1.256118000	-0.127282000
H	0.844527000	2.107877000	0.339172000
C	2.841129000	-1.146990000	-0.315855000
H	0.976789000	-2.176318000	0.015809000
C	3.493451000	0.086782000	-0.365255000
H	3.272693000	2.225862000	-0.159403000
H	3.402339000	-2.067554000	-0.496361000
H	4.562328000	0.136229000	-0.586179000
H	-1.287489000	1.087710000	-1.544803000

E(RwB97XD) = -556.860938233

TS2

C	-1.264249000	0.050273000	-0.984840000
H	-1.113653000	-0.813656000	-1.645126000
B	-0.907587000	-0.082857000	0.583888000
O	-1.201948000	-1.369170000	1.179022000
H	-2.105033000	-1.352851000	1.517182000
O	-1.403337000	1.080430000	1.288737000
N	-3.072214000	0.101475000	-1.114872000
N	-4.072252000	0.109676000	-0.653095000
H	-1.025603000	1.088188000	2.176025000
C	0.704637000	-0.026889000	0.173289000
C	1.387277000	1.198521000	0.106678000
C	1.437328000	-1.200535000	-0.069392000
C	2.756994000	1.251812000	-0.154180000
H	0.831079000	2.123645000	0.283141000
C	2.806780000	-1.157182000	-0.331727000
H	0.922844000	-2.163997000	-0.029636000
C	3.469642000	0.071403000	-0.374293000
H	3.271993000	2.214990000	-0.187943000
H	3.361652000	-2.082676000	-0.504045000
H	4.541638000	0.108822000	-0.581407000
H	-1.083891000	0.999262000	-1.507144000

E(RwB97XD) = -556.841795438

<u>The computed reaction pathway for the coupling between 10 and TMS diazomethane 2</u>

Start

B	0.009937000	1.075357000	-0.030336000
C	-1.382366000	0.334867000	-0.038605000
C	-1.562106000	-0.892846000	-0.699703000
C	-2.503404000	0.907684000	0.589200000
C	-2.807487000	-1.519582000	-0.738699000
H	-0.713910000	-1.362599000	-1.204373000
C	-3.747550000	0.278752000	0.570247000
H	-2.403452000	1.860122000	1.119091000
C	-3.901861000	-0.936297000	-0.098843000
H	-2.925180000	-2.467558000	-1.268130000
H	-4.600078000	0.737553000	1.075649000
H	-4.876173000	-1.429412000	-0.120790000
C	1.394911000	0.332209000	0.033776000
C	2.558423000	0.957576000	-0.450676000
C	1.526065000	-0.955120000	0.582924000
C	3.797031000	0.320702000	-0.404189000
H	2.487108000	1.961605000	-0.876366000

C	2.764891000	-1.593395000	0.647442000
H	0.644725000	-1.465480000	0.979784000
C	3.901749000	-0.958114000	0.147114000
H	4.685336000	0.821401000	-0.795681000
H	2.843914000	-2.590213000	1.086750000
H	4.871777000	-1.458675000	0.188665000
O	0.064303000	2.439085000	-0.085971000
H	-0.807958000	2.845172000	-0.185074000

E(RwB97XD) = -563.914450431

TS1

C	-0.383417000	-1.154106000	-0.707549000
H	-0.300759000	-0.664531000	-1.686383000
N	0.503741000	-2.145025000	-0.657153000
N	1.280864000	-2.938502000	-0.488214000
C	-0.449216000	1.583674000	0.248093000
C	-0.949642000	2.398181000	1.279065000
C	-0.708874000	2.002498000	-1.067933000
C	-1.672489000	3.562632000	1.015971000
H	-0.774537000	2.102912000	2.316322000
C	-1.435265000	3.161634000	-1.345150000
H	-0.345609000	1.411189000	-1.912763000
C	-1.921690000	3.948987000	-0.300973000
H	-2.046401000	4.170324000	1.843695000
H	-1.622732000	3.449937000	-2.382240000
Si	-2.132305000	-1.750794000	-0.159787000
C	-2.957878000	-2.485625000	-1.683951000
H	-3.955637000	-2.881218000	-1.429096000
H	-3.087908000	-1.724289000	-2.470920000
H	-2.361431000	-3.312618000	-2.102838000
C	-1.858808000	-3.066466000	1.153459000
H	-1.302046000	-3.933852000	0.762542000
H	-1.303822000	-2.646051000	2.006504000
H	-2.830350000	-3.433464000	1.524101000
C	-3.152731000	-0.325853000	0.499868000
H	-2.718139000	0.079878000	1.424363000
H	-3.246065000	0.490859000	-0.232561000
H	-4.166671000	-0.700649000	0.721928000
H	-2.489783000	4.857638000	-0.512883000
B	0.407023000	0.290685000	0.669910000
C	1.964299000	0.225020000	0.276592000
C	2.847484000	-0.644322000	0.942692000
C	2.518255000	1.023382000	-0.740722000
C	4.202940000	-0.719882000	0.616297000
H	2.483637000	-1.292558000	1.746677000

C	3.869273000	0.956593000	-1.079069000
H	1.881071000	1.728786000	-1.278350000
C	4.719210000	0.081576000	-0.400392000
H	4.856092000	-1.406691000	1.159330000
H	4.263375000	1.597673000	-1.871175000
H	5.779062000	0.028501000	-0.659122000
O	0.017546000	-0.221522000	1.923014000
H	0.684496000	-0.794754000	2.316021000

E(RwB97XD) = -1121.26098024

Intermediate

C	-0.500704000	-1.020447000	-0.637845000
H	-0.460863000	-0.638641000	-1.670186000
N	0.224382000	-2.183943000	-0.659170000
N	0.850027000	-3.096001000	-0.557929000
C	-0.290247000	1.541116000	0.179918000
C	-0.603431000	2.370009000	1.270398000
C	-0.515115000	2.081409000	-1.098166000
C	-1.109541000	3.659748000	1.103467000
H	-0.462518000	1.984813000	2.284088000
C	-1.025474000	3.368654000	-1.282960000
H	-0.294868000	1.486263000	-1.990505000
C	-1.326906000	4.166142000	-0.178707000
H	-1.342653000	4.271531000	1.978881000
H	-1.191019000	3.748778000	-2.294266000
Si	-2.342600000	-1.480917000	-0.130737000
C	-3.143108000	-2.115979000	-1.708113000
H	-4.182222000	-2.428408000	-1.510513000
H	-3.166406000	-1.334715000	-2.485286000
H	-2.599612000	-2.986693000	-2.110826000
C	-2.243083000	-2.828535000	1.168061000
H	-1.762051000	-3.743942000	0.786183000
H	-1.673651000	-2.464727000	2.036389000
H	-3.259606000	-3.101394000	1.497297000
C	-3.214662000	0.044214000	0.505509000
H	-2.749735000	0.396478000	1.437049000
H	-3.205307000	0.865361000	-0.227157000
H	-4.265860000	-0.219679000	0.712922000
H	-1.728474000	5.172735000	-0.316074000
B	0.327981000	0.058308000	0.473476000
C	1.934721000	-0.033854000	0.182443000
C	2.731350000	-0.934831000	0.913686000
C	2.595090000	0.728400000	-0.797769000
C	4.102930000	-1.071453000	0.686449000
H	2.262536000	-1.548717000	1.688810000

C	3.963453000	0.599924000	-1.039509000
H	2.028232000	1.454783000	-1.386272000
C	4.726007000	-0.303227000	-0.296206000
H	4.686487000	-1.780830000	1.278896000
H	4.440786000	1.212927000	-1.808329000
H	5.798269000	-0.403248000	-0.479912000
O	-0.038169000	-0.483443000	1.785029000
H	0.581833000	-0.181968000	2.457282000

E(RwB97XD) = -1121.26267578

TS2

C	-0.497735000	-0.892775000	-0.405465000
H	-0.280760000	-0.776891000	-1.480801000
B	0.483802000	-0.062023000	0.589217000
N	0.172765000	-2.618893000	-0.314777000
N	0.704004000	-3.452043000	0.171315000
C	-0.247029000	1.368627000	0.179893000
C	-1.045995000	2.020940000	1.134200000
C	-0.107321000	1.979937000	-1.076921000
C	-1.666259000	3.237963000	0.853418000
H	-1.162324000	1.563042000	2.118579000
C	-0.733723000	3.192241000	-1.371053000
H	0.516658000	1.506775000	-1.841285000
C	-1.517780000	3.824652000	-0.405411000
H	-2.270582000	3.732440000	1.617996000
H	-0.606205000	3.648486000	-2.355685000
Si	-2.396855000	-1.226487000	-0.159018000
C	-2.789924000	-2.865121000	-1.001674000
H	-3.883026000	-3.012632000	-1.020019000
H	-2.431094000	-2.880598000	-2.043960000
H	-2.349266000	-3.724541000	-0.470729000
C	-2.841038000	-1.306347000	1.658861000
H	-2.233673000	-2.060136000	2.182886000
H	-2.678812000	-0.336747000	2.151539000
H	-3.903954000	-1.580044000	1.767774000
C	-3.291337000	0.160645000	-1.058910000
H	-3.131440000	1.136333000	-0.575263000
H	-2.962916000	0.232948000	-2.108221000
H	-4.374604000	-0.049677000	-1.057982000
H	-2.008227000	4.774367000	-0.630581000
C	2.059070000	-0.092269000	0.182080000
C	2.555150000	-0.490719000	-1.071212000
C	3.013571000	0.327848000	1.126749000
C	3.919612000	-0.471813000	-1.370808000
H	1.864703000	-0.830948000	-1.849173000

C	4.379248000	0.353844000	0.842169000
H	2.671621000	0.642540000	2.117249000
C	4.840310000	-0.047568000	-0.413029000
H	4.265244000	-0.792801000	-2.356879000
H	5.089112000	0.689600000	1.602421000
H	5.908480000	-0.030577000	-0.641345000
O	0.242340000	-0.332650000	2.003353000
H	0.848998000	-1.012768000	2.313739000

E(RwB97XD) = -1121.24004881

The computed reaction pathway for the coupling between 11 and TMS diazomethane 2

Start

C	1.852810000	0.696742000	-0.000017000
C	1.852812000	-0.696749000	0.000017000
C	3.024473000	-1.430891000	0.000034000
C	4.220259000	-0.698922000	0.000014000
C	4.220256000	0.698933000	-0.000021000
C	3.024464000	1.430894000	-0.000038000
H	3.013209000	-2.521148000	0.000058000
H	5.170561000	-1.235575000	0.000025000
H	5.170555000	1.235591000	-0.000036000
H	3.013191000	2.521151000	-0.000063000
B	-0.232668000	-0.000005000	0.000020000
O	0.557107000	-1.146881000	0.000012000
O	0.557101000	1.146870000	-0.000010000
C	-1.778767000	-0.000013000	0.000006000
C	-2.496448000	1.207744000	0.000034000
C	-2.496462000	-1.207749000	-0.000029000
C	-3.889662000	1.209677000	0.000032000
H	-1.956139000	2.157553000	0.000065000
C	-3.889685000	-1.209663000	-0.000039000
H	-1.956178000	-2.157572000	-0.000054000
C	-4.586726000	0.000009000	-0.000008000
H	-4.435236000	2.155424000	0.000059000
H	-4.435257000	-2.155410000	-0.000061000
H	-5.678979000	0.000025000	-0.000015000

E(RwB97XD) = -637.948272245

TS1

C	0.303538000	1.108248000	-0.936716000
H	0.651017000	0.782416000	-1.926390000
N	-0.859586000	1.742410000	-1.077217000

N	-1.859417000	2.254554000	-1.100426000
Si	1.554517000	2.106291000	0.131855000
C	1.463480000	3.901538000	-0.429627000
H	2.127965000	4.528908000	0.188452000
H	1.773684000	4.010649000	-1.481693000
H	0.441218000	4.304340000	-0.330365000
C	1.043360000	1.982820000	1.932023000
H	0.009998000	2.340284000	2.070502000
H	1.100850000	0.948647000	2.301707000
H	1.703288000	2.612541000	2.552322000
C	3.258531000	1.398956000	-0.204815000
H	3.387798000	0.399580000	0.236650000
H	3.455857000	1.333992000	-1.287013000
H	4.017865000	2.068045000	0.234542000
C	-2.249082000	-0.560795000	0.765576000
C	-2.403219000	-1.016138000	-0.548728000
C	-3.336724000	-0.233784000	1.554121000
C	-3.651286000	-1.164008000	-1.125220000
C	-4.609224000	-0.375651000	0.976134000
H	-3.204911000	0.122749000	2.576564000
C	-4.763116000	-0.830382000	-0.334061000
H	-3.760670000	-1.518169000	-2.151087000
H	-5.492833000	-0.124140000	1.565944000
H	-5.764878000	-0.930622000	-0.756090000
O	-1.181150000	-1.256734000	-1.101821000
O	-0.924972000	-0.503447000	1.082597000
B	-0.227629000	-0.825106000	-0.123117000
C	1.204205000	-1.473413000	-0.060004000
C	1.905200000	-1.817903000	-1.226409000
C	1.812321000	-1.735631000	1.176270000
C	3.176230000	-2.385936000	-1.162865000
H	1.450265000	-1.636428000	-2.204315000
C	3.084641000	-2.305107000	1.248493000
H	1.278859000	-1.493260000	2.098744000
C	3.771787000	-2.625946000	0.077437000
H	3.706664000	-2.642441000	-2.082621000
H	3.542590000	-2.498622000	2.221110000
H	4.768426000	-3.069517000	0.130303000

E(RwB97XD) = -1195.29668668

Intermediate

C	0.483666000	1.104101000	-0.879512000
H	0.868274000	0.934872000	-1.898870000
N	-0.556317000	1.989504000	-1.018031000
N	-1.442704000	2.658212000	-1.022841000

Si	1.881209000	1.931182000	0.221433000
C	2.017645000	3.706020000	-0.378718000
H	2.820333000	4.226555000	0.169943000
H	2.260706000	3.748303000	-1.453087000
H	1.084154000	4.269108000	-0.213144000
C	1.344557000	1.864811000	2.011565000
H	0.354442000	2.326891000	2.148723000
H	1.295302000	0.829143000	2.377921000
H	2.070490000	2.419243000	2.629680000
C	3.459032000	0.983651000	-0.120018000
H	3.422098000	-0.040738000	0.278811000
H	3.675961000	0.936987000	-1.198975000
H	4.295034000	1.512717000	0.368652000
C	-2.275540000	-0.274644000	0.690438000
C	-2.462069000	-0.679428000	-0.642640000
C	-3.347818000	-0.096771000	1.546055000
C	-3.727559000	-0.915325000	-1.150837000
C	-4.638281000	-0.335186000	1.034934000
H	-3.191069000	0.217034000	2.579602000
C	-4.823778000	-0.736513000	-0.285157000
H	-3.862712000	-1.229623000	-2.187265000
H	-5.503315000	-0.202980000	1.688062000
H	-5.833556000	-0.916643000	-0.659807000
O	-1.274936000	-0.784458000	-1.281025000
O	-0.960180000	-0.103070000	0.957609000
B	-0.227057000	-0.387048000	-0.299780000
C	0.980823000	-1.430852000	-0.152168000
C	1.703400000	-1.877386000	-1.270021000
C	1.364032000	-1.916905000	1.105713000
C	2.775141000	-2.759878000	-1.139254000
H	1.426226000	-1.525009000	-2.268679000
C	2.437519000	-2.798763000	1.249089000
H	0.806526000	-1.596568000	1.989669000
C	3.150738000	-3.218019000	0.125713000
H	3.323095000	-3.091483000	-2.024542000
H	2.718838000	-3.160578000	2.241119000
H	3.992540000	-3.905793000	0.233386000

E(RwB97XD) = -1195.30078242

TS2

C	0.717686000	0.882893000	-0.805757000
H	1.000112000	0.799036000	-1.868759000
N	-0.534099000	2.314276000	-1.122999000
N	-1.479693000	2.857038000	-0.977269000
Si	2.081648000	1.783125000	0.239138000

C	2.301477000	3.496753000	-0.504361000
H	3.165400000	3.992759000	-0.030770000
H	2.494255000	3.444644000	-1.588220000
H	1.419298000	4.135440000	-0.337959000
C	1.530999000	1.887772000	2.025392000
H	0.570504000	2.419687000	2.110755000
H	1.411654000	0.889287000	2.471819000
H	2.281884000	2.440995000	2.613672000
C	3.644834000	0.767760000	0.006089000
H	3.556293000	-0.227583000	0.467137000
H	3.881740000	0.639660000	-1.062057000
H	4.489769000	1.294345000	0.481522000
C	-2.307427000	-0.185545000	0.703364000
C	-2.520595000	-0.540764000	-0.641501000
C	-3.362877000	-0.064239000	1.589534000
C	-3.794337000	-0.778878000	-1.127419000
C	-4.661908000	-0.307104000	1.100740000
H	-3.186931000	0.209245000	2.631440000
C	-4.873099000	-0.656874000	-0.229384000
H	-3.949474000	-1.051664000	-2.172817000
H	-5.511552000	-0.220020000	1.781228000
H	-5.888150000	-0.840797000	-0.587694000
O	-1.352209000	-0.592932000	-1.316436000
O	-0.992925000	0.007182000	0.947584000
B	-0.283282000	-0.274009000	-0.328817000
C	0.887635000	-1.408778000	-0.192783000
C	1.498360000	-1.990023000	-1.315606000
C	1.307778000	-1.830730000	1.079750000
C	2.486552000	-2.965695000	-1.175479000
H	1.188957000	-1.684611000	-2.319229000
C	2.288396000	-2.809994000	1.227151000
H	0.839881000	-1.391815000	1.963943000
C	2.885096000	-3.375294000	0.097213000
H	2.946709000	-3.409005000	-2.061401000
H	2.590575000	-3.135776000	2.225187000
H	3.658596000	-4.138298000	0.209392000

E(RwB97XD) = -1195.27517006

<u>The computed reaction pathway for the coupling between 12 and TMS diazomethane 2</u>

Start

B	-1.385006000	-0.090443000	0.000598000
B	0.613872000	1.243953000	0.000016000
B	0.769739000	-1.154293000	0.000313000
O	-0.609586000	-1.233999000	0.001098000

O	-0.764139000	1.143901000	0.000346000
O	1.372290000	0.089058000	-0.000455000
C	-2.939081000	-0.191678000	0.000294000
C	-3.736196000	0.965118000	-0.000470000
C	-3.578635000	-1.442420000	0.000583000
C	-5.126922000	0.877245000	-0.000948000
H	-3.257434000	1.947259000	-0.000675000
C	-4.968952000	-1.536678000	0.000129000
H	-2.976054000	-2.353862000	0.001171000
C	-5.743795000	-0.375264000	-0.000639000
H	-5.733623000	1.785174000	-0.001545000
H	-5.451869000	-2.516012000	0.000369000
C	1.634984000	-2.449161000	0.000168000
C	1.032728000	-3.718255000	0.000417000
C	3.037862000	-2.376528000	-0.000378000
C	1.805070000	-4.878166000	0.000110000
H	-0.057174000	-3.795543000	0.000830000
C	3.815563000	-3.532891000	-0.000676000
H	3.524817000	-1.398448000	-0.000559000
C	3.198175000	-4.785084000	-0.000459000
H	1.322813000	-5.857835000	0.000295000
H	4.905126000	-3.460460000	-0.001090000
C	1.303266000	2.640492000	0.000055000
C	2.703663000	2.752218000	-0.000081000
C	0.540198000	3.820013000	0.000170000
C	3.323194000	4.000405000	-0.000086000
H	3.314912000	1.846501000	-0.000211000
C	1.154046000	5.071049000	0.000165000
H	-0.550474000	3.754513000	0.000210000
C	2.547251000	5.161136000	0.000030000
H	4.412842000	4.071592000	-0.000208000
H	0.547436000	5.979035000	0.000257000
H	-6.833733000	-0.446595000	-0.001023000
H	3.030776000	6.140505000	0.000022000
H	3.805593000	-5.692893000	-0.000704000

E(RwB97XD) = -995.282713340

TS1

C	-1.287705000	-0.746504000	1.514802000
H	-1.129996000	-1.814057000	1.714391000
B	-0.467798000	-0.634651000	-0.506344000
O	0.713962000	-1.388355000	-0.285926000
O	-0.304972000	0.760824000	-0.654255000
N	-0.344361000	-0.026981000	2.104218000
N	0.455828000	0.675748000	2.469388000

B	0.924776000	1.353016000	-0.573621000
B	1.944127000	-0.793553000	-0.249272000
O	2.062826000	0.575554000	-0.428984000
C	1.050764000	2.909745000	-0.645971000
C	-0.096227000	3.714416000	-0.744886000
C	2.303526000	3.542025000	-0.600059000
C	0.001781000	5.103890000	-0.795129000
H	-1.079303000	3.238875000	-0.779899000
C	2.409616000	4.931212000	-0.653448000
H	3.208492000	2.934650000	-0.520657000
C	1.257124000	5.713544000	-0.749853000
H	-0.900788000	5.714306000	-0.869854000
H	3.391496000	5.407855000	-0.617301000
C	3.227569000	-1.649810000	0.006739000
C	3.140499000	-3.039564000	0.190557000
C	4.495559000	-1.050046000	0.071428000
C	4.279463000	-3.805468000	0.434322000
H	2.162754000	-3.525079000	0.141154000
C	5.639193000	-1.809475000	0.314580000
H	4.584732000	0.029806000	-0.069469000
C	5.530952000	-3.189386000	0.496748000
H	4.193737000	-4.884948000	0.576376000
H	6.617495000	-1.326705000	0.363608000
C	-1.649451000	-1.333303000	-1.289750000
C	-1.869287000	-2.716232000	-1.191646000
C	-2.505384000	-0.589010000	-2.114459000
C	-2.919338000	-3.331599000	-1.870775000
H	-1.209841000	-3.322158000	-0.563928000
C	-3.556358000	-1.197261000	-2.802962000
H	-2.342730000	0.486259000	-2.221649000
C	-3.769414000	-2.570265000	-2.676490000
H	-3.078420000	-4.407905000	-1.773399000
H	-4.211216000	-0.598157000	-3.439912000
Si	-3.052277000	-0.027355000	1.768333000
C	-3.292773000	0.205813000	3.620759000
H	-2.536134000	0.892557000	4.036031000
H	-4.286567000	0.636865000	3.828758000
H	-3.216983000	-0.753643000	4.157890000
C	-3.137174000	1.633811000	0.901807000
H	-2.362811000	2.317010000	1.286954000
H	-2.998466000	1.526747000	-0.183601000
H	-4.118924000	2.102432000	1.083230000
C	-4.267641000	-1.286003000	1.097435000
H	-4.219963000	-1.364234000	0.001208000
H	-4.084138000	-2.283435000	1.528653000
H	-5.291410000	-0.983759000	1.375936000
H	1.338292000	6.802145000	-0.790432000

H	-4.594931000	-3.049060000	-3.207950000
H	6.425422000	-3.786201000	0.688970000

E(RwB97XD) = -1552.63721732

Intermediate

C	-1.346687000	-0.945466000	1.423248000
H	-1.297944000	-2.020593000	1.660564000
B	-0.518632000	-0.673099000	-0.111575000
O	0.791423000	-1.317178000	0.045340000
O	-0.416955000	0.774005000	-0.273724000
N	-0.545779000	-0.319701000	2.340059000
N	0.140291000	0.287217000	2.969365000
B	0.765442000	1.428865000	-0.328101000
B	1.955506000	-0.632419000	-0.028151000
O	1.969697000	0.745538000	-0.217329000
C	0.793888000	2.989427000	-0.508671000
C	-0.399925000	3.713240000	-0.660933000
C	2.003252000	3.702171000	-0.519209000
C	-0.389963000	5.098534000	-0.817295000
H	-1.351075000	3.175302000	-0.655126000
C	2.022634000	5.088260000	-0.673690000
H	2.944450000	3.158990000	-0.403419000
C	0.824200000	5.788258000	-0.823297000
H	-1.328936000	5.643965000	-0.935796000
H	2.973460000	5.625889000	-0.678765000
C	3.328633000	-1.384705000	0.108069000
C	3.370133000	-2.781516000	0.246903000
C	4.546879000	-0.686992000	0.092321000
C	4.582374000	-3.459808000	0.365016000
H	2.432142000	-3.342402000	0.258077000
C	5.764191000	-1.357030000	0.212136000
H	4.537830000	0.400384000	-0.016547000
C	5.782605000	-2.746224000	0.348481000
H	4.594978000	-4.547109000	0.469314000
H	6.702047000	-0.797200000	0.198149000
C	-1.431495000	-1.380260000	-1.232088000
C	-1.563260000	-2.777018000	-1.286610000
C	-2.142018000	-0.626059000	-2.176393000
C	-2.382809000	-3.396637000	-2.229719000
H	-1.013034000	-3.397611000	-0.572595000
C	-2.964381000	-1.234859000	-3.127235000
H	-2.046530000	0.462790000	-2.164900000
C	-3.092169000	-2.623965000	-3.152156000
H	-2.469991000	-4.485775000	-2.248519000
H	-3.507428000	-0.623654000	-3.852362000

Si	-3.188135000	-0.303635000	1.610236000
C	-3.526915000	-0.249105000	3.457372000
H	-2.858229000	0.459994000	3.973058000
H	-4.563065000	0.084035000	3.635839000
H	-3.405761000	-1.241153000	3.922379000
C	-3.317621000	1.407576000	0.867086000
H	-2.594875000	2.095764000	1.332584000
H	-3.133094000	1.386700000	-0.216335000
H	-4.331224000	1.805102000	1.043996000
C	-4.273215000	-1.572222000	0.762210000
H	-4.157221000	-1.549073000	-0.331540000
H	-4.055625000	-2.591293000	1.119365000
H	-5.327105000	-1.348978000	1.000766000
H	0.836237000	6.873759000	-0.945523000
H	-3.737055000	-3.104569000	-3.891450000
H	6.734463000	-3.274253000	0.441073000

E(RwB97XD) = -1552.64139290

TS2

C	-1.477169000	-1.008652000	1.147705000
H	-1.399849000	-2.061076000	1.468201000
B	-0.514387000	-0.651608000	-0.095249000
O	0.781423000	-1.337174000	0.001932000
O	-0.384488000	0.792090000	-0.299823000
N	-0.425251000	-0.356726000	2.600244000
N	0.352209000	0.288869000	3.036117000
B	0.811722000	1.415690000	-0.344568000
B	1.956287000	-0.675385000	-0.063164000
O	2.001250000	0.705724000	-0.226589000
C	0.880304000	2.977707000	-0.514909000
C	-0.295777000	3.736730000	-0.629958000
C	2.108278000	3.657304000	-0.545059000
C	-0.250731000	5.123155000	-0.769792000
H	-1.261876000	3.226230000	-0.606246000
C	2.163070000	5.044180000	-0.684432000
H	3.036369000	3.086930000	-0.456964000
C	0.981666000	5.779254000	-0.796408000
H	-1.176743000	5.695870000	-0.858145000
H	3.128320000	5.555200000	-0.705585000
C	3.314876000	-1.458538000	0.060520000
C	3.327429000	-2.857345000	0.184925000
C	4.547724000	-0.786986000	0.052850000
C	4.525033000	-3.562349000	0.296195000
H	2.377824000	-3.398503000	0.191247000
C	5.750755000	-1.483660000	0.165810000

H	4.561733000	0.301508000	-0.043536000
C	5.740183000	-2.874241000	0.287565000
H	4.514504000	-4.650705000	0.389717000
H	6.700121000	-0.943394000	0.158520000
C	-1.518976000	-1.347547000	-1.190549000
C	-1.652843000	-2.742066000	-1.289893000
C	-2.276430000	-0.550039000	-2.064597000
C	-2.502763000	-3.322268000	-2.232701000
H	-1.071459000	-3.387932000	-0.626014000
C	-3.118077000	-1.122100000	-3.016980000
H	-2.184677000	0.536711000	-2.002897000
C	-3.238758000	-2.511754000	-3.097497000
H	-2.590167000	-4.409393000	-2.294816000
H	-3.684813000	-0.484718000	-3.699752000
Si	-3.214684000	-0.253868000	1.566525000
C	-3.409575000	-0.331333000	3.436594000
H	-2.730668000	0.365915000	3.953508000
H	-4.441816000	-0.053088000	3.708051000
H	-3.222911000	-1.347741000	3.820217000
C	-3.303434000	1.515343000	0.959100000
H	-2.485674000	2.115296000	1.387635000
H	-3.235544000	1.565521000	-0.137501000
H	-4.260531000	1.966957000	1.269462000
C	-4.473239000	-1.380082000	0.745135000
H	-4.404290000	-1.343850000	-0.352614000
H	-4.343961000	-2.424852000	1.070396000
H	-5.487639000	-1.058999000	1.037541000
H	1.021315000	6.865551000	-0.905312000
H	-3.904214000	-2.962080000	-3.837415000
H	6.680651000	-3.423090000	0.375428000

E(RwB97XD) = -1552.61582776

Product

C	-1.554559000	-1.437364000	-0.441194000
H	-1.345985000	-2.221247000	-1.191215000
B	-0.295329000	-0.520254000	-0.381866000
O	0.962386000	-1.089854000	-0.516206000
O	-0.373620000	0.842831000	-0.161808000
B	0.761020000	1.622493000	-0.064815000
B	2.108525000	-0.337554000	-0.370553000
O	2.003125000	1.023756000	-0.153538000
C	0.634897000	3.160319000	0.147107000
C	-0.625984000	3.779877000	0.157539000
C	1.773868000	3.961375000	0.331081000
C	-0.746715000	5.155342000	0.346021000

H	-1.523046000	3.173360000	0.010091000
C	1.659076000	5.336747000	0.524101000
H	2.763422000	3.497935000	0.324543000
C	0.397197000	5.934201000	0.531232000
H	-1.733176000	5.623622000	0.349042000
H	2.553504000	5.946249000	0.669007000
C	3.505906000	-1.023028000	-0.437789000
C	3.610553000	-2.415913000	-0.587955000
C	4.688548000	-0.271636000	-0.339997000
C	4.855390000	-3.040415000	-0.636146000
H	2.700563000	-3.015920000	-0.664316000
C	5.936471000	-0.890120000	-0.390682000
H	4.627881000	0.813072000	-0.222248000
C	6.019655000	-2.276098000	-0.538301000
H	4.920951000	-4.124429000	-0.750763000
H	6.847808000	-0.293350000	-0.314248000
C	-2.880587000	-0.786679000	-0.746931000
C	-3.709048000	-1.312931000	-1.749461000
C	-3.349970000	0.325159000	-0.028510000
C	-4.954358000	-0.750678000	-2.030100000
H	-3.369149000	-2.180708000	-2.320994000
C	-4.594561000	0.888852000	-0.306895000
H	-2.729639000	0.758354000	0.757122000
C	-5.405185000	0.355050000	-1.309305000
H	-5.575947000	-1.181653000	-2.818278000
H	-4.932625000	1.754105000	0.267996000
Si	-1.664226000	-2.480847000	1.179605000
C	-3.145143000	-3.632443000	1.022469000
H	-3.049052000	-4.288053000	0.141159000
H	-3.224014000	-4.275680000	1.915194000
H	-4.086888000	-3.068154000	0.928129000
C	-0.086762000	-3.493478000	1.364274000
H	0.109626000	-4.093806000	0.460846000
H	0.789669000	-2.852097000	1.545622000
H	-0.182154000	-4.188582000	2.215464000
C	-1.871129000	-1.357435000	2.677500000
H	-1.066457000	-0.605250000	2.730952000
H	-2.836370000	-0.827170000	2.660219000
H	-1.833461000	-1.954957000	3.604110000
H	0.304969000	7.012259000	0.681135000
H	-6.379560000	0.797374000	-1.526300000
H	6.996732000	-2.762925000	-0.576498000

E(RwB97XD) = -1443.23517438

The computed reaction pathway for the coupling between 12 and TMS diazomethane 2; addition of the second equivalent of 2 to the benzyl center (*anti*-isomer)

Start

C	-1.554559000	-1.437364000	-0.441194000
H	-1.345985000	-2.221247000	-1.191215000
B	-0.295329000	-0.520254000	-0.381866000
O	0.962386000	-1.089854000	-0.516206000
O	-0.373620000	0.842831000	-0.161808000
B	0.761020000	1.622493000	-0.064815000
B	2.108525000	-0.337554000	-0.370553000
O	2.003125000	1.023756000	-0.153538000
C	0.634897000	3.160319000	0.147107000
C	-0.625984000	3.779877000	0.157539000
C	1.773868000	3.961375000	0.331081000
C	-0.746715000	5.155342000	0.346021000
H	-1.523046000	3.173360000	0.010091000
C	1.659076000	5.336747000	0.524101000
H	2.763422000	3.497935000	0.324543000
C	0.397197000	5.934201000	0.531232000
H	-1.733176000	5.623622000	0.349042000
H	2.553504000	5.946249000	0.669007000
C	3.505906000	-1.023028000	-0.437789000
C	3.610553000	-2.415913000	-0.587955000
C	4.688548000	-0.271636000	-0.339997000
C	4.855390000	-3.040415000	-0.636146000
H	2.700563000	-3.015920000	-0.664316000
C	5.936471000	-0.890120000	-0.390682000
H	4.627881000	0.813072000	-0.222248000
C	6.019655000	-2.276098000	-0.538301000
H	4.920951000	-4.124429000	-0.750763000
H	6.847808000	-0.293350000	-0.314248000
C	-2.880587000	-0.786679000	-0.746931000
C	-3.709048000	-1.312931000	-1.749461000
C	-3.349970000	0.325159000	-0.028510000
C	-4.954358000	-0.750678000	-2.030100000
H	-3.369149000	-2.180708000	-2.320994000
C	-4.594561000	0.888852000	-0.306895000
H	-2.729639000	0.758354000	0.757122000
C	-5.405185000	0.355050000	-1.309305000
H	-5.575947000	-1.181653000	-2.818278000
H	-4.932625000	1.754105000	0.267996000
Si	-1.664226000	-2.480847000	1.179605000
C	-3.145143000	-3.632443000	1.022469000
H	-3.049052000	-4.288053000	0.141159000
H	-3.224014000	-4.275680000	1.915194000
H	-4.086888000	-3.068154000	0.928129000
C	-0.086762000	-3.493478000	1.364274000

H	0.109626000	-4.093806000	0.460846000
H	0.789669000	-2.852097000	1.545622000
H	-0.182154000	-4.188582000	2.215464000
C	-1.871129000	-1.357435000	2.677500000
H	-1.066457000	-0.605250000	2.730952000
H	-2.836370000	-0.827170000	2.660219000
H	-1.833461000	-1.954957000	3.604110000
H	0.304969000	7.012259000	0.681135000
H	-6.379560000	0.797374000	-1.526300000
H	6.996732000	-2.762925000	-0.576498000

E(RwB97XD) = -1443.23517438

TS1

C	1.329317000	-1.390714000	-0.454019000
H	2.169715000	-1.473021000	0.252598000
B	0.208633000	-0.426189000	0.158711000
O	-1.139996000	-0.860277000	0.170452000
O	0.395524000	0.968334000	0.045227000
B	-0.641486000	1.825951000	-0.183246000
B	-2.182299000	0.001513000	-0.035653000
O	-1.939594000	1.344801000	-0.266336000
C	-0.362258000	3.352537000	-0.369408000
C	0.957209000	3.831531000	-0.420051000
C	-1.410811000	4.277652000	-0.494823000
C	1.222739000	5.189619000	-0.586629000
H	1.784501000	3.122859000	-0.333212000
C	-1.152678000	5.638001000	-0.658623000
H	-2.444368000	3.924115000	-0.461069000
C	0.165947000	6.094662000	-0.704224000
H	2.254527000	5.545445000	-0.626716000
H	-1.979097000	6.345983000	-0.751785000
C	-3.650020000	-0.536406000	-0.028951000
C	-3.909314000	-1.905977000	0.144656000
C	-4.741928000	0.330433000	-0.195670000
C	-5.214326000	-2.395737000	0.150749000
H	-3.071478000	-2.595960000	0.273803000
C	-6.049931000	-0.151753000	-0.188007000
H	-4.561273000	1.399464000	-0.331924000
C	-6.286487000	-1.516736000	-0.014710000
H	-5.398890000	-3.463736000	0.284722000
H	-6.888286000	0.536054000	-0.317506000
C	1.890355000	-0.827893000	-1.740140000
C	3.274766000	-0.765849000	-1.957305000
C	1.049236000	-0.358087000	-2.762623000
C	3.800846000	-0.254781000	-3.144489000

H	3.952029000	-1.126919000	-1.178375000
C	1.570052000	0.154704000	-3.949020000
H	-0.035140000	-0.391891000	-2.627570000
C	2.950736000	0.209533000	-4.147769000
H	4.883819000	-0.217896000	-3.283540000
H	0.890596000	0.516676000	-4.723944000
Si	0.765014000	-3.199248000	-0.703899000
C	2.248649000	-4.197849000	-1.310643000
H	3.087580000	-4.146779000	-0.596016000
H	1.977058000	-5.260427000	-1.431481000
H	2.611263000	-3.832498000	-2.285458000
C	0.184262000	-3.932239000	0.936967000
H	0.970152000	-3.873021000	1.708884000
H	-0.713443000	-3.413673000	1.310048000
H	-0.071121000	-4.997589000	0.807873000
C	-0.614403000	-3.337829000	-1.981906000
H	-1.480807000	-2.715548000	-1.709560000
H	-0.268113000	-3.029197000	-2.981646000
H	-0.954949000	-4.384750000	-2.056506000
H	0.370934000	7.159832000	-0.833506000
H	3.359612000	0.613014000	-5.076293000
H	-7.310414000	-1.897124000	-0.009210000
C	0.618973000	-0.664163000	2.355755000
N	-0.336442000	0.158165000	2.745437000
N	-1.127684000	0.932117000	2.956475000
Si	2.392322000	-0.172340000	2.897151000
C	3.089916000	1.130087000	1.742912000
H	2.483316000	2.048447000	1.780268000
H	4.115784000	1.386299000	2.057618000
H	3.122114000	0.782060000	0.699656000
C	2.283393000	0.516856000	4.643304000
H	1.885398000	-0.231946000	5.347381000
H	3.281267000	0.824856000	4.998277000
H	1.628964000	1.404005000	4.680019000
C	3.390596000	-1.763078000	2.867927000
H	4.413217000	-1.558528000	3.227270000
H	2.947071000	-2.523047000	3.531421000
H	3.473606000	-2.189030000	1.855697000
H	0.300299000	-1.704674000	2.487522000

E(RwB97XD) = -2000.58628861

Intermediate

C	-0.827769000	-1.371998000	0.960067000
H	-1.752295000	-1.914387000	0.699139000
B	-0.183296000	-0.727127000	-0.400691000

O	1.293022000	-0.709391000	-0.405874000
O	-0.727671000	0.598056000	-0.711054000
B	0.005155000	1.727235000	-0.599812000
B	2.013697000	0.432698000	-0.455709000
O	1.392868000	1.675238000	-0.505474000
C	-0.704548000	3.127968000	-0.571412000
C	-2.101612000	3.206038000	-0.453246000
C	0.022150000	4.327404000	-0.625885000
C	-2.753355000	4.437050000	-0.398244000
H	-2.679319000	2.280464000	-0.392596000
C	-0.622135000	5.563666000	-0.576466000
H	1.111454000	4.289654000	-0.708501000
C	-2.012469000	5.619023000	-0.462176000
H	-3.840642000	4.478392000	-0.301227000
H	-0.041030000	6.487520000	-0.623112000
C	3.584616000	0.377446000	-0.440435000
C	4.259582000	-0.853894000	-0.410294000
C	4.354948000	1.550999000	-0.452593000
C	5.652412000	-0.914527000	-0.393742000
H	3.677050000	-1.778719000	-0.397737000
C	5.748586000	1.500167000	-0.434671000
H	3.850586000	2.520234000	-0.474731000
C	6.399205000	0.265382000	-0.405917000
H	6.159399000	-1.881828000	-0.370157000
H	6.330960000	2.424270000	-0.443156000
C	-1.231409000	-0.303739000	1.945384000
C	-2.553249000	-0.207636000	2.406971000
C	-0.317291000	0.659556000	2.407047000
C	-2.951740000	0.809264000	3.275963000
H	-3.287716000	-0.944294000	2.069110000
C	-0.710419000	1.679851000	3.270649000
H	0.723031000	0.614703000	2.078798000
C	-2.032685000	1.763943000	3.709931000
H	-3.990816000	0.856968000	3.610566000
H	0.023735000	2.419032000	3.599125000
Si	0.233837000	-2.719212000	1.787925000
C	-0.839067000	-3.598930000	3.073020000
H	-1.710390000	-4.087383000	2.603989000
H	-0.265380000	-4.377695000	3.603832000
H	-1.219085000	-2.889632000	3.827449000
C	0.785188000	-4.000802000	0.512158000
H	-0.073172000	-4.431566000	-0.030941000
H	1.479331000	-3.559680000	-0.221427000
H	1.310129000	-4.832883000	1.011125000
C	1.758874000	-2.037084000	2.664528000
H	2.387921000	-1.446421000	1.980502000
H	1.486414000	-1.400581000	3.521657000

H	2.368058000	-2.871926000	3.051853000
H	-2.519519000	6.585809000	-0.419472000
H	-2.342435000	2.565961000	4.382961000
H	7.490728000	0.222264000	-0.392075000
C	-0.624288000	-1.737048000	-1.757151000
N	-0.036623000	-1.094125000	-2.816243000
N	0.424092000	-0.447496000	-3.591955000
Si	-2.529328000	-2.029774000	-2.119189000
C	-3.527781000	-0.572783000	-1.506008000
H	-3.238834000	0.348251000	-2.034184000
H	-4.596712000	-0.763306000	-1.700862000
H	-3.391011000	-0.409136000	-0.427203000
C	-2.699721000	-2.228127000	-3.979178000
H	-2.083898000	-3.061138000	-4.355811000
H	-3.751159000	-2.444733000	-4.232226000
H	-2.411745000	-1.310995000	-4.519492000
C	-2.948340000	-3.636080000	-1.246523000
H	-3.998611000	-3.895668000	-1.462256000
H	-2.317731000	-4.462000000	-1.612943000
H	-2.839728000	-3.568856000	-0.153458000
H	-0.112591000	-2.709436000	-1.681833000

E(RwB97XD) = -2000.59307823

TS2

C	-0.748983000	-1.579618000	0.756644000
H	-1.396761000	-2.401431000	0.390099000
B	-0.064489000	-0.691009000	-0.512624000
O	1.406465000	-0.649306000	-0.467073000
O	-0.638409000	0.648019000	-0.636411000
B	0.073164000	1.774282000	-0.435891000
B	2.100914000	0.503210000	-0.354382000
O	1.459914000	1.736434000	-0.313675000
C	-0.658918000	3.159503000	-0.314843000
C	-2.057390000	3.210147000	-0.192353000
C	0.049956000	4.370573000	-0.291234000
C	-2.725902000	4.425310000	-0.052352000
H	-2.622192000	2.274609000	-0.196417000
C	-0.611446000	5.591621000	-0.159142000
H	1.139413000	4.354308000	-0.378360000
C	-2.002018000	5.619554000	-0.038082000
H	-3.813343000	4.444200000	0.049846000
H	-0.044029000	6.525018000	-0.146155000
C	3.669523000	0.470826000	-0.250466000
C	4.355503000	-0.751665000	-0.164670000
C	4.425407000	1.653570000	-0.229564000

C	5.745259000	-0.794846000	-0.062224000
H	3.783179000	-1.682986000	-0.169162000
C	5.816407000	1.620035000	-0.130852000
H	3.911941000	2.616269000	-0.291846000
C	6.478108000	0.393839000	-0.047031000
H	6.260437000	-1.755399000	0.008577000
H	6.387087000	2.551398000	-0.117235000
C	-1.600891000	-0.725489000	1.654372000
C	-2.949281000	-1.028539000	1.895154000
C	-1.068431000	0.408926000	2.291891000
C	-3.737989000	-0.229554000	2.724840000
H	-3.390629000	-1.908603000	1.420184000
C	-1.850422000	1.209376000	3.120121000
H	-0.019348000	0.669899000	2.136225000
C	-3.193762000	0.897724000	3.339143000
H	-4.786061000	-0.490565000	2.889068000
H	-1.407388000	2.088931000	3.592396000
Si	0.540473000	-2.598978000	1.731251000
C	-0.426419000	-3.644427000	2.977257000
H	-1.160003000	-4.294813000	2.470964000
H	0.248588000	-4.291236000	3.563515000
H	-0.977815000	-3.006609000	3.687997000
C	1.432123000	-3.771162000	0.549753000
H	0.722093000	-4.468623000	0.073106000
H	1.962695000	-3.218073000	-0.240302000
H	2.170229000	-4.379733000	1.099015000
C	1.779488000	-1.561755000	2.702903000
H	2.337107000	-0.860132000	2.065508000
H	1.281595000	-0.989578000	3.502220000
H	2.511858000	-2.232127000	3.184924000
H	-2.522069000	6.573996000	0.071763000
H	-3.809478000	1.527914000	3.984168000
H	7.567150000	0.364025000	0.034093000
C	-0.616884000	-1.675287000	-1.649383000
N	0.314438000	-1.003873000	-3.155402000
N	0.932982000	-0.251212000	-3.667541000
Si	-2.447014000	-1.792263000	-2.296077000
C	-3.399102000	-0.244470000	-1.849167000
H	-2.960618000	0.642160000	-2.331263000
H	-4.442723000	-0.345409000	-2.191447000
H	-3.402248000	-0.080859000	-0.761145000
C	-2.365616000	-2.023268000	-4.161285000
H	-1.739337000	-2.885638000	-4.442168000
H	-3.382896000	-2.204766000	-4.546833000
H	-1.974181000	-1.126269000	-4.667505000
C	-3.186529000	-3.328675000	-1.501432000
H	-4.162269000	-3.540858000	-1.970150000

H	-2.541248000	-4.209455000	-1.650042000
H	-3.356433000	-3.198660000	-0.421838000
H	-0.095129000	-2.645040000	-1.689871000

E(RwB97XD) = -2000.56667410

The computed reaction pathway for the coupling between 12 and diazomethane

TS1

C	-1.088496000	-1.240701000	2.335988000
H	-1.003076000	-2.314441000	2.518654000
B	-0.920519000	-0.962981000	0.147924000
O	0.453633000	-1.234127000	-0.018256000
O	-1.322172000	0.387827000	0.055145000
N	-0.009551000	-0.577049000	2.729374000
N	0.946697000	-0.007321000	2.889176000
B	-0.413691000	1.388102000	-0.161798000
B	1.361890000	-0.227388000	-0.212288000
O	0.929729000	1.085301000	-0.310582000
C	-0.882465000	2.877270000	-0.240106000
C	-2.241218000	3.211769000	-0.118454000
C	0.042982000	3.916624000	-0.428384000
C	-2.663738000	4.538446000	-0.183291000
H	-2.976467000	2.416573000	0.027699000
C	-0.371957000	5.245821000	-0.492548000
H	1.104736000	3.677339000	-0.525106000
C	-1.727296000	5.557327000	-0.369865000
H	-3.724262000	4.781795000	-0.088795000
H	0.360899000	6.042300000	-0.638398000
C	2.885715000	-0.554154000	-0.328504000
C	3.342294000	-1.879746000	-0.248185000
C	3.834575000	0.465258000	-0.508286000
C	4.699775000	-2.180260000	-0.345137000
H	2.618726000	-2.686486000	-0.107822000
C	5.194205000	0.172394000	-0.605051000
H	3.500187000	1.503627000	-0.572282000
C	5.627626000	-1.152188000	-0.523505000
H	5.037675000	-3.216890000	-0.281767000
H	5.919725000	0.976827000	-0.743972000
C	-1.963544000	-2.083308000	-0.239988000
C	-1.551414000	-3.402391000	-0.485792000
C	-3.330876000	-1.788250000	-0.353883000
C	-2.470223000	-4.393509000	-0.829890000
H	-0.490632000	-3.655153000	-0.408914000
C	-4.256403000	-2.773855000	-0.696882000
H	-3.675116000	-0.766423000	-0.173867000

C	-3.826895000	-4.080514000	-0.934589000
H	-2.129472000	-5.413975000	-1.019762000
H	-5.316309000	-2.523122000	-0.781586000
H	-2.055061000	6.598117000	-0.420563000
H	-4.548739000	-4.854807000	-1.203978000
H	6.692368000	-1.384361000	-0.599128000
H	-2.018991000	-0.727207000	2.588197000

E(RwB97XD) = -1143.98040924

Intermediate

C	-0.052765000	1.828679000	2.328522000
H	-0.976616000	2.351702000	2.609984000
B	-0.041701000	1.352442000	0.659350000
O	-1.238060000	0.529195000	0.467282000
O	1.201438000	0.604738000	0.465009000
N	-0.023840000	0.641451000	3.058215000
N	0.003277000	-0.385807000	3.466623000
B	1.221074000	-0.670616000	0.012559000
B	-1.178747000	-0.743325000	0.010273000
O	0.041436000	-1.359582000	-0.242274000
C	2.590191000	-1.404982000	-0.222928000
C	3.806874000	-0.747772000	0.022270000
C	2.637839000	-2.730970000	-0.681560000
C	5.028113000	-1.387998000	-0.183461000
H	3.790810000	0.285172000	0.379081000
C	3.854930000	-3.379394000	-0.890193000
H	1.703390000	-3.261998000	-0.879073000
C	5.052698000	-2.707316000	-0.640247000
H	5.964107000	-0.859580000	0.011396000
H	3.872076000	-4.411312000	-1.247883000
C	-2.498964000	-1.559251000	-0.235189000
C	-3.755529000	-0.970687000	-0.019034000
C	-2.461879000	-2.890993000	-0.678015000
C	-4.934075000	-1.682275000	-0.238774000
H	-3.805020000	0.066029000	0.323505000
C	-3.635794000	-3.610889000	-0.899479000
H	-1.494903000	-3.369132000	-0.853203000
C	-4.874511000	-3.005976000	-0.679386000
H	-5.902279000	-1.206116000	-0.068036000
H	-3.587116000	-4.646153000	-1.244429000
C	-0.084848000	2.743402000	-0.154233000
C	-1.299002000	3.400547000	-0.408984000
C	1.091029000	3.349906000	-0.621127000
C	-1.341016000	4.612954000	-1.098949000
H	-2.234257000	2.947670000	-0.067153000

C	1.060822000	4.561568000	-1.314173000
H	2.050186000	2.855073000	-0.445763000
C	-0.157816000	5.197986000	-1.554296000
H	-2.299500000	5.102871000	-1.287581000
H	1.990696000	5.009763000	-1.672996000
H	6.007479000	-3.212708000	-0.802795000
H	-0.186314000	6.145821000	-2.096681000
H	-5.795739000	-3.566753000	-0.853354000
H	0.847524000	2.393600000	2.605493000

E(RwB97XD) = -1143.98683078

TS2

C	0.001253000	2.094704000	2.027173000
H	-0.910951000	2.600922000	2.367789000
B	0.000855000	1.317794000	0.628942000
O	-1.223457000	0.553179000	0.415872000
O	1.224093000	0.551364000	0.416204000
N	-0.000025000	0.776785000	3.323679000
N	-0.001159000	-0.287141000	3.606478000
B	1.200322000	-0.735556000	0.001298000
B	-1.201457000	-0.733813000	0.001080000
O	-0.001025000	-1.397809000	-0.222755000
C	2.544699000	-1.516842000	-0.228815000
C	3.783369000	-0.889751000	-0.017589000
C	2.547395000	-2.856219000	-0.649521000
C	4.982442000	-1.571391000	-0.220609000
H	3.802457000	0.152891000	0.309686000
C	3.742027000	-3.546147000	-0.855121000
H	1.595075000	-3.364606000	-0.819599000
C	4.962269000	-2.903202000	-0.639914000
H	5.936063000	-1.065682000	-0.052679000
H	3.723910000	-4.587881000	-1.183179000
C	-2.546895000	-1.513241000	-0.229144000
C	-3.784735000	-0.884514000	-0.017917000
C	-2.551363000	-2.852605000	-0.649875000
C	-4.984707000	-1.564577000	-0.220929000
H	-3.802441000	0.158149000	0.309372000
C	-3.746900000	-3.540953000	-0.855491000
H	-1.599716000	-3.362241000	-0.819960000
C	-4.966292000	-2.896411000	-0.640245000
H	-5.937661000	-1.057625000	-0.052968000
H	-3.730156000	-4.582704000	-1.183571000
C	0.002078000	2.765382000	-0.160519000
C	-1.202426000	3.396017000	-0.513170000
C	1.207693000	3.393976000	-0.513023000

C	-1.204935000	4.593367000	-1.227788000
H	-2.151251000	2.928095000	-0.238163000
C	1.212320000	4.591312000	-1.227650000
H	2.155685000	2.924435000	-0.237919000
C	0.004224000	5.193140000	-1.585361000
H	-2.151147000	5.062783000	-1.506369000
H	2.159362000	5.059109000	-1.506134000
H	5.899634000	-3.440796000	-0.800493000
H	0.005056000	6.132729000	-2.142326000
H	-5.904360000	-3.432787000	-0.800796000
H	0.914152000	2.599442000	2.368095000

E(RwB97XD) = -1143.96745609

The computed reaction pathway for the coupling between 13 and TMS diazomethane 2

Start

B	-1.730105000	-0.000015000	-0.000120000
C	-0.184181000	-0.000020000	0.000002000
C	0.530951000	-1.209714000	-0.000081000
C	0.530448000	1.209403000	0.000158000
C	1.923471000	-1.210742000	0.000003000
H	-0.009861000	-2.158773000	-0.000215000
C	1.923433000	1.210773000	0.000233000
H	-0.010204000	2.158587000	0.000207000
C	2.618828000	0.000251000	0.000169000
H	2.470746000	-2.155365000	-0.000068000
H	2.469823000	2.155887000	0.000343000
H	3.711183000	0.000340000	0.000225000
F	-2.446598000	-1.131500000	-0.000291000
F	-2.446610000	1.131466000	-0.000020000

E(RwB97XD) = -456.212021439

TS1

C	1.377746000	0.403706000	-0.814794000
H	1.046974000	0.540042000	-1.850488000
B	-0.403641000	1.779674000	0.175482000
N	2.308698000	1.272057000	-0.486396000
N	3.059066000	2.022996000	-0.100525000
C	-1.577879000	0.759312000	0.080898000
C	-2.038969000	0.087579000	1.223069000
C	-2.204801000	0.499675000	-1.148031000
C	-3.088189000	-0.827024000	1.139984000
H	-1.573221000	0.284620000	2.191335000

C	-3.249993000	-0.416851000	-1.237530000
H	-1.864437000	1.016779000	-2.048565000
C	-3.691120000	-1.083488000	-0.092277000
H	-3.437777000	-1.341568000	2.037416000
H	-3.725560000	-0.612610000	-2.200803000
Si	1.545701000	-1.325120000	-0.037370000
C	3.180745000	-2.073842000	-0.598895000
H	3.332119000	-3.065209000	-0.138805000
H	3.208315000	-2.198896000	-1.693809000
H	4.029786000	-1.433191000	-0.307137000
C	1.564503000	-1.115377000	1.829665000
H	2.310161000	-0.359555000	2.127456000
H	0.583331000	-0.798890000	2.214034000
H	1.836556000	-2.064699000	2.319910000
C	0.106469000	-2.355800000	-0.660552000
H	0.273399000	-3.416028000	-0.406642000
H	-0.848080000	-2.042685000	-0.211780000
H	0.015320000	-2.285221000	-1.756795000
H	-4.508967000	-1.804081000	-0.160424000
F	-0.239310000	2.745800000	-0.760312000
F	0.223206000	2.021066000	1.350322000

E(RwB97XD) = -1013.56835979

Intermediate

C	1.162837000	0.514843000	-0.647589000
H	0.965308000	0.506940000	-1.732330000
B	-0.045877000	1.472218000	0.144470000
N	2.333250000	1.219271000	-0.488102000
N	3.238940000	1.810888000	-0.243210000
C	-1.446515000	0.696698000	0.048740000
C	-2.052734000	0.140080000	1.183889000
C	-2.090031000	0.518248000	-1.186129000
C	-3.245332000	-0.580676000	1.092491000
H	-1.579844000	0.273135000	2.160246000
C	-3.280541000	-0.200758000	-1.289924000
H	-1.647191000	0.945409000	-2.091242000
C	-3.859469000	-0.758517000	-0.147765000
H	-3.697656000	-1.006224000	1.991631000
H	-3.760842000	-0.329373000	-2.262965000
Si	1.429851000	-1.322926000	-0.015258000
C	3.099030000	-1.861533000	-0.687615000
H	3.298749000	-2.905703000	-0.394208000
H	3.125695000	-1.809047000	-1.788280000
H	3.921053000	-1.242974000	-0.290548000
C	1.430786000	-1.310778000	1.855293000

H	2.154663000	-0.581999000	2.252366000
H	0.435959000	-1.065747000	2.254617000
H	1.716142000	-2.309568000	2.225814000
C	0.041114000	-2.337359000	-0.753004000
H	-0.934863000	-2.079396000	-0.315968000
H	-0.015088000	-2.209655000	-1.845657000
H	0.239340000	-3.403341000	-0.548270000
H	-4.790146000	-1.325306000	-0.224607000
F	-0.046053000	2.718777000	-0.538970000
F	0.403828000	1.677903000	1.473362000

E(RwB97XD) = -1013.58060038

TS2

C	-0.880832000	-0.407891000	-0.562080000
H	-0.759991000	-0.493230000	-1.654850000
B	-0.039266000	-1.506685000	0.252986000
N	-2.647961000	-1.209545000	-0.609054000
N	-3.480539000	-1.861530000	-0.308193000
C	1.388913000	-0.735439000	0.065016000
C	1.988086000	-0.076299000	1.151900000
C	2.054300000	-0.707522000	-1.171577000
C	3.217038000	0.566538000	1.014840000
H	1.487273000	-0.085447000	2.122588000
C	3.278972000	-0.055108000	-1.319178000
H	1.611392000	-1.211588000	-2.035124000
C	3.862003000	0.583981000	-0.224599000
H	3.676615000	1.057301000	1.875860000
H	3.781239000	-0.046142000	-2.289031000
Si	-1.292451000	1.409989000	-0.025132000
C	-2.934597000	1.863263000	-0.821824000
H	-3.129499000	2.937345000	-0.664162000
H	-2.923482000	1.677285000	-1.908060000
H	-3.775385000	1.304486000	-0.380498000
C	-1.398865000	1.512032000	1.841803000
H	-2.115748000	0.777215000	2.240559000
H	-0.420654000	1.333855000	2.312963000
H	-1.741421000	2.517529000	2.138366000
C	0.089335000	2.464680000	-0.737129000
H	1.056643000	2.245770000	-0.259837000
H	0.191882000	2.310448000	-1.823077000
H	-0.144366000	3.529130000	-0.565093000
H	4.821181000	1.094423000	-0.335875000
F	-0.066695000	-2.788785000	-0.356195000
F	-0.423488000	-1.623068000	1.612850000

E(RwB97XD) = -1013.55389191

The computed reaction pathway for the coupling between 14 and TMS diazomethane 2

Start

B	-0.001985000	0.000573000	0.000825000
C	0.679802000	1.415839000	0.000631000
C	1.897326000	1.640755000	0.673225000
C	0.100054000	2.510061000	-0.671206000
C	2.499698000	2.897660000	0.688435000
H	2.376357000	0.815492000	1.206069000
C	0.711454000	3.762574000	-0.685839000
H	-0.843352000	2.372967000	-1.205703000
C	1.909721000	3.960106000	0.001980000
H	3.435154000	3.049057000	1.231385000
H	0.248873000	4.589942000	-1.228238000
H	2.384999000	4.943593000	0.002482000
C	0.884395000	-1.296675000	-0.000254000
C	0.480084000	-2.461077000	0.682076000
C	2.115845000	-1.341434000	-0.683054000
C	1.272068000	-3.608128000	0.697118000
H	-0.469222000	-2.464047000	1.223724000
C	2.898134000	-2.495115000	-0.699228000
H	2.461606000	-0.457272000	-1.224490000
C	2.480432000	-3.629144000	-0.001025000
H	0.943449000	-4.491845000	1.248310000
H	3.840646000	-2.508599000	-1.250886000
H	3.097653000	-4.530346000	-0.000727000
C	-1.568250000	-0.118710000	0.002569000
C	-2.375456000	0.823686000	0.670200000
C	-2.221944000	-1.171762000	-0.667335000
C	-3.764888000	0.713647000	0.681991000
H	-1.903540000	1.653199000	1.202950000
C	-3.612073000	-1.271351000	-0.686069000
H	-1.628179000	-1.921713000	-1.196258000
C	-4.386090000	-0.331098000	-0.004120000
H	-4.366626000	1.448710000	1.220976000
H	-4.094421000	-2.088133000	-1.227302000
H	-5.475341000	-0.412875000	-0.006984000

E(RwB97XD) = -719.649745592

TS1

C	-0.607753000	-0.193552000	-1.479280000
H	-0.196029000	0.702641000	-1.958209000

N	0.010912000	-1.267617000	-1.944230000
N	0.523681000	-2.232084000	-2.215950000
C	-0.434591000	0.989078000	1.254719000
C	-0.822389000	0.581262000	2.541497000
C	-1.053371000	2.145353000	0.740721000
C	-1.785752000	1.279181000	3.274623000
H	-0.361857000	-0.304083000	2.986726000
C	-2.021971000	2.843782000	1.456697000
H	-0.777015000	2.501902000	-0.255585000
C	-2.394115000	2.409501000	2.731815000
H	-2.063798000	0.935286000	4.273680000
H	-2.489820000	3.729825000	1.021192000
Si	-2.529439000	-0.342788000	-1.506589000
C	-3.126153000	1.246289000	-2.313270000
H	-4.222181000	1.221910000	-2.433160000
H	-2.874377000	2.123386000	-1.695624000
H	-2.676391000	1.383292000	-3.309950000
C	-2.927151000	-1.847750000	-2.567332000
H	-2.527071000	-1.748498000	-3.589743000
H	-2.513340000	-2.769771000	-2.125003000
H	-4.019822000	-1.977662000	-2.639646000
C	-3.344377000	-0.562034000	0.170624000
H	-3.035318000	-1.491364000	0.673394000
H	-3.160306000	0.284550000	0.847099000
H	-4.432921000	-0.629093000	-0.001213000
H	-3.152585000	2.953726000	3.298984000
B	0.684546000	0.208526000	0.431496000
C	1.046330000	-1.284702000	0.857001000
C	0.059406000	-2.269617000	1.054997000
C	2.376818000	-1.668961000	1.099090000
C	0.376264000	-3.564622000	1.457749000
H	-0.988295000	-2.014838000	0.879472000
C	2.706804000	-2.959599000	1.519666000
H	3.179513000	-0.939496000	0.966693000
C	1.707526000	-3.914662000	1.694986000
H	-0.416228000	-4.305081000	1.588967000
H	3.751677000	-3.219117000	1.705310000
H	1.962261000	-4.928072000	2.013235000
C	1.839844000	1.079900000	-0.254216000
C	2.667285000	0.564462000	-1.272508000
C	2.116288000	2.390962000	0.173610000
C	3.686426000	1.317960000	-1.851074000
H	2.517703000	-0.459186000	-1.624651000
C	3.146664000	3.150211000	-0.385732000
H	1.517871000	2.832635000	0.973560000
C	3.931110000	2.619413000	-1.407709000
H	4.299439000	0.885802000	-2.645323000

H	3.333934000	4.162808000	-0.020838000
H	4.733357000	3.211678000	-1.853375000

E(RwB97XD) = -1277.00387203

Intermediate

C	-0.516067000	-0.113938000	-1.375664000
H	-0.431113000	0.812535000	-1.968656000
N	0.156417000	-1.042951000	-2.133256000
N	0.719948000	-1.842157000	-2.658088000
C	-0.511332000	1.232938000	0.927069000
C	-0.770352000	1.080911000	2.299869000
C	-1.115800000	2.343601000	0.306580000
C	-1.584304000	1.967491000	3.008121000
H	-0.338349000	0.233373000	2.836816000
C	-1.937136000	3.235201000	0.997726000
H	-0.948865000	2.527823000	-0.758917000
C	-2.178933000	3.048959000	2.359182000
H	-1.761350000	1.805780000	4.074437000
H	-2.390158000	4.078510000	0.470451000
Si	-2.440861000	-0.576024000	-1.357149000
C	-3.269825000	0.787212000	-2.343250000
H	-4.352498000	0.587000000	-2.409270000
H	-3.137868000	1.768625000	-1.861343000
H	-2.871071000	0.843509000	-3.369038000
C	-2.629549000	-2.217120000	-2.257261000
H	-2.252055000	-2.166282000	-3.291771000
H	-2.127566000	-3.053528000	-1.744924000
H	-3.704002000	-2.461278000	-2.308455000
C	-3.141261000	-0.672923000	0.375651000
H	-2.535028000	-1.301567000	1.043638000
H	-3.242354000	0.325037000	0.826915000
H	-4.147476000	-1.120702000	0.302096000
H	-2.822644000	3.740289000	2.907748000
B	0.419441000	0.177864000	0.094346000
C	0.689569000	-1.209356000	0.904492000
C	0.034601000	-2.430601000	0.683782000
C	1.649389000	-1.207472000	1.937821000
C	0.306527000	-3.578083000	1.435908000
H	-0.721360000	-2.517718000	-0.100243000
C	1.927023000	-2.337742000	2.704154000
H	2.200515000	-0.285546000	2.146713000
C	1.253523000	-3.536263000	2.455614000
H	-0.228962000	-4.506232000	1.221390000
H	2.676796000	-2.286050000	3.497670000
H	1.470364000	-4.427666000	3.048479000

C	1.837920000	0.822230000	-0.425772000
C	2.873451000	-0.009819000	-0.899273000
C	2.094972000	2.204324000	-0.465228000
C	4.071981000	0.497846000	-1.402104000
H	2.750462000	-1.096259000	-0.857615000
C	3.292886000	2.727710000	-0.957983000
H	1.342789000	2.902267000	-0.090437000
C	4.287701000	1.875891000	-1.436545000
H	4.845143000	-0.186851000	-1.759879000
H	3.450980000	3.809245000	-0.962579000
H	5.226061000	2.280478000	-1.822806000

E(RwB97XD) = -1277.01320499

TS2

C	0.603910000	-0.261951000	-1.150299000
H	0.459967000	-1.231776000	-1.657165000
B	-0.449421000	-0.045142000	0.068022000
N	-0.070735000	0.605124000	-2.608345000
N	-0.673063000	1.142158000	-3.354956000
C	0.320676000	-1.202065000	0.990379000
C	1.237357000	-0.871116000	2.005529000
C	0.109241000	-2.574079000	0.759852000
C	1.887204000	-1.846497000	2.762184000
H	1.444698000	0.178668000	2.220400000
C	0.760509000	-3.559592000	1.502220000
H	-0.593881000	-2.885537000	-0.017115000
C	1.653938000	-3.198767000	2.510951000
H	2.583849000	-1.547364000	3.548929000
H	0.565988000	-4.614620000	1.295236000
Si	2.512153000	0.126165000	-1.264882000
C	2.876939000	0.576805000	-3.057396000
H	3.968536000	0.663622000	-3.190076000
H	2.514514000	-0.191038000	-3.760554000
H	2.432249000	1.545192000	-3.339153000
C	3.073280000	1.520321000	-0.145247000
H	2.625208000	2.482002000	-0.436769000
H	2.835595000	1.334688000	0.911926000
H	4.169053000	1.614676000	-0.235989000
C	3.360840000	-1.497641000	-0.843593000
H	3.218361000	-1.775480000	0.211528000
H	2.985029000	-2.319133000	-1.474492000
H	4.443832000	-1.397265000	-1.028709000
H	2.163677000	-3.965951000	3.098020000
C	-0.430268000	1.436125000	0.746945000
C	-0.178007000	2.611599000	0.020093000

C	-0.748155000	1.617615000	2.107735000
C	-0.224513000	3.881592000	0.601352000
H	0.072073000	2.558001000	-1.042173000
C	-0.802218000	2.876989000	2.705200000
H	-0.955805000	0.740457000	2.727253000
C	-0.536135000	4.021813000	1.952274000
H	-0.013234000	4.765052000	-0.006453000
H	-1.049819000	2.965661000	3.766020000
H	-0.572684000	5.010694000	2.414611000
C	-1.977854000	-0.510726000	-0.318825000
C	-2.284198000	-1.346532000	-1.409850000
C	-3.077328000	-0.109125000	0.463382000
C	-3.586916000	-1.757254000	-1.703444000
H	-1.488872000	-1.708698000	-2.068127000
C	-4.383745000	-0.514769000	0.188463000
H	-2.914602000	0.549631000	1.319117000
C	-4.649218000	-1.343358000	-0.901974000
H	-3.770097000	-2.404486000	-2.564928000
H	-5.201357000	-0.176103000	0.829935000
H	-5.670785000	-1.658796000	-1.125503000

E(RwB97XD) = -1276.99297293

TMSCHN$_2$ (**2**)

Si	0.677495000	-0.019597000	-0.000015000
C	1.683439000	0.396156000	-1.536786000
H	1.123008000	0.158919000	-2.455683000
H	1.939657000	1.468528000	-1.560663000
H	2.626688000	-0.175884000	-1.552496000
C	0.143095000	-1.823481000	-0.001382000
H	1.027691000	-2.482108000	-0.001686000
H	-0.456278000	-2.068227000	0.891056000
H	-0.455918000	-2.066909000	-0.894416000
C	1.683722000	0.393740000	1.537257000
H	1.939995000	1.466059000	1.562702000
H	1.123344000	0.155238000	2.455859000
H	2.626916000	-0.178416000	1.552043000
C	-0.898794000	1.012605000	0.000850000
H	-0.959685000	2.105153000	0.001602000
N	-2.054330000	0.425219000	0.000401000
N	-3.044116000	-0.136949000	-0.000080000

E(RwB97XD) = -557.345811590

CH$_2$N$_2$

C	-1.139346000	0.000001000	-0.000112000

H	-1.638609000	0.966519000	0.000185000
N	0.152948000	-0.000027000	0.000070000
N	1.291808000	0.000016000	-0.000026000
H	-1.638607000	-0.966449000	0.000185000

E(RwB97XD) = -148.693182186

N_2

N	0.000000000	0.000000000	0.550137000
N	0.000000000	0.000000000	-0.550137000

E(RwB97XD) = -109.489752742

7. References

1 R. Crampton, S. Woodward and M. Fox, *Adv. Synth. Catal.*, 2011, **353**, 903–906.

2 J. K. Awino, R. W. Gunasekara and Y. Zhao, *J. Am. Chem. Soc.*, 2016, **138**, 9759–9762.

3 D. S. W. Lim, T. T. S. Lew and Y. Zhang, *Org. Lett.*, 2015, **17**, 6054–6057.

4 T. Senda, M. Ogasawara and T. Hayashi, *J. Org .Chem*, 2001, **66**, 6852–6856.

5 M. K. Smith and B. H. Northrop, *Chem. Mater.*, 2014, **26**, 3781–3795.

6 H. G. Kuivila and A. R. Hendrickson, *J. Am. Chem. Soc.*, 1952, **74**, 5068–5070.

7 F. J. Williams and E. R. Jarvo, *Angew. Chemie - Int. Ed.*, 2011, **50**, 4459–4462.

8 C. Schrapel, W. Frey, D. Garnier and R. Peters, *Chem. - A Eur. J.*, 2016, 2448–2460.

9 C. Peng, W. Zhang, G. Yan and J. Wang, *Org. Lett.*, 2009, **11**, 1667–1670.

10 K. Endo, F. Kurosawa and Y. Ukaji, *Chem. Lett.*, 2013, **42**, 1363–1365.
